# A Useful Criterion on Studying Consistent Estimation in Community Detection

**DOI:** 10.3390/e24081098

**Published:** 2022-08-09

**Authors:** Huan Qing

**Affiliations:** School of Mathematics, China University of Mining and Technology, Xuzhou 221116, China; qinghuan@cumt.edu.cn

**Keywords:** community detection, consistency, mixed membership network, separation condition, sharp threshold

## Abstract

In network analysis, developing a unified theoretical framework that can compare methods under different models is an interesting problem. This paper proposes a partial solution to this problem. We summarize the idea of using a separation condition for a standard network and sharp threshold of the Erdös–Rényi random graph to study consistent estimation, and compare theoretical error rates and requirements on the network sparsity of spectral methods under models that can degenerate to a stochastic block model as a four-step criterion SCSTC. Using SCSTC, we find some inconsistent phenomena on separation condition and sharp threshold in community detection. In particular, we find that the original theoretical results of the SPACL algorithm introduced to estimate network memberships under the mixed membership stochastic blockmodel are sub-optimal. To find the formation mechanism of inconsistencies, we re-establish the theoretical convergence rate of this algorithm by applying recent techniques on row-wise eigenvector deviation. The results are further extended to the degree-corrected mixed membership model. By comparison, our results enjoy smaller error rates, lesser dependence on the number of communities, weaker requirements on network sparsity, and so forth. The separation condition and sharp threshold obtained from our theoretical results match the classical results, so the usefulness of this criterion on studying consistent estimation is guaranteed. Numerical results for computer-generated networks support our finding that spectral methods considered in this paper achieve the threshold of separation condition.

## 1. Introduction

Networks with latent structure are ubiquitous in our daily life, for example, social networks from social platforms, protein–protein interaction networks, co-citation networks and co-authorship networks [1,2,3,4,5,6,7,8,9,10,11,12,13,14,15]. Community detection is a powerful tool to learn the latent community structure in networks and graphs in social science, computer science, machine learning, statistical science and complex networks [16,17,18,19,20,21,22]. The goal of community detection is to infer a node’s community information from the network.

Many models have been proposed to model networks with latent community structure; see [23] for a survey. The stochastic blockmodel (SBM) [24] stands out for its simplicity, and it has received increasing attention in recent years [25,26,27,28,29,30,31,32,33,34,35]. However, the SBM only models a non-overlapping network in which each node belongs to a single community. Estimating mixed memberships of the network whose node may belong to multiple communities has received a lot of attention [36,37,38,39,40,41,42,43,44]. To capture the structure of the network with mixed memberships, Ref. [36] proposed the popular mixed membership stochastic blockmodel (MMSB), which is an extension of SBM from non-overlapping networks to overlapping networks. It is well known that the degree-corrected stochastic blockmodel (DCSBM) [45] is an extension of SBM by considering the degree heterogeneity of nodes to fit real-world networks with various node degree. Similarly, Ref. [41] proposed a model named the degree-corrected mixed membership (DCMM) model as an extension of MMSB by considering the degree heterogeneity of nodes. There are alternative models based on MMSB, such as the overlapping continuous community assignment model (OCCAM) of [40] and the stochastic blockmodel with overlap (SBMO) proposed by [46], which can also model networks with mixed memberships. As discussed in Section 5, OCCAM equals DCMM, while SBMO is a special case of DCMM.

### 1.1. Spectral Clustering Approaches

For the four models SBM, DCSBM, MMSB and DCMM, many researchers focus on designing algorithms with provable consistent theoretical guarantees. Spectral clustering [47] is one of the most widely applied methods with guarantees of consistency for community detection.

Within the SBM and DCSBM frameworks for a non-overlapping network, spectral clustering has two steps. It first conducts the eigen-decomposition of the adjacency matrix or the Laplacian matrix [26,48,49]. Then it runs a clustering algorithm (typically, k-means) on some leading eigenvectors or their variants to infer the community membership. For example, Ref. [26] showed the consistency of spectral clustering designed based on Laplacian matrix under SBM. Ref. [48] proposed a regularized spectral clustering (RSC) algorithm designed based on regularized Laplacian matrix and shows its theoretical consistency under DCSBM. Ref. [30] studied the consistencies of two spectral clustering algorithms based on the adjacency matrix under SBM and DCSBM. Ref. [50] designed the spectral clustering on the ratios-of-eigenvectors (SCORE) algorithm with a theoretical guarantee under DCSBM. Ref. [49] studied the impact of regularization on a Laplacian spectral clustering under SBM.

Within the MMSB and DCMM frameworks for the overlapping network, broadly speaking, spectral clustering has the following three steps. One first conducts an eigen-decomposition of the adjacency matrix or the graph Laplacian, then hunts corners (also known as vertexes) using a convex hull algorithm, and finally has a membership reconstruction step by projection. The convex hull algorithms suggested in [41] differ in the k-means algorithm a lot. For example, Ref. [44] designed the sequential projection after cleaning (SPACL) algorithm based on the finding that there exists a simplex structure in the eigen-decomposition of the population adjacency matrix and studies the SPACL theoretical properties under MMSB. Meanwhile, SPACL uses the successive projection algorithm proposed in [51] to find the corners for its simplex structure. To fit DCMM, Ref. [41] designs the Mixed-SCORE algorithm based on the finding that there exists a simplex structure in the entry-wise ratio matrix obtained from the eigen-decomposition of the population adjacency matrix under DCMM. Ref. [41] also introduces several choices for convex hull algorithms to find corners for the simplex structure and show the estimation consistency of the Mixed-SCORE under DCMM. Ref. [43] finds the cone structure inherent in the normalization of eigenvectors of the population adjacency matrix under DCMM as well as OCCAM, and develops an algorithm to hunt corners in the cone structure.

### 1.2. Separation Condition, Alternative Separation Condition and Sharp Threshold

SBM with *n* nodes belonging to *K* equal (or nearly equal) size communities and vertices connect with probability $p_{\mathrm{in}}$ within clusters and $p_{\mathrm{out}}$ across clusters, denoted by $SBM(n,K,p_{\mathrm{in}},p_{\mathrm{out}})$, has been well studied in recent years, especially for the case when K=2; see [21] and the references therein. In this paper, we call the network generated from $SBM(n,K,p_{\mathrm{in}},p_{\mathrm{out}})$ the standard network for convenience. Without causing confusion, we also call $SBM(n,K,p_{\mathrm{in}},p_{\mathrm{out}})$ the standard network, occasionally. Let $p_{\mathrm{in}}=\alpha_{\mathrm{in}}\frac{\log(n)}{n},p_{\mathrm{out}}=\alpha_{\mathrm{out}}\frac{\log(n)}{n}$. Refs. [21,52] found that exact recovery in $SBM(n,2,\alpha_{\mathrm{in}}\frac{\log(n)}{n},\alpha_{\mathrm{out}}\frac{\log(n)}{n})$ is solvable, and efficiently so, if $|\sqrt{\alpha_{\mathrm{in}}}-\sqrt{\alpha_{\mathrm{out}}}|>\sqrt{2}$ (i.e., $|\sqrt{p_{\mathrm{in}}}-\sqrt{p_{\mathrm{out}}}|>\sqrt{\frac{2\log(n)}{n}}$) and unsolvable if $|\sqrt{\alpha_{\mathrm{in}}}-\sqrt{\alpha_{\mathrm{out}}}|<\sqrt{2}$ as summarized in Theorem 13 of [53]. This threshold can be achieved by semidefinite relaxations [21,54,55,56] and spectral methods with local refinements [57,58]. Unlike semidefinite relaxations, spectral methods have a different threshold, which was particularly pointed out by [21,52]: one highlight for $SBM(n,2,p_{\mathrm{in}},p_{\mathrm{out}})$ is a theorem by [59] which says that when $p_{\mathrm{in}}>p_{\mathrm{out}}$, if

$$\begin{array}{c} \frac{p_{\mathrm{in}}-p_{\mathrm{out}}}{\sqrt{p_{\mathrm{in}}}}\gg \sqrt{\frac{\log(n)}{n}} \end{array}$$

then spectral methods can exactly recover node labels with high probability as *n* goes to infinity (also known as consistent estimation [30,40,41,43,44,48,50]).

Consider a more general case $SBM(n,K,p_{\mathrm{in}},p_{\mathrm{out}})$ with K=O(1); this paper finds that the above threshold can be extended as

(1) $$\begin{array}{c} \frac{|p_{\mathrm{in}}-p_{\mathrm{out}}|}{\sqrt{\max(p_{\mathrm{in}},p_{\mathrm{out}})}}\gg \sqrt{\frac{\log(n)}{n}}, \end{array}$$

which can be alternatively written as

(2) $$\begin{array}{c} \frac{|\alpha_{\mathrm{in}}-\alpha_{\mathrm{out}}|}{\sqrt{\max(\alpha_{\mathrm{in}},\alpha_{\mathrm{out}})}}\gg 1. \end{array}$$

In this paper, when K=O(1), the lower bound requirement on $\frac{|p_{\mathrm{in}}-p_{\mathrm{out}}|}{\sqrt{\max(p_{\mathrm{in}},p_{\mathrm{out}})}}$ (and $\frac{|\alpha_{\mathrm{in}}-\alpha_{\mathrm{out}}|}{\sqrt{\max(\alpha_{\mathrm{in}},\alpha_{\mathrm{out}})}}$) for the consistent estimation of spectral methods is called the separation condition (alternative separation condition). The network generated from $SBM(n,K,p_{\mathrm{in}},p_{\mathrm{out}})$ with $p_{\mathrm{in}}>p_{\mathrm{out}}$ is an assortative network in which nodes within the community have more edges than across communities [60]. The network generated from $SBM(n,K,p_{\mathrm{in}},p_{\mathrm{out}})$ with $p_{\mathrm{in}}<p_{\mathrm{out}}$ is a dis-assortative network in which nodes within the community have fewer edges than across communities [60]. Therefore, Equation (2) holds for both assortative and dis-assortative networks.

Meanwhile, when K=1 such that $p=p_{\mathrm{in}}=p_{\mathrm{out}}$, $SBM\left(n,K,p_{\mathrm{in}},p_{\mathrm{out}}\right)=SBM\left(n,1,p,p\right)$ degenerates to Erdös–Rényi (ER) random graph G(n,p) [53,61,62]. Ref. [61] finds that the ER random graph is connected with high probability if

(3) $$\begin{array}{c} p\geq \frac{\log(n)}{n}. \end{array}$$

We call the lower bound requirement on *p* for generating a connected ER random graph the sharp threshold in this paper.

### 1.3. Inconsistencies on Separation Condition in Some Previous Works

In this paper, we focus on the consistency of spectral method in community detection. The study of consistency is developed by obtaining the theoretical upper bound of error rate for a spectral method through analyzing the properties of the population adjacency matrix under the statistical model. To compare the consistencies of the theoretical results under different models, it is meaningful to study whether the separation condition and sharp threshold obtained from upper bounds of theoretical error rates for different methods under different models are consistent or not. Meanwhile, the separation condition and sharp threshold can also be seen as alternative unified theoretical frameworks to compare all methods and model parameters mentioned in the concluding remarks of [30].

Based on the separation condition and sharp threshold, here we describe some phenomena of the inconsistency in the community detection area. We find that the separation conditions of $SBM(n,K,p_{\mathrm{in}},p_{\mathrm{out}})$ with K=O(1) obtained from the error rates developed in [41,43,44] under DCMM or MMSB are not consistent with those obtained from the main results of [30] under SBM, and the sharp threshold obtained from the main results of [43,44] do not match the classical results. A summary of these inconsistencies is provided in Table 1 and Table 2. Furthermore, after delicate analysis, we find that the requirement on the network sparsity of [43,44] is stronger than that of [30,41], and [63] also finds that the requirement of Ref. [44] of network sparsity is sub-optimal.

### 1.4. Our Findings

Recall that we reviewed several spectral clustering methods under SBM, DCSBM, MMSB and DCMM introduced in [26,30,41,43,44,48,49,50] and DCSBM, MMSB and DCMM are extensions of SBM (i.e., $SBM(n,K,p_{\mathrm{in}},p_{\mathrm{out}})$ is a special case of DCSBM, MMSB and DCMM). We have the following question:

Can these spectral clustering methods achieve the threshold in Equation (1) (or Equation (2)) for $SBM(n,K,p_{\mathrm{in}},p_{\mathrm{out}})$ with K=O(1) and the threshold in Equation (3) for the Erdös–Rényi (ER) random graph G(n,p)?

The answer is yes. In fact, spectral methods for network with mixed memberships still achieve thresholds in Equations (1) and (2) for $MMSB(n,K,\Pi ,p_{\mathrm{in}},p_{\mathrm{out}})$ defined in Definition 2 when K=O(1), where $MMSB(n,K,\Pi ,p_{\mathrm{in}},p_{\mathrm{out}})$ can be seen as a generalization of $SBM(n,K,p_{\mathrm{in}},p_{\mathrm{out}})$ such that there exist nodes belonging to multiple communities. Explanations for why these spectral clustering methods achieve thresholds in Equations (1)–(3) will be provided in Section 3, Section 4 and Section 5 via re-establishing theoretical guarantee for SPACL under MMSB and its extension under DCMM because we find that the main theoretical results of [43,44] are sub-optimal. Meanwhile, we can obtain (and cannot obtain) the separation condition and sharp threshold from the theoretical bounds of error rates for spectral methods analyzed in [30,41,43,44] ([26,30,48,49,50]) directly. Instead of re-establishing the theoretical guarantee for all spectral methods reviewed in this paper to show that they achieve thresholds in Equations (1) and (3) for $SBM(n,K,P_{\mathrm{in}},P_{\mathrm{out}})$ with K=O(1), we mainly focus on the SPACL algorithm under MMSB and its extension under DCMM since MMSB and DCMM are more complex than SBM and DCSBM.

We then summarize the idea of using the separation condition and sharp threshold to study the consistencies, and compare the error rates and requirements on network sparsity of different spectral methods under different models as a four-step criterion, which we call the separation condition and sharp threshold criterion (SCSTC for short). With an application of this criterion, this paper provides an attempt to answer the questions of how the above inconsistency phenomena occur, and how to obtain consistent resultswith weaker requirements on the network sparsity of [43,44]. To answer the two questions, we use the recent techniques on row-wise eigenvector deviation developed in [64,65] to obtain consistent theoretical results directly related with model parameters for the SPACL and the SVM-cone-DCMMSB algorithm of [43]. The two questions are then answered by delicate analysis with an application of SCSTC to the theoretical upper bounds of error rates in this paper and some previous spectral methods. Using SCSTC for the spectral methods introduced and studied in [26,30,48,49,50] and some other spectral methods fitting models that can reduce to $SBM(n,K,P_{\mathrm{in}},P_{\mathrm{out}})$ with K=O(1), one can prove that these spectral methods achieve thresholds in Equations (1)–(3). The main contributions in this paper are as follows:

(i) We summarize the idea of using the separation condition of a standard network and sharp threshold of the ER random graph G(n,p) to study the consistent estimations of different spectral methods designed via eigen-decomposition or singular value decomposition of the adjacency matrix or its variants under different models that can degenerate to SBM under mild conditions as a four-step criterion, SCSTC. The separation condition is used to study the consistency of the theoretical upper bound for the spectral method, and the sharp threshold can be used to study the network sparsity. The theoretical results of upper bounds for different spectral methods can be compared by SCSTC. Using this criterion, a few inconsistent phenomenons of some previous works are found.
(ii) Under MMSB and DCMM, we study the consistencies of the SPACL algorithm proposed in [44] and its extended version using the recent techniques on row-wise eigenvector deviation developed in [64,65]. Compared with the original results of [43,44], our main theoretical results enjoy smaller error rates by lesser dependence on *K* and log(n). Meanwhile, our main theoretical results have weaker requirements on the network sparsity and the lower bound of the smallest nonzero singular value of the population adjacency matrix. For details, see Table 3 and Table 4.
(iii) Our results for DCMM are consistent with those for MMSB when DCMM degenerates to MMSB under mild conditions. Using SCSTC, under mild conditions, our main theoretical results under DCMM are consistent with those of [41]. This answers the question that the phenomenon that the main results of [43,44] do not match those of [41] occurs due to the fact that in Refs. [43,44], the theoretical results of error rates are sub-optimal. We also find that our theoretical results (as well as those of [41]) under both MMSB and DCMM match the classical results on the separation condition and sharp threshold, i.e., achieve thresholds in Equations (1)–(3). Using the bound of ∥A−Ω∥ instead of $∥A_{\mathrm{re}}-\Omega ∥$ to establish the upper bound of error rate under SBM in [30], the two spectral methods studied in [30] achieve thresholds in Equations (1)–(3), which answers the question of why the separation condition obtained from error rate of [41] does not match that obtained from the error rate of [30]. Using $∥A_{\mathrm{re}}-\Omega ∥$ or ∥A−Ω∥ influences the row-wise eigenvector deviations in Theorem 3.1 of [44] and Theorem I.3 of [43], and thus using $∥A_{\mathrm{re}}-\Omega ∥$ or ∥A−Ω∥ influences the separation conditions and sharp thresholds of [43,44]. For comparison, our bound on row-wise eigenvector deviation is obtained by using the techniques developed in [64,65] and that of [41] is obtained by applying the modified Theorem 2.1 of [66]; therefore, using $∥A_{\mathrm{re}}-\Omega ∥$ or ∥A−Ω∥ has no influence on the separation conditions and sharp thresholds of ours and that of [41]. For details, see Table 1 and Table 2. In a word, using SCSTC, the spectral methods proposed and studied in [26,30,41,43,44,48,49,50,67,68] or some other spectral methods fitting models that can reduce to $SBM(n,K,p_{\mathrm{in}},p_{\mathrm{out}})$ achieve thresholds in Equations (1)–(3).
(iv) We verify our threshold in Equation (2) by some computer-generated networks in Section 6. The numerical results for networks generated under $MMSB(n,K,\Pi ,p_{\mathrm{in}},p_{\mathrm{out}})$ when K=2 and K=3 show that SPACL and its extended version achieve a threshold in Equation (2), and results for networks generated from $SBM(n,K,p_{\mathrm{in}},p_{\mathrm{out}})$ when K=2 and K=3 show that the spectral methods considered in [26,30,48,50] achieve the threshold in Equation (2).

The article is organized as follows. In Section 2, we give the formal introduction to the mixed membership stochastic blockmodel and review the algorithm SPACL considered in this paper. The theoretical results of consistency for the mixed membership stochastic blockmodel are presented and compared to related works in Section 3. After delicate analysis, the separation condition and sharp threshold criterion is presented in Section 4. Based on an application of this criterion, the improvement consistent estimation results for the extended version of SPACL under the degree corrected mixed membership model are provided in Section 5. Several computer-generated networks under MMSB and SBM are conducted to show that some spectral clustering methods achieve the threshold in Equation (2) in Section 6. The conclusion is given in Section 7.

*Notations.* We take the following general notations in this paper. Write [m]:={1,2,…,m} for any positive integer *m*. For a vector *x* and fixed q>0, ${∥x∥}_{q}$ denotes its $l_{q}$-norm. We drop the subscript if q=2 occasionally. For a matrix *M*, $M^{\prime }$ denotes the transpose of the matrix *M*, ∥M∥ denotes the spectral norm, ${∥M∥}_{F}$ denotes the Frobenius norm, ${∥M∥}_{2\to \infty }$ denotes the maximum $l_{2}$-norm of all the rows of *M*, and ${∥M∥}_{\infty }:=\max_{i}\sum_{j}\left|M\left(i,j\right)\right|$ denotes the maximum absolute row sum of *M*. Let rank(M) denote the rank of matrix *M*. Let $\sigma_{i}\left(M\right)$ be the *i*-th largest singular value of matrix *M*, $\lambda_{i}\left(M\right)$ denote the *i*-th largest eigenvalue of the matrix *M* ordered by the magnitude, and κ(M) denote the condition number of *M*. M(i,:) and M(:,j) denote the *i*-th row and the *j*-th column of matrix *M*, respectively. $M(S_{r},:)$ and $M(:,S_{c})$ denote the rows and columns in the index sets $S_{r}$ and $S_{c}$ of matrix *M*, respectively. For any matrix *M*, we simply use Y=max(0,M) to represent $Y_{ij}=\max\left(0,M_{ij}\right)$ for any i,j. For any matrix $M\in R^{m\times m}$, let diag(M) be the m×m diagonal matrix whose *i*-th diagonal entry is M(i,i). 1 and 0 are column vectors with all entries being ones and zeros, respectively. $e_{i}$ is a column vector whose *i*-th entry is 1, while other entries are zero. In this paper, *C* is a positive constant which may vary occasionally. f(n)=O(g(n)) means that there exists a constant c>0 such that |f(n)|≤c|g(n)| holds for all sufficiently large *n*. x⪰y means there exists a constant c>0 such that |x|≥c|y|. f(n)=o(g(n)) indicates that $\frac{f(n)}{g(n)}\to 0$ as n→∞.

## 2. Mixed Membership Stochastic Blockmodel

Let $A\in {\left\{0,1\right\}}^{n\times n}$ be a symmetric adjacency matrix such that A(i,j)=1 if there is an edge between node *i* to node *j*, and A(i,j)=0 otherwise. The mixed membership stochastic blockmodel (MMSB) [36] for generating *A* is as follows.

(4) $$\begin{array}{c} \Omega :=\rho \Pi \tilde{P}\Pi^{\prime }\;\;\;\;\;\;\;\;\;A\left(i,j\right)∼\mathrm{Bernoulli}\left(\Omega \left(i,j\right)\right)\;\;\;\;i,j\in \left[n\right], \end{array}$$

where $\Pi \in R^{n\times K}$ is called the membership matrix with Π(i,k)≥0 and $\sum_{k=1}^{K}\Pi \left(i,k\right)=1$ for i∈[n] and k∈[K], $\tilde{P}\in R^{K\times K}$ is an non-negative symmetric matrix with $\max_{k,l\in [K]}\tilde{P}\left(k,l\right)=1$ for model identifiability under MMSB, ρ is called the sparsity parameter which controls the sparsity of the network, and $\Omega \in R^{n\times n}$ is called the population adjacency matrix since E[A]=Ω. As mentioned in [41,44], $\sigma_{K}\left(\tilde{P}\right)$ is a measure of the separation between communities, and we call it the separation parameter in this paper. ρ and $\sigma_{K}\left(\tilde{P}\right)$ are two important model parameters directly related with the separation condition and sharp threshold, and they will be considered throughout this paper.

**Definition** **1.** *Call model (4) the mixed membership stochastic blockmodel (MMSB), and denote it by $MMSB_{n}\left(K,\tilde{P},\Pi ,\rho \right)$.*

**Definition** **2.** *Let $MMSB(n,K,\Pi ,p_{\mathrm{in}},p_{\mathrm{out}})$ be a special case of $MMSB_{n}\left(K,\tilde{P},\Pi ,\rho \right)$ when $\rho \tilde{P}$ has diagonal entries $p_{\mathrm{in}}$ and non-diagonal entries $p_{\mathrm{out}}$, and $\kappa \left(\Pi^{\prime }\Pi \right)=O\left(1\right)$.*

Call node *i* ‘pure’ if Π(i,:) is degenerate (i.e., one entry is 1, all others K−1 entries are 0) and ‘mixed’ otherwise. When all nodes are pure in Π, we see that $MMSB(n,K,\Pi ,p_{\mathrm{in}},p_{\mathrm{out}})$ exactly reduces to $SBM(n,K,p_{\mathrm{in}},p_{\mathrm{out}})$. Thus, $MMSB(n,K,\Pi ,p_{\mathrm{in}},p_{\mathrm{out}})$ is a generalization of $SBM(n,K,p_{\mathrm{in}},p_{\mathrm{out}})$ with mixed nodes in each community. In this paper, we show that SPACL [44] fitting MMSB and SVM-cone-DCMMSB [43] and Mixed-SCORE [41] fitting DCMM also achieve thresholds in Equations (1)–(3) for $MMSB(n,K,\Pi ,p_{\mathrm{in}},p_{\mathrm{out}})$ with K=O(1). By Theorems 2.1 and 2.2 [44], the following conditions are sufficient for the identifiability of MMSB, when $\rho \tilde{P}\left(k,l\right)\in \left[0,1\right]$ for all k,l∈[K],

- (I1) $\mathrm{rank}(\tilde{P})=K$.
- (I2) There is at least one pure node for each of the *K* communities.

Unless specified, we treat conditions (I1) and (I2) as the default from now on.

For k∈[K], let $I^{\left(k\right)}$ be the set of pure nodes in community *k* such that $I^{\left(k\right)}=\left\{i\in \left[n\right]:\Pi \left(i,k\right)=1\right\}$. For k∈[K], select one node from $I^{\left(k\right)}$ to construct the index set I, i.e., I is the index of nodes corresponding to *K* pure nodes, one from each community. Without loss of generality, let $\Pi \left(I,:\right)=I_{K}$ where $I_{K}$ is the K×K identity matrix. Recall that rank(Ω)=K. Let $\Omega =U\Lambda U^{\prime }$ be the compact eigen-decomposition of Ω such that $U\in R^{n\times K},\Lambda \in R^{K\times K}$, and $U^{\prime }U=I_{K}$. Lemma 2.1 [44] gives that U=ΠU(I,:), and such a form is called ideal simplex (IS for short) [41,44] since all rows of *U* form a *K*-simplex in $R^{K}$ and the *K* rows of U(I,:) are the vertices of the *K*-simplex. Given Ω and *K*, as long as we know U(I,:), we can exactly recover Π by $\Pi =UU^{-1}\left(I,:\right)$ since $U\left(I,:\right)\in R^{K\times K}$ is a full rank matrix. As mentioned in [41,44], for such IS, the successive projection (SP) algorithm [51] (i.e., Algorithm A1) can be applied to *U* with *K* communities to exactly find the corner matrix U(I,:). For convenience, set $Z=UU^{-1}\left(I,:\right)$. Since Π=Z, we have $\Pi \left(i,:\right)=\frac{Z(i,:)}{{∥Z\left(i,:\right)∥}_{1}}$ for i∈[n].

Based on the above analysis, we are now ready to give the ideal SPACL algorithm with input Ω,K and output Π.

- Let $\Omega =U\Lambda U^{\prime }$ be the top-*K* eigen-decomposition of Ω such that $U\in R^{n\times K},\Lambda \in R^{K\times K},U^{\prime }U=I$.
- Run SP algorithm on the rows of *U* assuming that there are *K* communities to obtain I.
- Set $Z=UU^{-1}\left(I,:\right)$.
- Recover Π by setting $\Pi \left(i,:\right)=\frac{Z(i,:)}{{∥Z\left(i,:\right)∥}_{1}}$ for i∈[n].

With the given *U* and *K*, since the SP algorithm returns U(I,:), we see that the ideal SPACL exactly (for detail, see Appendix B) returns Π.

Now, we review the SPACL algorithm of [44]. Set $\tilde{A}=\hat{U}\hat{\Lambda }{\hat{U}}^{\prime }$ to be the top *K* eigen-decomposition of *A* such that $\hat{U}\in R^{n\times K},\hat{\Lambda }\in R^{K\times K},{\hat{U}}^{\prime }\hat{U}=I_{K}$, and $\hat{\Lambda }$ contains the top *K* eigenvalues of *A*. For the real case, use $\hat{Z},\hat{\Pi }$ given in Algorithm 1 to estimate Z,Π, respectively. Algorithm 1 is the SPACL algorithm [44] where we only care about the estimation of the membership matrix Π, and omit the estimation of *P* and ρ. Meanwhile, Algorithm 1 is a direct extension of the ideal SPACL algorithm from the oracle case to the real case, and we omit the prune step in the original SPACL algorithm of [44].
**Algorithm 1** SPACL [44]**Require:** The adjacency matrix $A\in R^{n\times n}$ and the number of communities *K*.**Ensure:** The estimated n×K membership matrix $\hat{\Pi }$.1: Obtain $\tilde{A}=\hat{U}\hat{\Lambda }{\hat{U}}^{\prime }$, the top *K* eigen-decomposition of *A*.2: Apply SP algorithm (i.e., Algorithm A1) on the rows of $\hat{U}$ assuming there are *K* communities to obtain $\hat{I}$, the index set returned by SP algorithm.3: Set $\hat{Z}=\hat{U}{\hat{U}}^{-1}\left(\hat{I},:\right)$. Then set $\hat{Z}=\max\left(0,\hat{Z}\right)$.4: Estimate Π(i,:) by $\hat{\Pi }\left(i,:\right)=\hat{Z}\left(i,:\right)/{∥\hat{Z}\left(i,:\right)∥}_{1},i\in \left[n\right]$.

## 3. Consistency under MMSB

Our main result under MMSB provides an upper bound on the estimation error of each node’s membership in terms of several model parameters. Throughout this paper, *K* is a known positive integer. Assume that

(A1) ρn≥log(n).

Assumption (A1) provides a requirement on the lower bound of sparsity parameter ρ such that it should be at least log(n)/n. Then we have the following lemma.

**Lemma** **1.** *Under $MMSB_{n}\left(K,\tilde{P},\Pi ,\rho \right)$, when Assumption (A1) holds, with probability at least $1-o(n^{-\alpha })$ for any α>0, we have*

$$\begin{array}{c} ∥A-\Omega ∥\leq \frac{\alpha +1+\sqrt{\left(\alpha +1)(\alpha +19\right)}}{3}\sqrt{\rho n\log(n)}. \end{array}$$

In Lemma 1, instead of simply using a constant $C_{\alpha }$ to denote $\frac{\alpha +1+\sqrt{\left(\alpha +1)(\alpha +19\right)}}{3}$, we keep the explicit form here.

**Remark** **1.** *When Assumption (A1) holds, the upper bound of ∥A−Ω∥ in Lemma 1 is consistent with Corollary 6.5 in [69] since Var(A(i,j))≤ρ under $MMSB_{n}\left(K,P,\Pi ,\rho \right)$.*

Lemma 1 is obtained via Theorem 1.4 (Bernstein inequality) in [70]. For comparison, Ref. [44] applies Theorem 5.2 [30] to bound ∥A−Ω∥ (see, for example, Equation (14) of [44]) and obtains a bound as $C\sqrt{\rho n}$ for some C>0. However, $C\sqrt{\rho n}$ is the bound between a regularization of *A* and Ω as stated in the proof of Theorem 5.2 [30], where such regularization of *A* is obtained from *A* with some constraints in Lemmas 4.1 and 4.2 of the supplemental material [30]. Meanwhile, Theorem 2 [71] also gives that the bound between a regularization of *A* and Ω is $C\sqrt{\rho n}$, where such a regularization of *A* should also satisfy few constraints on *A*; see Theorem 2 [71] for detail. Instead of bounding the difference between a regularization of *A* and Ω, we are interested in bounding ∥A−Ω∥ by the Bernstein inequality, which has no constraints on *A*. For convenience, use $A_{\mathrm{re}}$ to denote the regularization of *A* in this paper. Hence, $∥A_{\mathrm{re}}-\Omega ∥\leq C\sqrt{\rho n}$ with high probability, and this bound is model independent as shown by Theorem 5.2 [30] and Theorem 2 [71] as long as $\rho \geq \max_{i,j}\Omega \left(i,j\right)$ (here, let Ω=E[A] without considering models, a ρ satisfying $\rho \geq \max_{i,j}\Omega \left(i,j\right)$ is also the sparsity parameter which controls the overall sparsity of a network). Note that $A_{\mathrm{re}}$ is not $\tilde{A}$, where $\tilde{A}=\hat{U}\Lambda {\hat{U}}^{\prime }$ is obtained by the top *K* eigen-decomposition of *A*, while $A_{\mathrm{re}}$ is obtained by adding constraints on degrees of *A*; see Theorem 2 [71] for detail.

In [41,43,44], the main theoretical results for their proposed membership estimating methods hinge on a row-wise deviation bound for the eigenvectors of the adjacency matrix, whether under MMSB or DCMM. Different from the theoretical technique applied in Theorem 3.1 [44], which provides sup-optimal dependencies on log(n) and *K*, and needs sub-optimal requirements on the sparsity parameter ρ and the lower bound of $\sigma_{K}\left(\Omega \right)$, to obtain row-wise deviation bound for the singular eigenvector of Ω, we use Theorem 4.2 [64] and Theorem 4.2 [65].

**Lemma** **2** (Row-wise eigenspace error). *Under $MMSB_{n}\left(K,\tilde{P},\Pi ,\rho \right)$, when Assumption (A1) holds, suppose $\sigma_{K}\left(\Omega \right)\geq C\sqrt{\rho n\log(n)}$, with probability at least $1-o(n^{-\alpha })$,*

- *When we apply Theorem 4.2 of [64], we have*
$$\begin{array}{c} ∥\hat{U}{\hat{U}}^{\prime }-UU^{\prime }{∥}_{2\to \infty }=O\left(\frac{\sqrt{K}\left(\kappa \left(\Omega \right)\sqrt{\frac{n}{K\lambda_{K}\left(\Pi^{\prime }\Pi \right)}}+\sqrt{\log(n)}\right)}{\sigma_{K}\left(\tilde{P}\right)\sqrt{\rho }\lambda_{K}\left(\Pi^{\prime }\Pi \right)}\right), \end{array}$$
- *When we apply Theorem 4.2 of [65], we have*
$$\begin{array}{c} ∥\hat{U}{\hat{U}}^{\prime }-UU^{\prime }{∥}_{2\to \infty }=O\left(\frac{\sqrt{n\log(n)}}{\sigma_{K}\left(\tilde{P}\right)\sqrt{\rho }\lambda_{K}^{1.5}\left(\Pi^{\prime }\Pi \right)}\right). \end{array}$$

For convenience, set $\varpi =∥\hat{U}{\hat{U}}^{\prime }-UU^{\prime }{∥}_{2\to \infty }$, and let $\varpi_{1},\varpi_{2}$ denote the upper bound in Lemma 2 when applying Theorem 4.2 of [64] and Theorem 4.2 of [65], respectively. Note that when $\lambda_{K}\left(\Pi^{\prime }\Pi \right)=O\left(\frac{n}{K}\right)$, we have $\varpi_{1}=\varpi_{2}=O\left(\frac{K^{1.5}}{\sigma_{K}\left(\tilde{P}\right)}\frac{1}{\sqrt{n}}\sqrt{\frac{\log(n)}{\rho n}}\right)$, and therefore we simply let $\varpi_{2}$ be the bound since its form is slightly simpler than $\varpi_{1}$.

Compared with Theorem 3.1 of [44], since we apply Theorem 4.2 of [64] and Theorem 4.2 of [65] to obtain the bound of row-wise eigenspace error under MMSB, our bounds do not rely on $\min(K^{2},\kappa^{2}\left(\Omega \right))$ while Theorem 3.1 [44] does. Meanwhile, our bound in Lemma 2 is sharper with lesser dependence on *K* and log(n), has weaker requirements on the lower bounds of $\sigma_{K}\left(\Omega \right),\lambda_{K}\left(\Pi^{\prime }\Pi \right)$ and the sparsity parameter ρ. The details are given below:

- We emphasize that the bound of Theorem 3.1 of [44] should be $∥\hat{U}{\hat{U}}^{\prime }-UU^{\prime }{∥}_{2\to \infty }=O\left(\frac{\psi \left(\Omega \right)\sqrt{Kn}\log^{\xi }\left(n\right)}{\sigma_{K}\left(\tilde{P}\right)\sqrt{\rho }\lambda_{K}^{1.5}\left(\Pi^{\prime }\Pi \right)}\right)$ instead of $∥\hat{U}{\hat{U}}^{\prime }-UU^{\prime }{∥}_{2\to \infty }=O\left(\frac{\psi \left(\Omega \right)\sqrt{Kn}}{\sigma_{K}\left(\tilde{P}\right)\sqrt{\rho }\lambda_{K}^{1.5}\left(\Pi^{\prime }\Pi \right)}\right)$ for ξ>1 where the function ψ is defined in Equation (7) of [44], and this is also pointed out by Table 2 of [63]. The reason is that in the proof part of Theorem 3.1 [44], from step (iii) to step (iv), they should keep the term $\log^{\xi }\left(n\right)$ since this term is much larger than 1. We can also find that the bound in Theorem 3.1 [44] should multiply $\log^{\xi }\left(n\right)$ from Theorem VI.1 [44] directly. For comparison, this bound $O(\frac{\psi \left(\Omega \right)\sqrt{Kn}\log^{\xi }\left(n\right)}{\sigma_{K}\left(\tilde{P}\right)\sqrt{\rho }\lambda_{K}^{1.5}\left(\Pi^{\prime }\Pi \right)})$ is $K^{0.5}\log^{\xi -0.5}\left(n\right)$ times our bound in Lemma 2. Meanwhile, by the proof of the bound in Theorem 3.1 of [44], we see that the bound depends on the upper bound of ∥A−Ω∥, and [44] applies Theorem 5.2 of [30] such that $∥A_{\mathrm{re}}-\Omega ∥\leq C\sqrt{\rho n}$ with high probability. Since $C\sqrt{\rho n}$ is the upper bound of the difference between a regularization of *A* and Ω. Therefore, if we are only interested in bounding ∥A−Ω∥ instead of $∥A_{\mathrm{re}}-\Omega ∥$, the upper bound of Theorem 3.1 [44] should be $O(\frac{\psi \left(\Omega \right)\sqrt{Kn}\log^{\xi +0.5}\left(n\right)}{\sigma_{K}\left(\tilde{P}\right)\lambda_{K}^{1.5}\left(\Pi^{\prime }\Pi \right)})$, which is at least $K^{0.5}\log^{\xi }\left(n\right)$ times our bound in Lemma 2. Furthermore, the upper bound of the row-wise eigenspace error in Lemma 2 does not rely on the upper bound of ∥A−Ω∥ as long as $\sigma_{K}\left(\Omega \right)\geq C\sqrt{\rho n\log(n)}$ holds. Therefore, whether using $∥A_{\mathrm{re}}-\Omega ∥\leq C\sqrt{\rho n}$ or $∥A-\Omega ∥\leq C\sqrt{\rho n\log(n)}$ does not change the bound in Lemma 2.
- Our Lemma 2 requires $\sigma_{K}\left(\Omega \right)\geq C\sqrt{\rho n\log(n)}$, while Theorem 3.1 [44] requires $\sigma_{K}\left(\Omega \right)\geq 4\sqrt{\rho n}\log^{\xi }\left(n\right)$ by their Assumption 3.1. Therefore, our Lemma 2 has a weaker requirement on the lower bound of $\sigma_{K}\left(\Omega \right)$ than that of Theorem 3.1 [44]. Meanwhile, Theorem 3.1 [44] requires $\lambda_{K}\left(\Pi^{\prime }\Pi \right)\geq \frac{1}{\rho }$ while our Lemma 2 has no lower bound requirement on $\lambda_{K}\left(\Pi^{\prime }\Pi \right)$ as long as it is positive.
- Since $∥\Omega ∥=∥\rho \Pi \tilde{P}\Pi^{\prime }∥\leq C\rho n$ by basic algebra, the lower bound requirement on $\sigma_{K}\left(\Omega \right)$ in Assumption 3.1 of [44] gives that $4\sqrt{\rho n}\log^{\xi }\left(n\right)\leq \sigma_{K}\left(\Omega \right)\leq ∥\Omega ∥\leq C\rho n$, which suggests that Theorem 3.1 [44] requires $\rho n\geq C\log^{2\xi }\left(n\right)$, and this also matches with the requirement on ρn in Theorem VI.1 of [44] (and this is also pointed out by Table 1 of [63]). For comparison, our requirement on sparsity given in Assumption (A1) is ρn≥log(n), which is weaker than $\rho n\geq C\log^{2\xi }\left(n\right)$. Similarly, in our Lemma 2, the requirement $\sigma_{K}\left(\Omega \right)\geq C\sqrt{\rho n\log(n)}$ gives $C\sqrt{\rho n\log(n)}\leq \sigma_{K}\left(\Omega \right)\leq ∥\Omega ∥\leq C\rho n$, thus we have log(n)≤Cρn which is consistent with Assumption (A1).

If we further assume that $K=O\left(1\right),\lambda_{K}\left(\Pi^{\prime }\Pi \right)=O\left(\frac{n}{K}\right)$ (i.e., $\kappa \left(\Pi^{\prime }\Pi \right)=O\left(1\right)$) and $\sigma_{K}\left(\tilde{P}\right)=O\left(1\right)$, the row-wise eigenspace error is of order $\frac{1}{\sqrt{n}}\sqrt{\frac{\log(n)}{\rho n}}$, which is consistent with the row-wise eigenvector deviation of the result of [63], shown in their Table 2. The next theorem gives the theoretical bounds on the estimations of memberships under MMSB.

**Theorem** **1.** *Under $MMSB_{n}\left(K,\tilde{P},\Pi ,\rho \right)$, let $\hat{\Pi }$ be obtained from Algorithm 1, and suppose the conditions in Lemma 2 hold; there exists a permutation matrix $P\in R^{K\times K}$ such that, with probability at least $1-o(n^{-\alpha })$, we have*

$$\begin{array}{c} \max_{i\in [n]}{∥e_{i}^{\prime }\left(\hat{\Pi }-\Pi P\right)∥}_{1}=O\left(\varpi K\kappa \left(\Pi^{\prime }\Pi \right)\sqrt{\lambda_{1}\left(\Pi^{\prime }\Pi \right)}\right). \end{array}$$

**Remark** **2** (Comparison to Theorem 3.2 [44]). *Consider a special case by setting $\kappa \left(\Pi^{\prime }\Pi \right)=O\left(1\right)$, i.e., $\lambda_{K}\left(\Pi^{\prime }\Pi \right)=O\left(\frac{n}{K}\right)$ and $\lambda_{1}\left(\Pi^{\prime }\Pi \right)=O\left(\frac{n}{K}\right)$. We focus on comparing the dependencies on K in bounds of our Theorem 1 and Theorem 3.2 [44]. Under this case, the bound of our Theorem 1 is proportional to $K^{2}$ by basic algebra; since $\min\left(K^{2},\kappa^{2}\left(\Omega \right)\right)=\min\left(K^{2},O\left(1\right)\right)=O\left(1\right)$ and the bound in Theorem 3.2 [44] should multiply $\sqrt{K}$ because (in [44]’s language) $∥{\hat{V}}_{p}^{-1}{∥}_{F}\leq \frac{\sqrt{K}}{\sigma_{K}\left({\hat{V}}_{p}\right)}$ instead of $∥{\hat{V}}_{p}^{-1}{∥}_{F}=\frac{1}{\lambda_{K}\left({\hat{V}}_{p}\right)}$ in Equation (45) [44], the power of K is 2 by checking the bound of Theorem 3.2 [44]. Meanwhile, note that our bound in Theorem 2 is $l_{1}$ bound, while the bound in Theorem 3.2 [44] is $l_{2}$ bound. When we translate the $l_{2}$ bound of Theorem 3.2 [44] into $l_{1}$ bound, the power of K is 2.5 for Theorem 3.2 [44]. Hence, our bound in Theorem 1 has less dependence on K than that of Theorem 3.2 [44], and this is also consistent with the first bullet given after Lemma 2.*

Table 3 summarizes the necessary conditions and dependence on the model parameters of the rates in Theorem 1 and Theorem 3.2 [44] for comparison. The following corollary is obtained by adding conditions on the model parameters similar to Corollary 3.1 in [44].

**Corollary** **1.** *Under $MMSB(n,K,\Pi ,p_{\mathrm{in}},p_{\mathrm{out}})$ with K=O(1), when the conditions of Lemma 2 hold, with probability at least $1-o(n^{-\alpha })$, we have*

$$\begin{array}{c} \max_{i\in [n]}{∥e_{i}^{\prime }\left(\hat{\Pi }-\Pi P\right)∥}_{1}=O\left(\frac{1}{\sigma_{K}\left(\tilde{P}\right)}\sqrt{\frac{\log(n)}{\rho n}}\right). \end{array}$$

**Remark** **3.** *Consider a special case in Corollary 1 by setting $\sigma_{K}\left(\tilde{P}\right)$ as a constant, we see that the error bound $O(\sqrt{\frac{\log(n)}{\rho n}})$ in Corollary 1 is directly related to Assumption (A1), and for consistent estimation, ρ should shrink slower than $\frac{\log(n)}{n}$.*

**Remark** **4.** *Under the setting of Corollary 1, the requirement $\sigma_{K}\left(\Omega \right)\geq C\sqrt{\rho n\log(n)}$ in Lemma 2 holds naturally. By Lemma II.4 [44], we know that $\sigma_{K}\left(\Omega \right)\geq \rho \sigma_{K}\left(\tilde{P}\right)\lambda_{K}\left(\Pi^{\prime }\Pi \right)=C\rho n\sigma_{K}\left(\tilde{P}\right)$. To make the requirement $\sigma_{K}\left(\Omega \right)\geq C\sqrt{\rho n\log(n)}$ always hold, we just need $C\rho n\sigma_{K}\left(\tilde{P}\right)\geq C\sqrt{\rho n\log(n)}$, which gives that $\sigma_{K}\left(\tilde{P}\right)\geq C\sqrt{\frac{\log(n)}{\rho n}}$, and it just matches with the requirement of the consistent estimation of memberships in Corollary 1.*

**Remark** **5** (Comparison to Theorem 3.2 [44]). *When K=O(1) and $\lambda_{K}\left(\Pi^{\prime }\Pi \right)=O\left(\frac{n}{K}\right)$, by the first bullet in the analysis given after Lemma 2, the row-wise eigenspace error of Theorem 3.1 [44] is $O(\frac{\log^{\xi }\left(n\right)}{\sigma_{K}\left(\tilde{P}\right)\sqrt{\rho }n})$, and it gives that their error bound on estimation membership given in their Equation (3) is $O(\frac{\log^{\xi }\left(n\right)}{\sigma_{K}\left(\tilde{P}\right)\sqrt{\rho n}})$, which is $\log^{\xi -0.5}\left(n\right)$ times of the bound in our Lemma 1.*

**Remark** **6** (Comparison to Theorem 2.2 [41]). *Replacing the *Θ* in [41] by $\Theta =\sqrt{\rho }I$, their DCMM model degenerates to MMSB. Then their conditions in Theorem 2.2 are our Assumption (A1) and $\lambda_{K}\left(\Pi^{\prime }\Pi \right)=O\left(\frac{n}{K}\right)$ for MMSB. When K=O(1), the error bound in Theorem 2.2 in [41] is $O(\frac{1}{\sigma_{K}\left(\tilde{P}\right)}\sqrt{\frac{\log(n)}{\rho n}})$, which is consistent with ours.*

## 4. Separation Condition and Sharp Threshold Criterion

After obtaining Corollary 1 under MMSB, now we are ready to give our criterion after introducing the separation condition of $MMSB(n,K,\Pi ,p_{\mathrm{in}},p_{\mathrm{out}})$ with K=O(1) and the sharp threshold of ER random graph G(n,p) in this section.

*Separation condition.* Let $P=\rho \tilde{P}$ be the probability matrix for $MMSB(n,K,\Pi ,p_{\mathrm{in}},p_{\mathrm{out}})$ when K=O(1), so *P* has diagonal (and non-diagonal) entries $p_{\mathrm{in}}$ (and $p_{\mathrm{out}}$) and $\sigma_{K}\left(P\right)=\rho \sigma_{K}\left(\tilde{P}\right)\equiv \left|p_{\mathrm{in}}-p_{\mathrm{out}}\right|$. Recall that $\max_{k,l\in [K]}\tilde{P}\left(k,l\right)=1$ under $MMSB_{n}\left(K,\tilde{P},\Pi ,\rho \right)$, we have $\max_{k,l\in [K]}P\left(k,l\right)=\rho \equiv \max\left(p_{\mathrm{in}},p_{\mathrm{out}}\right)$. So, we have the separation condition $\frac{|p_{\mathrm{in}}-p_{\mathrm{out}}|}{\sqrt{\max(p_{\mathrm{in}},p_{\mathrm{out}})}}\equiv \sqrt{\rho }\sigma_{K}\left(\tilde{P}\right)$ (also known as the relative edge probability gap in [44]) and the alternative separation condition $\frac{|\alpha_{\mathrm{in}}-\alpha_{\mathrm{out}}|}{\sqrt{\max(\alpha_{\mathrm{in}},\alpha_{\mathrm{out}})}}\equiv \sqrt{\frac{\rho n}{\log(n)}}\sigma_{K}\left(\tilde{P}\right)$. Now, we are ready to compare the thresholds of the (alternative) separation condition obtained from different theoretical results.

- (a) By Corollary 1, we know that $\sigma_{K}\left(\tilde{P}\right)$ should shrink slower than $\sqrt{\frac{\log(n)}{\rho n}}$ for consistent estimation. Therefore, the separation condition $\frac{|p_{\mathrm{in}}-p_{\mathrm{out}}|}{\sqrt{\max(p_{\mathrm{in}},p_{\mathrm{out}})}}\equiv \sqrt{\rho }\sigma_{K}\left(\tilde{P}\right)$ should shrink slower than $\sqrt{\frac{\log(n)}{n}}$ (i.e., Equation (1)), and this threshold is consistent with Corollary 1 of [59] and Equation (17) of [49]. The alternative separation condition $\frac{|\alpha_{\mathrm{in}}-\alpha_{\mathrm{out}}|}{\sqrt{\max(\alpha_{\mathrm{in}},\alpha_{\mathrm{out}})}}\equiv \sqrt{\frac{\rho n}{\log(n)}}\sigma_{K}\left(\tilde{P}\right)$ should shrink slower than 1 (i.e., Equation (2)).
- (b) Undoubtedly, the (alternative) separation condition in (a) is consistent with that of [41], since Theorem 2.2 [41] shares the same error rate $O(\frac{1}{\sigma_{K}\left(\tilde{P}\right)}\sqrt{\frac{\log(n)}{\rho n}})$ for $MMSB(n,K,\Pi ,p_{\mathrm{in}},p_{\mathrm{out}})$ with K=O(1).
- (c) By Remark 5, using $∥A_{\mathrm{re}}-\Omega ∥\leq C\sqrt{\rho n}$, we know that in Ref. [44], Equation (3) is $O(\frac{\log^{\xi }\left(n\right)}{\sigma_{K}\left(\tilde{P}\right)\sqrt{\rho n}})$, so $\sqrt{\rho }\sigma_{K}\left(\tilde{P}\right)$ should shrink slower than $\frac{\log^{\xi }\left(n\right)}{\sqrt{n}}$. Thus, for [44], the separation condition is $\frac{\log^{\xi }\left(n\right)}{\sqrt{n}}$, and the alternative separation condition is $\log^{\xi -0.5}\left(n\right)$, which are sub-optimal compared with ours in (a). Using $∥A-\Omega ∥\leq C\sqrt{\rho n\log(n)}$, and Equation (3) in Ref. [44], which is $O(\frac{\log^{\xi +0.5}\left(n\right)}{\sigma_{K}\left(\tilde{P}\right)\sqrt{\rho n}})$, we see that for [44], now the separation condition is $\frac{\log^{\xi +0.5}\left(n\right)}{\sqrt{n}}$ and the alternative separation condition is $\log^{\xi }\left(n\right)$.
- (d) For comparison, the error bound of Corollary 3.2 [30] built under SBM for community detection is $O(\frac{1}{\sigma_{K}^{2}\left(\tilde{P}\right)\rho n})$ for $SBM(n,K,p_{\mathrm{in}},p_{\mathrm{out}})$ with K=O(1), so $\sqrt{\rho }\sigma_{K}\left(\tilde{P}\right)$ should shrink slower than $\frac{1}{\sqrt{n}}$. Thus the separation condition for [30] is $\frac{1}{\sqrt{n}}$. However, as we analyzed in the first bullet given after Lemma 2, [30] applied $∥A_{re}-\Omega ∥\leq C\sqrt{\rho n}$ to build their consistency results. Instead, we apply $∥A-\Omega ∥\leq C\sqrt{\rho n\log(n)}$ to the built theoretical results of [30], and the error bound of Corollary 3.2 [30] is $O(\frac{\log(n)}{\sigma_{K}^{2}\left(\tilde{P}\right)\rho n})$, which returns the same separation condition as our Corollary 1 and Theorem 2.2 of [41] now. Following a similar analysis to (a)–(c), we can obtain an alternative separation condition for [30] immediately, and the results are provided in Table 2. Meanwhile, as analyzed in the first bullet given after Lemma 2, whether using $∥A-\Omega ∥\leq C\sqrt{\rho n\log(n)}$ or $∥A_{\mathrm{re}}-\Omega ∥\leq C\sqrt{\rho n}$ does not change our error rates. By carefully analyzing the proof of 2.1 of [41], we see that whether using $∥A-\Omega ∥\leq C\sqrt{\rho n\log(n)}$ or $∥A_{\mathrm{re}}-\Omega ∥\leq C\sqrt{\rho n}$ also does not change their row-wise large deviation, hence it does not influence their upper bound of the error rate for their Mixed-SCORE.

*Sharp threshold.* Consider the Erdös–Rényi (ER) random graph G(n,p) [61]. To construct the ER random graph G(n,p), let K=1 and Π be an n×1 vector with all entries being ones. Since K=1 and the maximum entry of $\tilde{P}$ is assumed to be 1, we have $\tilde{P}=1$ in G(n,p) and hence $\sigma_{K}\left(\tilde{P}\right)=1$. Then we have $\Omega =\Pi \rho \tilde{P}\Pi^{\prime }=\Pi \rho \Pi^{\prime }=\Pi p\Pi^{\prime }$, i.e, p=ρ. Since the error rate is $O\left(\frac{1}{\sigma_{K}\left(\tilde{P}\right)}\sqrt{\frac{\log(n)}{\rho n}}\right)=O\left(\sqrt{\frac{\log(n)}{pn}}\right)$, for consistent estimation, we see that *p* should shrink slower than $\frac{\log(n)}{n}$ (i.e., Equation (3)), which is just the sharp threshold in [61], Theorem 4.6 [62], strongly consistent with [72], and the first bullet in Section 2.5 [53] (called the lower bound requirement of *p* for the ER random graph to enjoy consistent estimation as the sharp threshold). Since the sharp threshold is obtained when K=1, which means a connected ER random graph G(n,p), this is also consistent with the connectivity in Table 2 of [21]. Meanwhile, since our Assumption (A1) requires ρn≥log(n), it gives that *p* should shrink slower than $\frac{\log(n)}{n}$ since p=ρ under G(n,p), which is consistent with the sharp threshold. Since Theorem 2.2 of Ref. [41] enjoys the same error rate as ours under the settings in Corollary 1, [41] also reaches the sharp threshold as $\frac{\log(n)}{n}$. Furthermore, Remark 5 says that the bound for the error rate in Equation (3) [44] should be $O(\frac{\log^{\xi }\left(n\right)}{\sigma_{K}\left(\tilde{P}\right)\sqrt{\rho n}})$ when using $∥A_{\mathrm{re}}-\Omega ∥\leq C\sqrt{\rho n}$; following a similar analysis, we see that the sharp threshold for [44] is $\frac{\log^{2\xi }\left(n\right)}{n}$, which is sub-optimal compared with ours. When using $∥A-\Omega ∥\leq C\sqrt{\rho n\log(n)}$, the sharp threshold for [44] is $\frac{\log^{2\xi +1}\left(n\right)}{n}$. Similarly, the error bound of Corollary 3.2 [30] is $O\left(\frac{1}{\sigma_{K}^{2}\left(\tilde{P}\right)\rho n}\right)\equiv O\left(\frac{1}{pn}\right)$ under ER G(n,p) since $p=\rho ,\sigma_{K}\left(\tilde{P}\right)=1$ and K=1. Hence, the sharp threshold obtained from the theoretical upper bound for error rates of [30] is $\frac{1}{n}$, which does not match the classical result. Instead, we apply $∥A-\Omega ∥\leq C\sqrt{\rho n\log(n)}$ with a high probability to build the theoretical results of [30], and the error bound of Corollary 3.2 [30] is $O(\frac{\log(n)}{pn})$, which returns the classical sharp threshold $\frac{\log(n)}{n}$ now.

Table 1 summarizes the comparisons of the separation condition and sharp threshold. Table 2 records the respective alternative separation condition. The delicate analysis given above supports our statement that the separation condition of a standard network (i.e., $SBM(n,K,p_{\mathrm{in}},p_{\mathrm{out}})$ with K=O(1) or $MMSB(n,K,\Pi ,p_{\mathrm{in}},p_{\mathrm{out}})$ with K=O(1)) and the sharp threshold of ER random graph G(n,p) can be seen as unified criteria to compare the theoretical results of spectral methods under different models. To conclude the above analysis, here, we summarize the main steps to apply the separation condition and sharp threshold criterion (SCSTC for short) to check the consistency of the theoretical results or compare the results of spectral methods under different models, where spectral methods mean methods developed based on the application of the eigenvectors or singular vectors of the adjacency matrix or its variants for community detection. The four-stage SCSTC is given below:

$step_{1}$ Check whether the theoretical upper bound of the error rate contains $\sigma_{K}\left(\tilde{P}\right)$ (note that $P=\rho \tilde{P}$ is probability matrix and maximum entries of $\tilde{P}$ should be set as 1), where the separation parameter $\sigma_{K}\left(\tilde{P}\right)$ always appears when considering the lower bound of $\sigma_{K}\left(\Omega \right)$. If it contains $\sigma_{K}\left(\tilde{P}\right)$, move to the next step. Otherwise, it suggests possible improvements for the consistency by considering $\sigma_{K}\left(\tilde{P}\right)$ in the proofs.
$step_{2}$ Let K=O(1) and network degenerate to the standard network whose numbers of nodes in each community are in the same order and can been seen as $O(\frac{n}{K})$ (i.e., a $SBM(n,K,p_{\mathrm{in}},p_{\mathrm{out}})$ with K=O(1) in the case of a non-overlapping network or a $MMSB(n,K,\Pi ,p_{\mathrm{in}},p_{\mathrm{out}})$ with K=O(1) in the case of an overlapping network, and we will mainly focus on $SBM(n,K,p_{\mathrm{in}},p_{\mathrm{out}})$ with K=O(1) for convenience.). Let the model degenerate to $SBM(n,K,p_{\mathrm{in}},p_{\mathrm{out}})$ with K=O(1), and then we obtain the new theoretical upper bound of the error rate. Note that if the model does consider degree heterogeneity, sparsity parameter ρ should be considered in the theoretical upper bound of error rate in $step_{1}$. If the model considers degree heterogeneity, when it degenerates to $SBM(n,K,p_{\mathrm{in}},p_{\mathrm{out}})$ with K=O(1), ρ appears at this step. Meanwhile, if ρ is not contained in the error rate of $step_{1}$ when the model does not consider degree heterogeneity, it suggests possible improvements by considering ρ.
$step_{3}$ Let $P=\rho \tilde{P}$ be the probability matrix when the model degenerates to $SBM(n,K,p_{\mathrm{in}},p_{\mathrm{out}})$ such that *P* has diagonal entries $p_{\mathrm{in}}$ and non-diagonal entries $p_{\mathrm{out}}$. So, $\sigma_{K}\left(P\right)=\left|p_{\mathrm{in}}-p_{\mathrm{out}}\right|=\rho \sigma_{K}\left(\tilde{P}\right)$ and separation condition $\frac{|p_{\mathrm{in}}-p_{\mathrm{out}}|}{\sqrt{\max(p_{\mathrm{in}},p_{\mathrm{out}})}}\equiv \sqrt{\rho }\sigma_{K}\left(\tilde{P}\right)$ since the maximum entry of $\tilde{P}$ is assumed to be 1. Compute the lower bound requirement of $\sigma_{K}\left(\tilde{P}\right)$ for consistency estimation through analyzing the new bound obtained in $step_{2}$. Compute separation condition $\frac{|p_{\mathrm{in}}-p_{\mathrm{out}}|}{\sqrt{\max(p_{\mathrm{in}},p_{\mathrm{out}})}}\equiv \sqrt{\rho }\sigma_{K}\left(\tilde{P}\right)$ using the lower bound requirement for $\sigma_{K}\left(\tilde{P}\right)$. The sharp threshold for the ER random graph G(n,p) is obtained from the lower bound requirement on ρ for the consistency estimation under the setting that $K=1,\sigma_{K}\left(\tilde{P}\right)=1$ and p=ρ.
$step_{4}$ Compare the separation condition and the sharp threshold obtained in $step_{3}$ with Equations (1) and (3), respectively. If the sharp threshold $\gg \frac{\log(n)}{n}$ or the separation condition $\gg \sqrt{\frac{\log(n)}{n}}$, then this leaves improvements on the requirement of the network sparsity or theoretical upper bound of the error rate. If the sharp threshold is $\frac{\log(n)}{n}$ and the separation condition is $\sqrt{\frac{\log(n)}{n}}$, the optimality of the theoretical results on both error rates and the requirement of network sparsity is guaranteed. Finally, if the sharp threshold $\ll \frac{\log(n)}{n}$ or separation condition $\ll \sqrt{\frac{\log(n)}{n}}$, this suggests that the theoretical result is obtained based on $∥A_{\mathrm{re}}-\Omega ∥$ instead of ∥A−Ω∥.

**Remark** **7.** *This remark provides some explanations on the four steps of SCSTC.*

- *In $step_{1}$, we give a few examples. When applying SCSTC to the main results of [40,48,67], we stop at $step_{1}$ as analyzed in Remark 8, suggesting possible improvements by considering $\sigma_{K}\left(\tilde{P}\right)$ for these works. Meanwhile, for the theoretical result without considering $\sigma_{K}\left(\tilde{P}\right)$, we can also move to $step_{2}$ to obtain the new theoretical upper bound of the error rate, which is related with ρ and n. Discussions on the theoretical upper bounds of error rates of [50,68] given in Remark 8 are examples of this case.*
- *In $step_{2}$, letting K=O(1) and the model reduce to $SBM(n,K,p_{\mathrm{in}},p_{\mathrm{out}})$ for the non-overlapping network or $MMSB(n,K,\Pi ,p_{\mathrm{in}},p_{\mathrm{out}})$ for the overlapping network can always simplify the theoretical upper bound of error rate, as shown by our Corollaries 1 and 2. Here, we provide some examples about how to make a model degenerate to SBM. For $MMSB_{n}\left(K,\tilde{P},\Pi ,\rho \right)$ in this paper, when all nodes are pure, MMSB degenerates to SBM; for the $DCMM_{n}\left(K,\tilde{P},\Pi ,\Theta \right)$ model introduced in Section 5 or DCSBM considered in [30,48,50], setting $\Theta =\sqrt{\rho }I$ makes DCMM and DCSBM degenerates to SBM when all nodes are pure, similar to the ScBM and DCScBM considered in [67,68,71], the OCCAM model of [40], the stochastic blockmodel with the overlap proposed in [46], the extensions of SBM and DCSBM for hypergraph networks considered in [73,74,75], and so forth.*
- *In $step_{3}$ and $step_{4}$, the separation condition can be replaced by an alternative separation condition.*
- *When using SCSTC to build and compare theoretical results for the spectral clustering method, the key point is computing the lower bound for $\frac{|p_{\mathrm{in}}-p_{\mathrm{out}}|}{\sqrt{\max(p_{\mathrm{in}},p_{\mathrm{out}})}}$ when the probability matrix P has diagonal entries $p_{\mathrm{in}}$ and non-diagonal entries $p_{\mathrm{out}}$ from the theoretical upper bound of the error rate for a certain spectral method. If this lower bound is consistent with that of Equation (1), this suggests theoretical optimality, and otherwise it suggests possible improvements by following the four steps of SCSTC.*

The above analysis shows that SCSTC can be used to study the consistent estimation of model-based spectral methods. Use SCSTC, the following remark lists a few works whose main theoretical results leave possible improvements.

**Remark** **8.** *The unknown separation condition, or sub-optimal error rates, or a lack of requirement of network sparsity of some previous works, suggest possible improvements of their theoretical results. Here, we list a few works whose main results can be possibly improved until considering the separation condition.*

- *Theorem 4.4 of [48] proposes the upper bound of the error rate for their regularized spectral clustering (RSC) algorithm, designed based on a regularized Laplacian matrix under DCSBM. However, since [48] does not study the lower bound (in the [48] language) of $\lambda_{K}$ and m, we cannot directly obtain the separation condition from their main theorem. Meanwhile, the main result of [48] does not consider the requirement on the network sparsity, which leaves some improvements. Ref. [48] does not study the theoretical optimal choice for the RSC regularizer τ. After considering $\sigma_{K}\left(\tilde{P}\right)$ and sparsity parameter ρ, one can obtain the theoretical optimal choice for τ, and this is helpful for explaining and choosing the empirical optimal choice for τ. Therefore, the feasible network implementation of SCSTC is obtaining the theoretical optimal choices for some tuning parameters, such as regularizer τ of the RSC algorithm. By using SCSTC, we can find that RSC achieves thresholds in Equations (1)–(3), and we omit proofs for it in this paper.*
- *Refs. [26,49] study two algorithms designed based on the Laplacian matrix and its regularized version under SBM. They obtain meaningful results, but do not consider the network sparsity parameter ρ and separation parameter $\sigma_{K}\left(\tilde{P}\right)$. After obtaining improved error bounds which are consistent with separation condition $\sqrt{\frac{\log(n)}{n}}$ using SCSTC, one can also obtain the theoretical optimal choice for regularizer τ of the RSC-τ algorithm considered in [49] and find that the two algorithms considered in [26,49] achieve thresholds in Equations (1)–(3).*
- *Theorem 2.2 of [50] provides an upper bound of their SCORE algorithm under DCSBM. However, since they do not consider the influence of $\sigma_{K}\left(\tilde{P}\right)$, we cannot directly obtain the separation condition from their main result. Meanwhile, by setting their $\Theta =\sqrt{\rho }I$, DCSBM degenerates to SBM, which gives that their $err_{n}=\frac{1}{\rho^{2}n}\left(1+\frac{\log(n)}{\rho n}\right)=O\left(\frac{1}{\rho^{2}n}\right)$ by their assumption Equation (2.9). Hence, when $\Theta =\sqrt{\rho }I$, the upper bound of Theorem 2.2 in [50] is $O(\frac{\log^{3}\left(n\right)}{\rho^{2}n})$. The upper bound of error rate in Corollary 3.2 of [30] is $O(\frac{\log(n)}{\rho n})$ when using $∥A-\Omega ∥\leq C\sqrt{\rho n\log(n)}$ under the setting that κ(Π)=O(1),K=O(1) and $\sigma_{K}\left(\tilde{P}\right)=O\left(1\right)$. We see that $\frac{\log{}^{3}\left(n\right)}{\rho^{2}n}$ grows faster than $\frac{\log(n)}{\rho n}$, which suggests that there is space to improve the main result of [50] in the aspects of the separation condition and error rates. Furthermore, using SCSTC, we can find that SCORE achieves thresholds in Equations (1)–(3) because its extension mixed-SCORE [41] achieves thresholds in Equations (1)–(3).*
- *Ref. [67] proposes two models, ScBM and DCScBM, to model the directed networks and an algorithm DI-SIM based on the directed regularized Laplacian matrix to fit DCScBM. However, similar to [48], their main theoretical result in their Theorem C.1 does not consider the lower bound of (in the language of Ref. [67]) $\sigma_{K},m_{y},m_{z}$ and $\gamma_{z}$, which causes that we cannot obtain the separation condition when DCScBM degenerates to SBM. Meanwhile, their Theorem C.1 also lacks a lower bound requirement on network sparsity. Hence, there is space to improve the theoretical guarantees of [67]. Similar to [48,49], we can also obtain the theoretical optimal choices for regularizer τ of the DI-SIM algorithm and prove that DI-SIM achieves the thresholds in Equations (1)–(3) since it is the directed version of RSC [48].*
- *Ref. [68] mainly studies the theoretical guarantee for the D-SCORE algorithm proposed by [14] to fit a special case of the DCScBM model for directed networks. By setting their $\theta \left(i\right)=\sqrt{\rho },\delta \left(j\right)=\sqrt{\rho }$ for i,j∈[n], their directed-DCBM degenerates to SBM. Meanwhile, since their $err_{n}=\frac{1}{\rho }$, their mis-clustering rate is $O(\frac{T_{n}^{2}\log\left(n\right)}{\rho n})$, which matches that of [30] under SBM when setting $T_{n}$ as a constant. However, if setting $T_{n}$ as log(n), then the error rate is $O(\frac{\log^{3}\left(n\right)}{\rho n})$, which is sub-optimal compared with that of [30]. Meanwhile, similar to [50,68], the main result does not consider the influences of K and $\sigma_{K}\left(\tilde{P}\right)$, causing a lack of a separation condition. Hence, the main results of [68] can be improved by considering K, $\sigma_{K}\left(P\right)$, or a more optimal choice of $T_{n}$ to make their main results comparable with those of [30] when directed-DCBM degenerates to SBM. Using SCSTC, we can find that the D-SCORE also achieves thresholds in Equations (1)–(3) since it is the directed version of SCORE [50].*

## 5. Degree Corrected Mixed Membership Model

Using SCSTC to Theorem 3.2 of [43], as shown in Table 1 and Table 2 results in Theorem 3.2 [43] being sub-optimal. To obtain the improvement theoretical results, we give a formal introduction of the degree corrected mixed membership (DCMM) model proposed in [41] first, then we review the SVM-cone-DCMMSB algorithm of [43] and provide the improvement theoretical results. A DCMM for generating *A* is as follows.

(5) $$\begin{array}{c} \Omega :=\Theta \Pi \tilde{P}\Pi^{\prime }\Theta \;\;\;\;\;\;\;\;\;A\left(i,j\right)∼\mathrm{Bernoulli}\left(\Omega \left(i,j\right)\right)\;\;\;\;i,j\in \left[n\right], \end{array}$$

where $\Theta \in R^{n\times n}$ is a diagonal matrix whose *i*-th diagonal entry is the degree heterogeneity of node *i* for i∈[n]. Let $\theta \in R^{n\times 1}$ with θ(i)=Θ(i,i) for i∈[n]. Set $\theta_{\max}=\max_{i\in [n]}\theta \left(i\right),\theta_{\min}=\min_{i\in [n]}\theta \left(i\right)$ and ${\tilde{P}}_{\max}=\max_{k,l\in [K]}\tilde{P}\left(k,l\right),{\tilde{P}}_{\min}=\min_{k,l\in [K]}\tilde{P}\left(k,l\right)$.

**Definition** **3.** *Call model (5) the degree corrected mixed membership (DCMM) model, and denote it by $DCMM_{n}\left(K,\tilde{P},\Pi ,\Theta \right)$.*

Note that if we set $\tilde{\Pi }=\Theta \Pi$ and choose Θ such that $\tilde{\Pi }\in {\left\{0,1\right\}}^{n\times K}$, then we have $\Omega =\tilde{\Pi }\tilde{P}{\tilde{\Pi }}^{\prime }$, which means that the stochastic blockmodel with overlap (SBMO) proposed in [46] is just a special case of DCMM. Meanwhile, if we write Θ as $\Theta =\tilde{\Theta }D_{o}$, where $\tilde{\Theta },D_{o}$ are two positive diagonal matrices and let $\Pi_{o}=D_{o}\Pi$, then we can choose $D_{0}$ such that $∥\Pi_{o}{\left(i,:\right)∥}_{F}=1$. By $\Omega =\Theta \Pi \tilde{P}\Pi^{\prime }\Theta =\tilde{\Theta }\Pi_{o}\tilde{P}\Pi_{o}^{\prime }\tilde{\Theta }$, we see that the OCCAM model proposed in [40] equals the DCMM model. By Equation (1.3) and Proposition 1.1 of [41], the following conditions are sufficient for the identifiability of DCMM when $\theta_{\max}{\tilde{P}}_{\max}\leq 1$:

- (II1) $\mathrm{rank}(\tilde{P})=K$ and $\tilde{P}$ has unit diagonals.
- (II2) There is at least one pure node for each of the *K* communities.

Note that though the diagonal entries of $\tilde{P}$ are ones, ${\tilde{P}}_{\max}$ may be larger than 1 as long as $\theta_{\max}{\tilde{P}}_{\max}\leq 1$ under DCMM, and this is slightly different from the setting that $\max_{k,l\in [K]}\tilde{P}\left(k,l\right)=1$ under MMSB.

Without causing confusion, under $DCMM_{n}\left(K,\tilde{P},\Pi ,\Theta \right)$, we still let $\Omega =U\Lambda U^{\prime }$ be the top-*K* eigen-decomposition of Ω such that $U\in R^{n\times K},\Lambda \in R^{K\times K}$ and $U^{\prime }U=I_{K}$. Set $U_{*}\in R^{n\times K}$ by $U_{*}\left(i,:\right)=\frac{U(i,:)}{{∥U\left(i,:\right)∥}_{F}}$ and let $N_{U}\in R^{n\times n}$ be a diagonal matrix such that $N_{U}\left(i,i\right)=\frac{1}{{∥U\left(i,:\right)∥}_{F}}$ for i∈[n]. Then $U_{*}$ can be rewritten as $U_{*}=N_{U}U$. The existence of the ideal cone (IC for short) structure inherent in $U_{*}$ mentioned in [43] is guaranteed by the following lemma.

**Lemma** **3.** Under $DCMM_{n}\left(K,\tilde{P},\Pi ,\Theta \right)$, $U_{*}=YU_{*}\left(I,:\right)$ where $Y=N_{M}\Pi \Theta^{-1}\left(I,I\right)N_{U}^{-1}\left(I,I\right)$ with $N_{M}$ being an n×n diagonal matrix whose diagonal entries are positive.

Lemma 3 gives $Y=U_{*}U_{*}^{-1}\left(I,:\right)$. Since $U_{*}=N_{U}U$ and $Y=N_{M}\Pi \Theta^{-1}\left(I,I\right)N_{U}^{-1}\left(I,I\right)$, we have

(6) $$\begin{array}{c} N_{U}^{-1}N_{M}\Pi =UU_{*}^{-1}\left(I,:\right)N_{U}\left(I,I\right)\Theta \left(I,I\right). \end{array}$$

Since $\Omega \left(I,I\right)=\Theta \left(I,I\right)\Pi \left(I,:\right)\tilde{P}\Pi^{\prime }\left(I,:\right)=\Theta \left(I,I\right)\tilde{P}\Theta \left(I,I\right)=U\left(I,:\right)\Lambda U^{\prime }\left(I,:\right)$, we have $\Theta \left(I,I\right)\tilde{P}\Theta \left(I,I\right)=U\left(I,:\right)\Lambda U^{\prime }\left(I,:\right)$. Then we have $\Theta \left(I,I\right)=\sqrt{\mathrm{diag}(U\left(I,:\right)\Lambda U^{\prime }\left(I,:\right))}$ when Condition (II1) holds such that $\tilde{P}$ has unit-diagonals. Set $J_{*}=N_{U}\left(I,I\right)\Theta \left(I,I\right)\equiv \sqrt{\mathrm{diag}(U_{*}\left(I,:\right)\Lambda U_{*}^{\prime }\left(I,:\right))},Z_{*}=N_{U}^{-1}N_{M}\Pi ,Y_{*}=UU_{*}^{-1}\left(I,:\right)$. By Equation (6), we have

(7) $$\begin{array}{c} Z_{*}=Y_{*}J_{*}\equiv UU_{*}^{-1}\left(I,:\right)\mathrm{diag}\left(U_{*}\left(I,:\right)\Lambda U_{*}^{\prime }\left(I,:\right)\right). \end{array}$$

Meanwhile, since $N_{U}^{-1}N_{M}$ is an n×n positive diagonal matrix, we have

(8) $$\begin{array}{c} \Pi \left(i,:\right)=\frac{Z_{*}\left(i,:\right)}{∥Z_{*}{\left(i,:\right)∥}_{1}},i\in \left[n\right]. \end{array}$$

With given Ω and *K*, we can obtain $U,U_{*}$ and Λ. The above analysis shows that once $U_{*}\left(I,:\right)$ is known, we can exactly recover Π by Equations (7) and (8). From Lemma 3, we know that $U_{*}=YU_{*}\left(I,:\right)$ forms the IC structure. Ref. [43] proposes the SVM-cone algorithm (i.e., Algorithm A2) which can exactly obtain $U_{*}\left(I,:\right)$ from the ideal cone $U_{*}=YU_{*}\left(I,:\right)$ with inputs $U_{*}$ and *K*.

Based on the above analysis, we are now ready to give the ideal SVM-cone-DCMMSB algorithm. Input Ω,K. Output: Π.

- Let $\Omega =U\Lambda U^{\prime }$ be the top-*K* eigen-decomposition of Ω such that $U\in R^{n\times K},\Lambda \in R^{K\times K},U^{\prime }U=I$. Let $U_{*}=N_{U}U$, where $N_{U}$ is a n×n diagonal matrix whose *i*-th diagonal entry is $\frac{1}{{∥U\left(i,:\right)∥}_{F}}$ for i∈[n].
- Run SVM-cone algorithm on $U_{*}$ assuming that there are *K* communities to obtain I.
- Set $J_{*}=\sqrt{\mathrm{diag}(U_{*}\left(I,:\right)\Lambda U_{*}^{\prime }\left(I,:\right))},Y_{*}=UU_{*}^{-1}\left(I,:\right),Z_{*}=Y_{*}J_{*}$.
- Recover Π by setting $\Pi \left(i,:\right)=\frac{Z_{*}\left(i,:\right)}{∥Z_{*}{\left(i,:\right)∥}_{1}}$ for i∈[n].

With given $U_{*}$ and *K*, since the SVM-cone algorithm returns $U_{*}\left(I,:\right)$, the ideal SVM-cone-DCMMSB exactly (for detail, see Appendix B) returns Π.

Now, we review the SVM-cone-DCMMSB algorithm of [43], where this algorithm can be seen as an extension of SPACL designed under MMSB to fit DCMM. For the real case, use ${\hat{Y}}_{*},{\hat{J}}_{*},{\hat{Z}}_{*},{\hat{\Pi }}_{*}$ given in Algorithm 2 to estimate $Y_{*},J_{*},Z_{*},\Pi$, respectively.
**Algorithm 2** SVM-cone-DCMMSB [43]**Require:** The adjacency matrix $A\in R^{n\times n}$ and the number of communities *K*.**Ensure:** The estimated n×K membership matrix ${\hat{\Pi }}_{*}$.1: Obtain $\tilde{A}=\hat{U}\hat{\Lambda }{\hat{U}}^{\prime }$, the top *K* eigen-decomposition of *A*. Let ${\hat{U}}_{*}\in R^{n\times K}$ such that ${\hat{U}}_{*}\left(i,:\right)=\frac{\hat{U}\left(i,:\right)}{∥\hat{U}{\left(i,:\right)∥}_{F}}$ for i∈[n].2: Apply SVM-cone algorithm (i.e., Algorithm A2) on the rows of ${\hat{U}}_{*}$ assuming there are *K* communities to obtain ${\hat{I}}_{*}$, the index set returned by SVM-cone algorithm.3: Set ${\hat{J}}_{*}=\sqrt{\mathrm{diag}({\hat{U}}_{*}\left({\hat{I}}_{*},:\right)\hat{\Lambda }{\hat{U}}_{*}^{\prime }\left({\hat{I}}_{*},:\right))},{\hat{Y}}_{*}=\hat{U}{\hat{U}}_{*}^{-1}\left({\hat{I}}_{*},:\right),{\hat{Z}}_{*}={\hat{Y}}_{*}{\hat{J}}_{*}$ . Then set ${\hat{Z}}_{*}=\max\left(0,{\hat{Z}}_{*}\right)$.4: Estimate Π(i,:) by ${\hat{\Pi }}_{*}\left(i,:\right)={\hat{Z}}_{*}\left(i,:\right)/{∥{\hat{Z}}_{*}\left(i,:\right)∥}_{1},i\in \left[n\right]$.

### Consistency under DCMM

Assume that

(A2) ${\tilde{P}}_{\max}\theta_{\max}{∥\theta ∥}_{1}\geq \log\left(n\right)$.

Since we let ${\tilde{P}}_{\max}\leq C$, Assumption (A2) equals $\theta_{\max}{∥\theta ∥}_{1}\geq \log\left(n\right)/C$. The following lemma bounds ∥A−Ω∥ under $DCMM_{n}\left(K,\tilde{P},\Pi ,\Theta \right)$ when Assumption (A2) holds.

**Lemma** **4.** *Under $DCMM_{n}\left(K,\tilde{P},\Pi ,\Theta \right)$, when Assumption (A2) holds, with probability at least $1-o(n^{-\alpha })$, we have*

$$\begin{array}{c} ∥A-\Omega ∥\leq \frac{\alpha +1+\sqrt{\left(\alpha +1)(\alpha +19\right)}}{3}\sqrt{{\tilde{P}}_{\max}\theta_{\max}{∥\theta ∥}_{1}\log\left(n\right)}. \end{array}$$

**Remark** **9.** *Consider a special case when $\Theta =\sqrt{\rho }I$ such that DCMM degenerates to MMSB, since ${\tilde{P}}_{\max}$ is assumed to be 1 under MMSB, Assumption (A2) and the upper bound of ∥A−Ω∥ in Lemma 4 are consistent with that of Lemma 1. When all nodes are pure, DCMM degenerates to DCSBM [45], then the upper bound of ∥A−Ω∥ in Lemma 4 is also consistent with Lemma 2.2 of [50]. Meanwhile, this bound is also consistent with Equation (6.34) in the first version of [41], which also applies the Bernstein inequality to bound ∥A−Ω∥. However, the bound is $C\sqrt{\theta_{\max}{∥\theta ∥}_{1}}$ in Equation (C.53) of the latest version for [41], which applies Corollary 3.12 and Remark 3.13 of [76] to obtain the bound. Though the bound in Equation (C.53) of the latest version for [41] is sharper by a $\sqrt{\log(n)}$ term, Corollary 3.12 of [76] has constraints on W(i,j) (here, W=A−Ω) such that W(i,j) can be written as $W\left(i,j\right)=\xi_{ij}b_{ij}$, where $\left\{\xi_{i,j}:i\geq j\right\}$ are independent symmetric random variables with unit variance, and $\left\{b_{i,j}:i\geq j\right\}$ are given scalars; see the proof of Corollary 3.12 [76] for detail. Therefore, without causing confusion, we also use $A_{\mathrm{re}}$ to denote the constraint A used in [41] such that $∥A_{\mathrm{re}}-\Omega ∥\leq C\sqrt{\theta_{\max}{∥\theta ∥}_{1}}$. Furthermore, if we set $\rho \geq \max_{i,j}\Omega \left(i,j\right)$ such that $\rho \geq \theta_{\max}^{2}$, the bound in Lemma 4 also equals $∥A-\Omega ∥\leq C\sqrt{\rho n\log(n)}$ and the assumption (A2) reads ${\tilde{P}}_{\max}\rho n\geq \log\left(n\right)$. The bound $∥A_{\mathrm{re}}-\Omega ∥\leq C\sqrt{\theta_{\max}{∥\theta ∥}_{1}}$ in Equation (C.53) of [41] reads $∥A_{\mathrm{re}}-\Omega \left|\right|\leq C\sqrt{\rho n}$.*

**Lemma** **5.** *(Row-wise eigenspace error) Under $DCMM_{n}\left(K,\tilde{P},\Pi ,\Theta \right)$, when Assumption (A2) holds, suppose $\sigma_{K}\left(\Omega \right)\geq C\theta_{\max}\sqrt{{\tilde{P}}_{\max}n\log\left(n\right)}$, with probability at least $1-o(n^{-\alpha })$.*

- *When we apply Theorem 4.2 of [64], we have*
$$\begin{array}{c} ∥\hat{U}{\hat{U}}^{\prime }-UU^{\prime }{∥}_{2\to \infty }=O\left(\frac{\theta_{\max}\sqrt{{\tilde{P}}_{\max}K}\left(\frac{\theta_{\max}\kappa \left(\Omega \right)}{\theta_{\min}}\sqrt{\frac{n}{K\lambda_{K}\left(\Pi^{\prime }\Pi \right)}}+\sqrt{\log(n)}\right)}{\theta_{\min}^{2}\sigma_{K}\left(\tilde{P}\right)\lambda_{K}\left(\Pi^{\prime }\Pi \right)}\right). \end{array}$$
- *When we apply Theorem 4.2 of [65], we have*
$$\begin{array}{c} ∥\hat{U}{\hat{U}}^{\prime }-UU^{\prime }{∥}_{2\to \infty }=O\left(\frac{\theta_{\max}\sqrt{{\tilde{P}}_{\max}\theta_{\max}{∥\theta ∥}_{1}\log\left(n\right)}}{\theta_{\min}^{3}\sigma_{K}\left(\tilde{P}\right)\lambda_{K}^{1.5}\left(\Pi^{\prime }\Pi \right)}\right). \end{array}$$

Without causing confusion, we also use $\varpi ,\varpi_{1},\varpi_{2}$ under DCMM as Lemma 2 for notation convenience.

**Remark** **10.** *When $\Theta =\sqrt{\rho }I$ such that DCMM degenerates to MMSB, bounds in Lemma 5 are consistent with those of Lemma 2.*

**Remark** **11** (Comparison to Theorem I.3 [43]). *Note that the ρ in [43] is $\theta_{\max}^{2}$, which gives that the row-wise eigenspace concentration in Theorem I.3 [43] is $O(\frac{\theta_{\max}\sqrt{Kn}{∥U∥}_{2\to \infty }\log^{\xi }\left(n\right)}{\sigma_{K}\left(\Omega \right)})$ when using $∥A_{\mathrm{re}}-\Omega ∥\leq C\sqrt{\rho n}$ and this value is at least $O(\frac{\sqrt{\theta_{\max}{∥\theta ∥}_{1}K}{∥U∥}_{2\to \infty }\log^{\xi }\left(n\right)}{\sigma_{K}\left(\Omega \right)})$. Since ${∥U∥}_{2\to \infty }\leq \frac{\theta_{\max}}{\theta_{\min}\sqrt{\lambda_{K}\left(\Pi^{\prime }\Pi \right)}}$ by Lemma II.1 of [43] and $\sigma_{K}\left(\Omega \right)\geq \theta_{\min}^{2}\sigma_{K}\left(\tilde{P}\right)\lambda_{K}\left(\Pi^{\prime }\Pi \right)$ by the proof of Lemma 5, we see that the upper bound of Theorem I.3 [43] is $O(\frac{\theta_{\max}\sqrt{K\theta_{\max}{∥\theta ∥}_{1}}\log^{\xi }\left(n\right)}{\theta_{\min}^{3}\sigma_{K}\left(\tilde{P}\right)\lambda_{K}^{1.5}\left(\Pi^{\prime }\Pi \right)})$, which is $\sqrt{K}\log^{\xi -0.5}\left(n\right)$ (recall that ξ>1) times than our $\varpi_{2}$. Again, Theorem I.3 [43] has stronger requirements on the sparsity of $\theta_{\max}{∥\theta ∥}_{1}$ and the lower bound of $\sigma_{K}\left(\Omega \right)$ than our Lemma 5. When using the bound of ∥A−Ω∥ in our Lemma 4 to obtain the row-wise eigenspace concentration in Theorem I.3 [43], their upper bound is $\sqrt{K}\log^{\xi }\left(n\right)$ times than our $\varpi_{2}$. Similar to the first bullet given after Lemma 2, whether using $∥A-\Omega ∥\leq C\sqrt{\theta_{\max}{∥\theta ∥}_{1}\log\left(n\right)}$ or $∥A_{\mathrm{re}}-\Omega ∥\leq C\sqrt{\theta_{\max}{∥\theta ∥}_{1}}$ does not change our ϖ under DCMM.*

**Remark** **12** (Comparison to Lemma 2.1 [41]). *The fourth bullet of Lemma 2.1 [41] is the row-wise deviation bound for the eigenvectors of the adjacency matrix under some assumptions translated to our $\kappa \left(\Pi^{\prime }\Pi \right)=O\left(1\right)$, Assumption (A2) and lower bound requirement on $\sigma_{K}\left(\Omega \right)$ since they apply Lemma C.2 [41]. The row-wise deviation bound in the fourth bullet of Lemma 2.1 [41] reads $O(\frac{\theta_{\max}K^{1.5}\sqrt{\theta_{\max}{∥\theta ∥}_{1}\log\left(n\right)}}{\sigma_{K}\left(\tilde{P}\right){∥\theta ∥}_{F}^{3}})$, where the denominator is $\sigma_{K}\left(\tilde{P}\right){∥\theta ∥}_{F}^{3}$ instead of our $\theta_{\min}^{3}\sigma_{K}\left(\tilde{P}\right)\lambda_{K}^{1.5}\left(\Pi^{\prime }\Pi \right)$ due to the fact that [41] uses $\frac{\sigma_{K}\left(\tilde{P}\right){∥\theta ∥}_{F}^{2}}{K}$ to roughly estimate $\sigma_{K}\left(\Omega \right)$ while we apply $\theta_{\min}^{2}\sigma_{K}\left(\tilde{P}\right)\lambda_{K}\left(\Pi^{\prime }\Pi \right)$ to strictly control the lower bound of $\sigma_{K}\left(\Omega \right)$. Therefore, we see that the row-wise deviation bound in the fourth bullet of Lemma 2.1 [41] is consistent with our bounds in Lemma 5 when $\kappa \left(\Pi^{\prime }\Pi \right)=O\left(1\right)$, while our row-wise eigenspace errors in Lemma 5 are more applicable than those of [41] since we do not need to add a constraint on $\Pi^{\prime }\Pi$ such that $\kappa \left(\Pi^{\prime }\Pi \right)=O\left(1\right)$. The upper bound of ∥A−Ω∥ of [41] is $C\sqrt{\theta_{\max}{∥\theta ∥}_{1}}$ given in their Equation (C.53) under $DCMM_{n}\left(K,\tilde{P},\Pi ,\Theta \right)$, while ours is $C\sqrt{\theta_{\max}{∥\theta ∥}_{1}\log\left(n\right)}$ in Lemma 4, since our bound of the row-wise eigenspace error in Lemma 5 is consistent with the fourth bullet of Lemma 2.1 [41], this supports the statement that the row-wise eigenspace error does not rely on ∥A−Ω∥ given in the first bullet after Lemma 2.*

Let $\pi_{\min}=\min_{1\leq k\leq K}1^{\prime }\Pi e_{k}$ , where $\pi_{\min}$ measures the minimum summation of nodes belonging to a certain community. Increasing $\pi_{\min}$ makes the network tend to be more balanced, and vice versa. Meanwhile, the term $\pi_{\min}$ appears when we propose a lower bound of η defined in Lemma A1 to keep track of the model parameters in our main theorem under $DCMM_{n}\left(K,\tilde{P},\Pi ,\Theta \right)$. The next theorem gives the theoretical bounds on estimations of memberships under DCMM.

**Theorem** **2.** Under $DCMM_{n}\left(K,\tilde{P},\Pi ,\Theta \right)$, let $\hat{\Pi }$ be obtained from Algorithm 2, suppose conditions in Lemma 5 hold, and there exists a permutation matrix $P_{*}\in R^{K\times K}$ such that with probability at least $1-o(n^{-\alpha })$, we have

$$\begin{array}{c} \max_{i\in [n]}{∥e_{i}^{\prime }\left({\hat{\Pi }}_{*}-\Pi P_{*}\right)∥}_{1}=O\left(\frac{\theta_{\max}^{15}K^{5}\varpi \kappa^{4.5}\left(\Pi^{\prime }\Pi \right)\lambda_{1}^{1.5}\left(\Pi^{\prime }\Pi \right)}{\theta_{\min}^{15}\pi_{\min}}\right). \end{array}$$

For comparison, Table 4 summarizes the necessary conditions and dependence on model parameters of rates for Theorem 2 and Theorem 3.2 [43], where the dependence on *K* and log(n) are analyzed in Remark 13 given below.

**Remark** **13.** *(Comparison to Theorem 3.2 [43]) Our bound in Theorem 2 is written as combinations of model parameters and *Π* can follow any distribution as long as Condition (II2) holds, where such model parameters’ related form of estimation bound is convenient for further theoretical analysis (see Corollary 2), while the bound in Theorem 3.2 [43] is built when *Π* follows a Dirichlet distribution and $\kappa \left(\Pi^{\prime }\Theta^{2}\Pi \right)=O\left(1\right)$. Meanwhile, since Theorem 3.2 [43] applies Theorem I.3 [43] to obtain the row-wise eigenspace error, the bound in Theorem 3.2 [43] should multiply $\log^{\xi }\left(n\right)$ by Remark 11, and this is also supported by the fact that in the proof of Theorem 3.1 [43], when computing bound of $\epsilon_{0}$ (in the language in Ref. [43]) [43] ignores the $\log^{\xi }\left(n\right)$ term.*

*Consider a special case by setting $\lambda_{K}\left(\Pi^{\prime }\Pi \right)=O\left(\frac{n}{K}\right),\pi_{\min}=O\left(\frac{n}{K}\right)$ and $\frac{\theta_{\max}}{\theta_{\min}}=O\left(1\right)$ with $\theta_{\max}=\sqrt{\rho }$, where such case matches the setting $\kappa \left(\Pi^{\prime }\Theta^{2}\Pi \right)=O\left(1\right)$ in Theorem 3.2 [43]. Now we focus on analyzing the powers of K in our Theorem 2 and Theorem 3.2 [43]. Under this case, the power of K in the estimation bound of Theorem 2 is 6 by basic algebra; since $\min\left(K^{2},\kappa^{2}\left(\Omega \right)\right)=\min\left(K^{2},O\left(1\right)\right)=O\left(1\right),\frac{1}{\lambda_{K}^{2}\left(\Pi^{\prime }\Theta^{2}\Pi \right)}=O\left(\frac{K^{2}}{\rho^{2}n^{2}}\right)$, $\frac{1}{\eta }=O\left(K\right)$ by Lemma A1 where η in Lemma A1 follows the same definition as that of Theorem 3.2 [43], and the bound in Theorem 3.2 [43] should multiply $\sqrt{K}$ because (in the language of Ref. [43]) $∥{\left({\hat{Y}}_{C}{\hat{Y}}_{C}^{\prime }\right)}^{-1}{∥}_{F}$ should be no larger than $\frac{\sqrt{K}}{\lambda_{K}\left({\hat{Y}}_{C}{\hat{Y}}_{C}^{\prime }\right)}$ instead of $\frac{1}{\lambda_{K}\left({\hat{Y}}_{C}{\hat{Y}}_{C}^{\prime }\right)}$ in the proof of Theorem 2.8 [43], the power of K is 6 by checking the bound of Theorem 3.2 [43]. Meanwhile, note that our bound in Theorem 2 is $l_{1}$ bound, while the bound in Theorem 3.2 [43] is $l_{2}$ bound, and when we translate the $l_{2}$ bound of Theorem 3.2 [43] into $l_{1}$ bound, the power of K is 6.5 for Theorem 3.2 [43], suggesting that our bound in Theorem 2 has less dependence on K than that of Theorem 3.2 [43].*

The following corollary is obtained by adding some conditions on the model parameters.

**Corollary** **2.** *Under $DCMM_{n}\left(K,\tilde{P},\Pi ,\Theta \right)$, when conditions of Lemma 5 hold, suppose $\lambda_{K}\left(\Pi^{\prime }\Pi \right)=O\left(\frac{n}{K}\right),\pi_{\min}=O\left(\frac{n}{K}\right)$ and K=O(1), with probability at least $1-o(n^{-\alpha })$, we have*

$$\begin{array}{c} \max_{i\in [n]}{∥e_{i}^{\prime }\left({\hat{\Pi }}_{*}-\Pi P_{*}\right)∥}_{1}=O\left(\frac{\theta_{\max}^{16}\sqrt{{\tilde{P}}_{\max}\theta_{\max}{∥\theta ∥}_{1}\log\left(n\right)}}{\theta_{\min}^{18}\sigma_{K}\left(\tilde{P}\right)n}\right). \end{array}$$

*Meanwhile, when $\theta_{\max}=O\left(\sqrt{\rho }\right),\theta_{\min}=O\left(\sqrt{\rho }\right)$ (i.e., $\frac{\theta_{\min}}{\theta_{\max}}=O\left(1\right)$), we have*

$$\begin{array}{c} \max_{i\in [n]}{∥e_{i}^{\prime }\left({\hat{\Pi }}_{*}-\Pi P_{*}\right)∥}_{1}=O\left(\frac{1}{\sigma_{K}\left(\tilde{P}\right)}\sqrt{\frac{{\tilde{P}}_{\max}\log\left(n\right)}{\rho n}}\right). \end{array}$$

**Remark** **14.** *When $\lambda_{K}\left(\Pi^{\prime }\Pi \right)=O\left(\frac{n}{K}\right),K=O\left(1\right),\theta_{\max}=O\left(\sqrt{\rho }\right)$ and $\theta_{\min}=O\left(\sqrt{\rho }\right)$, the requirement $\sigma_{K}\left(\Omega \right)\geq C\theta_{\max}\sqrt{{\tilde{P}}_{\max}n\log\left(n\right)}$ in Lemma 5 holds naturally. By the proof of Lemma 5, $\sigma_{K}\left(\Omega \right)$ has a lower bound $\theta_{\min}^{2}\sigma_{K}\left(\tilde{P}\right)\lambda_{K}\left(\Pi^{\prime }\Pi \right)=O\left(\theta_{\min}^{2}\sigma_{K}\left(P\right)n\right)$. To make the requirement $\sigma_{K}\left(\Omega \right)\geq C\theta_{\max}\sqrt{{\tilde{P}}_{\max}n\log\left(n\right)}$ always hold, we just need $\theta_{\min}^{2}\sigma_{K}\left(\tilde{P}\right)n\geq C\theta_{\max}\sqrt{{\tilde{P}}_{\max}n\log\left(n\right)}$, and it gives $\sigma_{K}\left(\tilde{P}\right)\geq C\sqrt{\frac{{\tilde{P}}_{\max}\log\left(n\right)}{\rho n}}$, which matches the requirement of consistent estimation in Corollary 2.*

Using SCSTC to Corollary 2, let $\Theta =\sqrt{\rho }I$ such that DCMM degenerates to MMSB, and it is easy to see that the bound in Lemma 2 is consistent with that of Lemma 1. Therefore, the separation condition, alternative separation condition and sharp threshold obtained from Corollary 2 for the extended version of SPACL under DCMM are consistent with classical results, as shown in Table 1 and Table 2 (detailed analysis will be provided in next paragraph). Meanwhile, when $\theta_{\max}=O\left(\sqrt{\rho }\right),\theta_{\min}=O\left(\sqrt{\rho }\right)$ and settings in Corollary 2 hold, the bound in Theorem 2.2 [41] is of order $\frac{1}{\sigma_{K}\left(\tilde{P}\right)}\sqrt{\frac{\log(n)}{\rho n}}$, which is consistent with our bound in Corollary 2.

Consider a mixed membership network under the settings of Corollary 2 when $\Theta =\sqrt{\rho }I$ such that DCMM degenerates to MMSB. By Corollary 2, $\frac{\sigma_{K}\left(\tilde{P}\right)}{\sqrt{{\tilde{P}}_{\max}}}$ should shrink slower than $\sqrt{\frac{\log(n)}{\rho n}}$. We further assume that $\tilde{P}=\left(2-\beta \right)I_{K}+\left(\beta -1\right)11^{\prime }$ for β∈[1,2)∪(2,∞); we see that this $\tilde{P}$ with unit diagonals and β−1 as non-diagonal entries still satisfies Condition (II1). Meanwhile, $\sigma_{K}\left(\tilde{P}\right)=\left|\beta -2\right|={\tilde{P}}_{\max}-{\tilde{P}}_{\min}$ and ${\tilde{P}}_{\max}=\max\left(1,\beta -1\right)$, so $\frac{\sigma_{K}\left(\tilde{P}\right)}{\sqrt{{\tilde{P}}_{\max}}}=\frac{\left|\beta -2\right|}{\sqrt{\max(1,\beta -1)}}$ should shrink slower than $\sqrt{\frac{\log(n)}{\rho n}}$. Setting P=ρP as the probability matrix for such $\tilde{P}$, we have $p_{\mathrm{out}}=\rho \left(\beta -1\right),p_{\mathrm{in}}=\rho$, and $\max\left(p_{\mathrm{in}},p_{\mathrm{out}}\right)=\rho \max\left(1,\beta -1\right)$. Sure, the separation condition $\frac{|p_{\mathrm{in}}-p_{\mathrm{out}}|}{\sqrt{\max(p_{\mathrm{in}},p_{\mathrm{out}})}}\equiv \frac{\sqrt{\rho }\left|\beta -2\right|}{\sqrt{\max(1,\beta -1)}}$ should shrink slower than $\sqrt{\frac{\log(n)}{n}}$, which satisfies Equation (1). For an alternative separation condition and sharp threshold, just follow a similar analysis as that of MMSB, and we obtain the results in Table 1 and Table 2.

## 6. Numerical Results

In this section, we present the experimental results for an overlapping network by plotting the phase transition behaviors for both SPACL and SVM-cone-DCMMSB to show that the two methods achieve the threshold in Equation (2) under $MMSB(n,K,\Pi ,p_{\mathrm{in}},p_{\mathrm{out}})$ when K=2 and K=3. We also use some experiments to show that the spectral methods studied in [26,30,48,50] achieve the threshold in Equation (2) under $SBM(n,K,p_{\mathrm{in}},p_{\mathrm{out}})$ when K=2 and K=3 for the non-overlapping network. To measure the performance of different algorithms, we use the error rate defined below:

(9) $$\begin{array}{c} \min_{P\in \{K\times K\;\mathrm{permutation}\;\mathrm{matrix}\}}\frac{1}{n}{∥\hat{\Pi }-\Pi P∥}_{1}. \end{array}$$

For all simulations, let $p_{\mathrm{in}}=\alpha_{\mathrm{in}}\frac{\log(n)}{n}$ and $p_{\mathrm{out}}=\alpha_{\mathrm{out}}\frac{\log(n)}{n}$ be diagonal and non-diagonal entries of *P*, respectively. Since *P* is the probability matrix, $\alpha_{\mathrm{in}}$ and $\alpha_{\mathrm{out}}$ should be located in $\left(0,\frac{n}{\log(n)}\right]$. After setting *P* and Π, each simulation experiment has the following steps:

(a) Set $\Omega =\Pi P\Pi^{\prime }$.
(b) Let *W* be an n×n symmetric matrix such that all diagonal entries of *W* are 0, and W(i,j) are independent centered Bernoulli with parameters Ω(i,j). Let A=Ω−diag(Ω)+W be the adjacency matrix of a simulated network with mixed memberships under MMSB (so there are no loops).
(c) Apply spectral clustering method to *A* with *K* communities. Record the error rate.
(d) Repeat (b)–(c) 50 times, and report the mean of the error rates over the 50 times.

**Figure 1:**
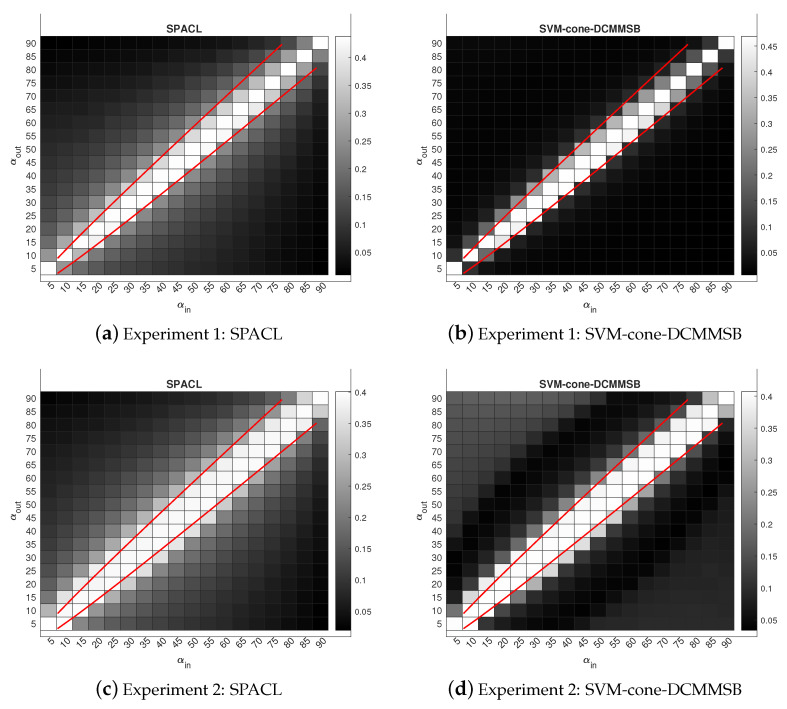
Phase transition for SPACL and SVM-cone-DCMMSB under MMSB: darker pixels represent lower error rates. The red lines represent $\frac{|\alpha_{\mathrm{in}}-\alpha_{\mathrm{out}}|}{\sqrt{\max(\alpha_{\mathrm{in}},\alpha_{\mathrm{out}})}}=1$.

Experiment 1: Set n=600,K=2, and $n_{0}=250$, where $n_{0}$ is the number of pure nodes in each community. Let all mixed nodes have mixed membership (1/2,1/2). Since $\alpha_{\mathrm{in}}$ and $\alpha_{\mathrm{out}}$ should be set to less than $\frac{n}{\log(n)}=\frac{600}{\log(600)}\approx 93.795$, we let $\alpha_{\mathrm{in}}$ and $\alpha_{\mathrm{out}}$ be in the range of {5,10,15,…,90}. For each pair of $\left(\alpha_{\mathrm{in}},\alpha_{\mathrm{out}}\right)$, we generate *P* and then run steps (a)–(d) for this experiment. So, this experiment generates an adjacency matrix of network with mixed memberships under $MMSB(n,2,\Pi ,p_{\mathrm{in}},p_{\mathrm{out}})$. The numerical results are displayed in panels (a) and (b) of Figure 1. We can see that our theoretical bounds (red lines) are quite tight, and the threshold regions obtained from the boundaries of light white areas in panels (a) and (b) are close to our theoretical bounds. Meanwhile, both methods perform better when $\frac{|\alpha_{\mathrm{in}}-\alpha_{\mathrm{out}}|}{\sqrt{\max(\alpha_{\mathrm{in}},\alpha_{\mathrm{out}})}}$ increases and SVM-cone-DCMMSB outperforms SPACL for this experiment since panel (b) is darker than panel (a). Note that the network generated here is an assortative network when $\alpha_{\mathrm{in}}>\alpha_{\mathrm{out}}$, and the network is a dis-assortative network when $\alpha_{\mathrm{in}}<\alpha_{\mathrm{out}}$. So, the results of this experiment support our finding that SPACL and SVM-cone-DCMMSB achieves the threshold in Equation (2) for both assortative and dis-assortative networks.

Experiment 2: Set n=600,K=3, and $n_{0}=150$. Let all mixed nodes have mixed membership (1/3,1/3,1/3). Let $\alpha_{\mathrm{in}}$ and $\alpha_{\mathrm{out}}$ range in {5,10,15,…,90}. Sure, this experiment is under $MMSB(n,3,\Pi ,p_{\mathrm{in}},p_{\mathrm{out}})$. The numerical results are displayed in panels (c) and (d) of Figure 1. We see that both methods perform poorly in the region between the two red lines, and the analysis of the numerical results for this experiment is similar to that of Experiment 1.

**Figure 2:**
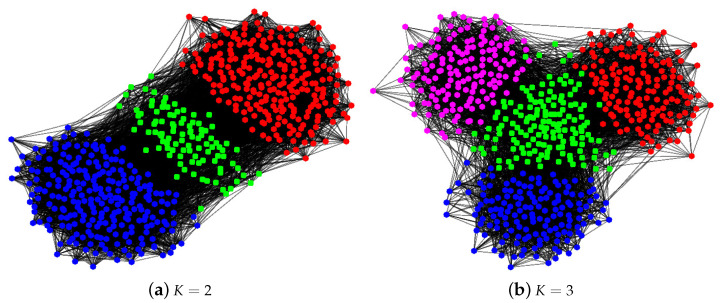
Panel (**a**): a graph generated from the mixed membership stochastic blockmodel with n=600 nodes and 2 communities. Among the 600 nodes, each community has 250 pure nodes. For the 100 mixed nodes, they have mixed membership (1/2,1/2). Panel (**b**): a graph generated from MMSB with n=600 nodes and 3 communities. Among the 600 nodes, each community has 150 pure nodes. For the 150 mixed nodes, they have mixed membership (1/3,1/3,1/3). Nodes in panels (**a**,**b**) connect with probability $p_{\mathrm{in}}=60/600$ and $p_{\mathrm{out}}=1/600$, so the two networks in both panels are assortative networks. For panel (**a**), error rates for SPACL and SVM-cone-DCMMSB are 0.0285 and 0.0175, respectively, where error rate is defined in Equation (9). For panel (**b**), error rates for SPACL and SVM-cone-DCMMSB are 0.0709 and 0.0436, respectively. For both panels, dots in the same color are pure nodes in the same community and green square nodes are mixed.

**Figure A1:**
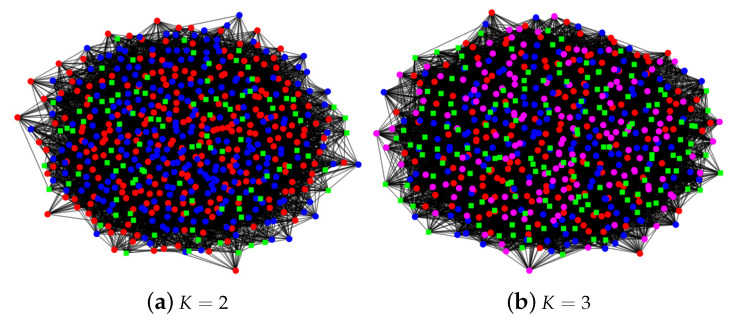
Panel (**a**): a graph generated from MMSB with n=600 and K=2. Each community has 250 pure nodes. For the 100 mixed nodes, they have mixed membership (1/2,1/2). Panel (**b**): a graph generated from MMSB with n=600 and K=3. Each community has 150 pure nodes. For the 150 mixed nodes, they have mixed membership (1/3,1/3,1/3). Nodes in panels (**a**,**b**) connect with probability $p_{\mathrm{in}}=1/600$ and $p_{\mathrm{out}}=60/600$, so the two networks in both panels are dis-assortative networks. For panel (**a**), error rates for SPACL and SVM-cone-DCMMSB are 0.0298 and 0.0180, respectively. For panel (**b**), error rates for SPACL and SVM-cone-DCMMSB are 0.1286 and 0.0896, respectively. For both panels, dots in the same color are pure nodes in the same community, and green square nodes are mixed.

For visualization, we plot two networks generated from $MMSB(n,K,\Pi ,p_{\mathrm{in}},p_{\mathrm{out}})$ when K=2 and K=3 in Figure 2. We also plot two dis-assortative networks generated from $MMSB(n,K,\Pi ,p_{\mathrm{in}},p_{\mathrm{out}})$ when K=2 and K=3 in Figure A1 in Appendix A. In Experiments 1 and 2, there exist some mixed nodes for network generated under MMSB. The following two experiments only focus on network under SBM such that all nodes are pure. Meanwhile, we only consider four spectral algorithms studied in [26,30,48,50] for the non-overlapping network. For convenience, we call the spectral clustering method studied in [26] normalized principle component analysis (nPCA), and Algorithm 1 studied in [30] ordinary principle component analysis (oPCA), where nPCA and oPCA are also considered in [50]. Next, we briefly review nPCA, oPCA, RSC and SCORE.

The nPCA algorithm is as follows with input A,K and output $\hat{\Pi }$.

- Obtain the graph Laplacian $L=D^{-1/2}AD^{-1/2}$, where *D* is a diagonal matrix with $D\left(i,i\right)=\sum_{j=1}^{n}A\left(i,j\right)$ for i∈[n].
- Obtain $\hat{U}\hat{\Lambda }{\hat{U}}^{\prime }$, the top *K* eigen-decomposition of *L*.
- Apply k-means algorithm to $\hat{U}$ to obtain $\hat{\Pi }$.

The oPCA algorithm is as follows with input A,K and output $\hat{\Pi }$.

- Obtain $\hat{U}\hat{\Lambda }{\hat{U}}^{\prime }$, the top *K* eigen-decomposition of *A*.
- Apply k-means algorithm to $\hat{U}$ to obtain $\hat{\Pi }$.

The RSC algorithm is as follows with input A,K, regularizer τ, and output $\hat{\Pi }$.

- Obtain the regularized graph Laplacian $L_{\tau }=D_{\tau }^{-1/2}AD_{\tau }^{-1/2}$, where $D_{\tau }=D+\tau I$, and the default τ is the average node degree.
- Obtain $\hat{U}\hat{\Lambda }{\hat{U}}^{\prime }$, the top *K* eigen-decomposition of $L_{\tau }$. Let ${\hat{U}}_{*}$ be the row-normalized version of $\hat{U}$.
- Apply k-means algorithm to ${\hat{U}}_{*}$ to obtain $\hat{\Pi }$.

The SCORE algorithm is as follows with input A,K, threshold $T_{n}$ and output $\hat{\Pi }$.

- Obtain the *K* (unit-norm) leading eigenvectors of *A*: ${\hat{\eta }}_{1},{\hat{\eta }}_{2},\ldots ,{\hat{\eta }}_{K}$.
- Obtain an n×(K−1) matrix ${\hat{R}}_{*}$ such that for i∈[n],k∈[K−1],
  $${\hat{R}}_{*}\left(i,k\right)=\left\{\begin{array}{cc} \hat{R}\left(i,k\right), & \mathrm{if}\;|\hat{R}\left(i,k\right)|\leq T_{n},\\ T_{n}, & \mathrm{if}\;\hat{R}\left(i,k\right)>T_{n},\\ -T_{n}, & \mathrm{if}\;\hat{R}\left(i,k\right)<-T_{n}, \end{array}\right.$$
  where $\hat{R}\left(i,k\right)=\frac{{\hat{\eta }}_{k+1}\left(i\right)}{{\hat{\eta }}_{1}}\left(i\right)$, and the default $T_{n}$ is log(n).
- Apply k-means algorithm to ${\hat{R}}_{*}$ to obtain $\hat{\Pi }$.

We now describe Experiments 3 and 4 under $SBM(n,K,p_{\mathrm{in}},p_{\mathrm{out}})$ when K=2 and K=3.

**Figure 3:**
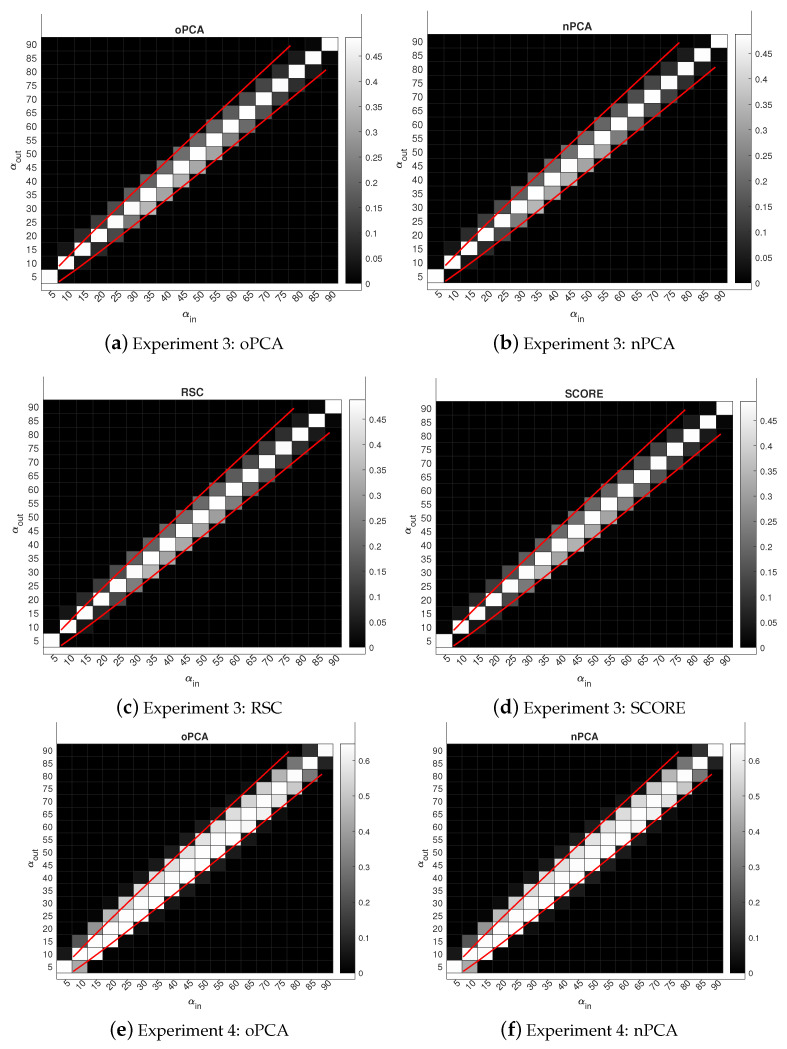

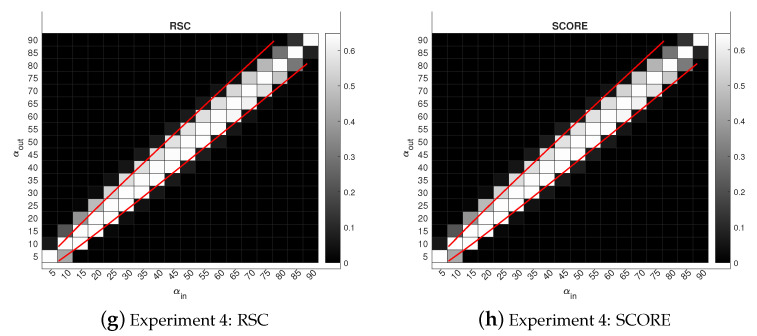
Phase transition for oPCA, nPCA, RSC and SCORE under SBM: darker pixels represent lower error rates. The red lines represent $\frac{|\alpha_{\mathrm{in}}-\alpha_{\mathrm{out}}|}{\sqrt{\max(\alpha_{\mathrm{in}},\alpha_{\mathrm{out}})}}=1$.

Experiment 3: Set n=600,K=2, and $n_{0}=300$, i.e., all nodes are pure and each community has 300 nodes. So this experiment generates the adjacency matrix of the network under $SBM(n,2,p_{\mathrm{in}},p_{\mathrm{out}})$. Numerical results are displayed in panels (a)–(d) of Figure 3. We can see that these spectral clustering methods achieve the threshold in Equation (2).

Experiment 4: Set n=600,K=3, and $n_{0}=200$, i.e., all nodes are pure and each community has 200 nodes. So, this experiment is under $SBM(n,3,p_{\mathrm{in}},p_{\mathrm{out}})$. The numerical results are displayed in panels (e)–(h) of Figure 3. The results show that these methods achieve threshold in Equation (2).

**Figure 4:**
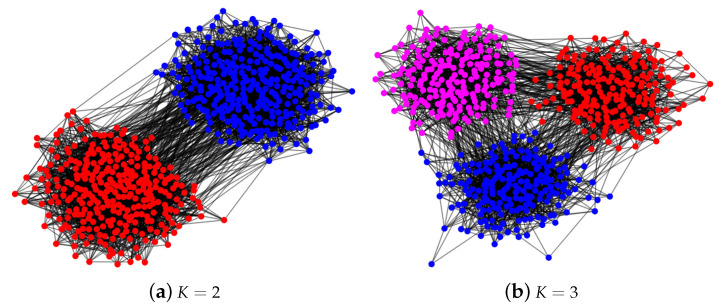
Panel (**a**): a graph generated from SBM(600,2,30/600,2/600). Panel (**b**): a graph generated from SBM(600,3,30/600,2/600). So, in panel (**a**), there are 2 communities and each community has 300 nodes; in panel (**b**), there are 3 communities and each community has 200 nodes. Networks in panels (**a**,**b**) are assortative networks since $p_{\mathrm{in}}>p_{\mathrm{out}}$. For both panels, error rates for oPCA, nPCA, RSC and SCORE are 0. Colors indicate clusters.

**Figure A2:**
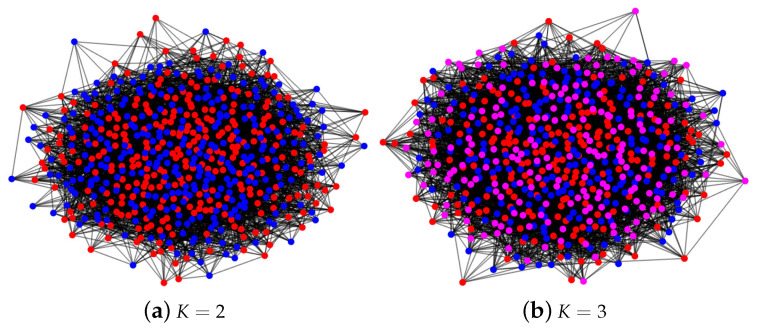
Panel (**a**): a graph generated from SBM(600,2,2/600,30/600). Panel (**b**): a graph generated from SBM(600,3,2/600,30/600). Networks in panels (**a**,**b**) are dis-assortative networks since $p_{\mathrm{in}}<p_{\mathrm{out}}$ . For panel (**a**), error rates for oPCA, nPCA, RSC and SCORE are 0. For panel (**b**), error rates for oPCA, nPCA, RSC and SCORE are 0.0067. Colors indicate clusters.

For visualization, we plot two assortative networks generated from $SBM(n,K,p_{\mathrm{in}},p_{\mathrm{out}})$ when K=2 and K=3 in Figure 4. We also plot two dis-assortative networks generated from $SBM(n,K,p_{\mathrm{in}},p_{\mathrm{out}})$ when K=2 and K=3 in Figure A2 in Appendix A.

## 7. Conclusions

In this paper, the four-step separation condition and sharp threshold criterion SCSTC is summarized as a unified framework to study the consistencies and compare the theoretical error rates of spectral methods under models that can degenerate to SBM in a community detection area. With an application of this criterion, we find some inconsistent phenomena of a few previous works. In particular, using SCSTC, we find that the original theoretical upper bounds on error rates of the SPACL algorithm under MMSB and its extended version under DCMM are sub-optimal for the error rates and requirements on network sparsity. To find how the inconsistent phenomena occur, we re-establish theoretical upper bounds of error rats for both SPACL and its extended version by using recent techniques on row-wise eigenvector deviation. The resulting error bounds explicitly keep track of seven independent model parameters $\left(K,\rho ,\sigma_{K}\left(\tilde{P}\right),\lambda_{K}\left(\Pi^{\prime }\Pi \right),\lambda_{1}\left(\Pi^{\prime }\Pi \right),\theta_{\min},\theta_{\max}\right)$, which allow us to have a further delicate analysis. Compared with the original theoretical results, ours have smaller error rates with lesser dependence on *K* and log(n), weaker requirements on the network sparsity and the lower bound of the smallest nonzero singular value of population adjacency matrix under both MMSB and DCMM. For DCMM, we have no constraint on the distribution of the membership matrix as long as it satisfies the identifiability condition. When considering the separation condition of a standard network and the probability to generate a connected Erdös–Rényi (ER) random graph by using SCSTC, our theoretical results match the classical results. Meanwhile, our theoretical results also match those of Theorem 2.2 [41] under mild conditions, and when DCMM degenerates to MMSB, the theoretical results under DCMM are consistent with those under MMSB. Using the SCSTC criterion, we find that the reasons behind the inconsistent phenomena are the sup-optimality of the original theoretical upper bounds on error rates for SPACL as well as its extended version, and the usage of a regularization version of the adjacency matrix when building theoretical results for spectral methods designed to detect nodes labels for a non-mixed network. The processes of finding these inconsistent phenomena, sub-optimality theoretical results on error rates and the formation mechanism of these inconsistent phenomena guarantee the usefulness of the SCSTC criterion. As shown by Remark 8, the theoretical results of some previous works can be improved by applying this criterion. Using SCSTC, we find that spectral methods considered in [26,41,43,44,48,49,50,67,68] achieve thresholds in Equations (1)–(3), and this conclusion is verified by both theoretical analysis and the numerical results in this paper. A limitation of this criterion is that it is only used for studying the consistency of spectral methods for a standard network with a constant number of communities. It would be interesting to develop a more general criterion that can study the consistency of all methods besides spectral methods, and models besides those can degenerate to SBM for a non-standard network with large *K*. Finally, we hope that the SCSTC criterion developed in this paper can be widely applied to build and compare theoretical results for spectral methods in the community detection area and that the thresholds in Equations (1)–(3) can be seen as benchmark thresholds for spectral methods.   

## Figures and Tables

**Table 1:** Comparison of separation condition and sharp threshold. Details of this table are given in Section 4. The classical result on separation condition given in Corollary 1 of [59] is $\sqrt{\frac{\log(n)}{n}}$ (i.e., Equation (1)). The classical result on sharp threshold is $\frac{\log(n)}{n}$ (i.e., Equation (3)) given in [61], Theorem 4.6 [62] and the first bullet in Section 2.5 [53]. In this paper, *n* is the number of nodes in a network, *A* is the adjacency matrix, Ω is the expectation of *A* under some models, $A_{\mathrm{re}}$ is a regularization of *A*, ρ is the sparsity parameter such that $\rho \geq \max_{i,j}\Omega \left(i,j\right)$ and it controls the overall sparsity of a network, ∥·∥ denotes spectral norm, and ξ>1.

	Model	Separation Condition	Sharp Threshold
Ours using $∥A_{\mathrm{re}}-\Omega ∥\leq C\sqrt{\rho n}$	MMSB&DCMM	$\sqrt{\frac{\log(n)}{n}}$	$\frac{\log(n)}{n}$
Ours using $∥A-\Omega ∥\leq C\sqrt{\rho n\log(n)}$	MMSB&DCMM	$\sqrt{\frac{\log(n)}{n}}$	$\frac{\log(n)}{n}$
Ref. [41] using $∥A_{\mathrm{re}}-\Omega ∥\leq C\sqrt{\rho n}$ (original)	DCMM	$\sqrt{\frac{\log(n)}{n}}$	$\frac{\log(n)}{n}$
Ref. [41] using $∥A-\Omega ∥\leq C\sqrt{\rho n\log(n)}$	DCMM	$\sqrt{\frac{\log(n)}{n}}$	$\frac{\log(n)}{n}$
Refs. [43,44] using $∥A_{\mathrm{re}}-\Omega ∥\leq C\sqrt{\rho n}$ (original)	MMSB&DCMM	$\frac{\log^{\xi }\left(n\right)}{\sqrt{n}}$	$\frac{\log^{2\xi }\left(n\right)}{n}$
Refs. [43,44] using $∥A-\Omega ∥\leq C\sqrt{\rho n\log(n)}$	MMSB&DCMM	$\frac{\log^{\xi +0.5}\left(n\right)}{\sqrt{n}}$	$\frac{\log^{2\xi +1}\left(n\right)}{n}$
Ref. [30] using $∥A_{\mathrm{re}}-\Omega ∥\leq C\sqrt{\rho n}$ (original)	SBM&DCSBM	$\sqrt{\frac{1}{n}}$	$\frac{1}{n}$
Ref. [30] using $∥A-\Omega ∥\leq C\sqrt{\rho n\log(n)\log(n)}$	SBM&DCSBM	$\sqrt{\frac{\log(n)}{n}}$	$\frac{\log(n)}{n}$

**Table 2:** Comparison of alternative separation condition, where the classical result on alternative separation condition is 1 (i.e., Equation (2)).

	Model	Alternative Separation Condition
Ours using $∥A_{\mathrm{re}}-\Omega ∥\leq C\sqrt{\rho n}$	MMSB&DCMM	1
Ours using $∥A-\Omega ∥\leq C\sqrt{\rho n\log(n)}$	MMSB&DCMM	1
Ref. [41] using $∥A_{\mathrm{re}}-\Omega ∥\leq C\sqrt{\rho n}$ (original)	DCMM	1
Ref. [41] using $∥A-\Omega ∥\leq C\sqrt{\rho n\log(n)}$	DCMM	1
Refs. [43,44] using $∥A_{\mathrm{re}}-\Omega ∥\leq C\sqrt{\rho n}$ (original)	MMSB&DCMM	$\log^{\xi -0.5}\left(n\right)$
Refs. [43,44] using $∥A-\Omega ∥\leq C\sqrt{\rho n\log(n)}$	MMSB&DCMM	$\log^{\xi }\left(n\right)$
Ref. [30] using $∥A_{\mathrm{re}}-\Omega ∥\leq C\sqrt{\rho n}$ (original)	SBM&DCSBM	$\sqrt{\frac{1}{\log(n)}}$
Ref. [30] using $∥A-\Omega ∥\leq C\sqrt{\rho n\log(n)\log(n)}$	SBM&DCSBM	1

**Table 3:** Comparison of error rates between our Theorem 1 and Theorem 3.2 [44] under $MMSB_{n}\left(K,\tilde{P},\Pi ,\rho \right)$. The dependence on *K* is obtained when $\kappa \left(\Pi^{\prime }\Pi \right)=O\left(1\right)$. For comparison, we have adjusted the $l_{2}$ error rates of Theorem 3.2 [44] into $l_{1}$ error rates. Note that as analyzed in the first bullet given after Lemma 2, whether using $∥A-\Omega ∥\leq C\sqrt{\rho n\log(n)}$ or $∥A_{\mathrm{re}}-\Omega ∥\leq C\sqrt{\rho n}$ does not change our ϖ, and has no influence on bound in Theorem 1. For [44], using $∥A_{\mathrm{re}}-\Omega ∥\leq \sqrt{\rho n}$, the power of log(n) in their Theorem 3.2 is ξ; using $∥A-\Omega ∥\leq \sqrt{\rho n\log(n)}$, the power of log(n) in their Theorem 3.2 is ξ+0.5.

	ρn	$\sigma_{K}\left(\Omega \right)$	$\lambda_{K}\left(\Pi^{\prime }\Pi \right)$	Dependence on *K*	Dependence on log(n)
Ours	≥log(n)	$⪰\sqrt{\rho n\log(n)}$	>0	$K^{2}$	$\log^{0.5}\left(n\right)$
[44]	$\geq \log^{2\xi }\left(n\right)$	$⪰\sqrt{\rho n}\log^{\xi }\left(n\right)$	≥1/ρ	$K^{2.5}$	$\log^{\xi }\left(n\right)$

**Table 4:** Comparison of error rates between our Theorem 2 and Theorem 3.2 [43] under $DCMM_{n}\left(K,P,\Pi ,\Theta \right)$. The dependence on *K* is obtained when $\kappa \left(\Pi^{\prime }\Pi \right)=O\left(1\right)$. For comparison, we adjusted the $l_{2}$ error rates of Theorem 3.2 [43] into $l_{1}$ error rates. Since Theorem 2 enjoys the same separation condition and sharp threshold as Theorem 1, and Theorem 3.2 [43] enjoys the same separation condition and sharp threshold as Theorem 3.2 [44], we do not report them in this table. Note that as analyzed in Remark 11, whether using $∥A-\Omega ∥\leq C\sqrt{\theta_{\max}{∥\theta ∥}_{1}\log\left(n\right)}$ or $∥A_{\mathrm{re}}-\Omega ∥\leq C\sqrt{\theta_{\max}{∥\theta ∥}_{1}}$ does not change our ϖ under DCMM, and has no influence on the results in Theorem 2. For [43], using $∥A_{\mathrm{re}}-\Omega ∥\sqrt{\theta_{\max}{∥\theta ∥}_{1}}$, the power of log(n) in their Theorem 3.2 is ξ; using $∥A-\Omega ∥\sqrt{\theta_{\max}{∥\theta ∥}_{1}\log\left(n\right)}$, the power of log(n) in their Theorem 3.2 is ξ+0.5.

	Π(i,:)	$\theta_{\max}{∥\theta ∥}_{1}$	$\sigma_{K}\left(\Omega \right)$	$\kappa (\Pi^{\prime }\Theta^{2}\Pi )$	Dependence on *K*	Dependence on log(n)
Ours	arbitrary	≥log(n)	$⪰\theta_{\max}\sqrt{n\log(n)}$	≥1	$K^{6}$	$\log^{0.5}\left(n\right)$
[43]	iid from Dirichlet	$\geq \log^{2\xi }\left(n\right)$	$⪰\theta_{\max}\sqrt{n}\log^{\xi }\left(n\right)$	=O(1)	$K^{6.5}$	$\log^{\xi }\left(n\right)$

## Data Availability

Not applicable.

## Abbreviations

The following abbreviations are used in this manuscript:

SCSTC	separation condition and sharp threshold criterion
SBM	stochastic blockmodels
DCSBM	degree corrected stochastic blockmodel
MMSB	mixed membership stochastic blockmodel
DCMM	degree corrected mixed membership model
SBMO	stochastic blockmodel with overlap
OCCAM	overlapping continuous community assignment model
RSC	regularized spectral clustering
SCORE	spectral clustering on ratios-of-eigenvectors
SPACL	sequential projection after cleaning
ER	Erdös–Rényi
IS	ideal simplex
IC	ideal cone
SP	successive projection algorithm
oPCA	ordinary principle component analysis
nPCA	normalized principle component analysis

## Appendix B. Vertex Hunting Algorithms

The SP algorithm is written as below.

**Algorithm A1** Successive projection (SP) [51]
**Require:** Near-separable matrix $Y_{sp}=S_{sp}M_{sp}+Z_{sp}\in R_{+}^{m\times n}$ , where $S_{sp},M_{sp}$ should satisfy Assumption 1 [51], the number *r* of columns to be extracted. **Ensure:** Set of indices K such that Y(K,:)≈S (up to permutation) 1: Let $R=Y_{sp},K=\left\{\right\},k=1$. 2: **While**R≠0 and k≤r **do** 3: $k_{*}={\mathrm{argmax}}_{k}{∥R\left(k,:\right)∥}_{F}$. 4: $u_{k}=R\left(k_{*},:\right)$. 5: $R\leftarrow (I-\frac{u_{k}u_{k}^{\prime }}{∥u_{k}{∥}_{F}^{2}})R$. 6: $K=K\cup \{k_{*}\}$. 7: k=k+1. 8: **end while**

Based on Algorithm A1, the following theorem is Theorem 1.1 in [51], and it is also the Lemma VII.1 in [44]. This theorem provides the bound between the corner matrix $S_{sp}$ and its estimated version returned by letting $Y_{sp}$ be the input of the SP algorithm when $M_{sp}^{\prime }S_{sp}^{\prime }$ enjoys the ideal simplex structure.

**Theorem** **A1.**
*Fix m≥r and n≥r. Consider a matrix $Y_{sp}=S_{sp}M_{sp}+Z_{sp}$, where $S_{sp}\in R^{m\times r}$ has a full column rank, $M_{sp}\in R^{r\times n}$ is a non-negative matrix such that the sum of each column is at most 1, and $Z_{sp}=\left[Z_{sp,1},\ldots ,Z_{sp,n}\right]\in R^{m\times n}$. Suppose that $M_{sp}$ has a submatrix equal to $I_{r}$. Write $\epsilon \leq \max_{1\leq i\leq n}{∥Z_{sp,i}∥}_{F}$. Suppose $\epsilon =O(\frac{\sigma_{\min}\left(S_{sp}\right)}{\sqrt{r}\kappa^{2}\left(S_{sp}\right)})$, where $\sigma_{\min}\left(S_{sp}\right)$ and $\kappa (S_{sp})$ are the minimum singular value and condition number of $S_{sp}$, respectively. If we apply the SP algorithm to columns of $Y_{sp}$, then it outputs an index set K⊂{1,2,…,n} such that |K|=r and $\max_{1\leq k\leq r}\min_{j\in K}{∥S_{sp}\left(:,k\right)-Y_{sp}\left(:,j\right)∥}_{F}=O\left(\epsilon \kappa^{2}\left(S_{sp}\right)\right)$, where $S_{sp}\left(:,k\right)$ is the k-th column of $S_{sp}$.*

For the ideal SPACL algorithm, since inputs of the ideal SPACL are Ω and *K*, we see that the inputs of SP algorithm are *U* and *K*. Let $m=K,r=K,Y_{sp}=U^{\prime },Z_{sp}=U^{\prime }-U^{\prime }\equiv 0,S_{sp}=U^{\prime }\left(I,:\right),$ and $M_{sp}=\Pi^{\prime }$. Then, we have $\max_{i\in [n]}{∥U\left(i,:\right)-U\left(i,:\right)∥}_{F}=0$. By Theorem A1, the SP algorithm returns I up to permutation when the input is *U*, assuming there are *K* communities. Since U=ΠU(I,:) under $MMSB_{n}\left(K,P,\Pi ,\rho \right)$, we see that U(i,:)=U(j,:) as long as Π(i,:)=Π(j,:). Therefore, though I may be different up to the permutation, U(I,:) is unchanged. Therefore, following the four steps of the ideal SPACL algorithm, we see that it exactly returns Π.

Algorithm A2 below is the SVM-cone algorithm provided in [43].

**Algorithm A2** SVM-cone [43]
**Require:**$\hat{S}\in R^{n\times m}$ with rows have unit $l_{2}$ norm, number of corners *K*, estimated distance corners from hyperplane γ. **Ensure:** The near-corner index set $\hat{I}$. 1: Run one-class SVM on $\hat{S}\left(i,:\right)$ to get $\hat{w}$ and $\hat{b}$ 2: Run k-means algorithm to the set $\left\{\hat{S}\left(i,:\right)|\hat{S}\left(i,:\right)\hat{w}\leq \hat{b}+\gamma \right\}$ that are close to the hyperplane into *K* clusters 3: Pick one point from each cluster to get the near-corner set $\hat{I}$

As suggested in [43], we can start γ=0 and incrementally increase it until *K* distinct clusters are found. Meanwhile, for the ideal SVM-cone-DCMMSB algorithm, when setting $U_{*}$ and *K* as the inputs of the SVM-cone algorithms, since $∥U_{*}-U_{*}{∥}_{2\to \infty }=0$, Lemma F.1. [43] guarantees that SVM-cone algorithm returns I up to permutation. Since $U_{*}=YU_{*}\left(I,:\right)$ by Lemma 3 under $DCMM_{n}\left(K,P,\Pi ,\Theta \right)$, we have $U_{*}\left(i,:\right)=U_{*}\left(j,:\right)$ when Π(i,:)=Π(j,:) by basic algebra, which gives that $U_{*}\left(I,:\right)$ is unchanged though I may be different up to permutation. Therefore, the ideal SVM-cone-DCMMSB exactly recovers Π.

## Appendix C. Proof of Consistency under MMSB

### Appendix C.1. Proof of Lemma 1

**Proof.** We apply Theorem 1.4 (Bernstein inequality) in [70] to bound ∥A−Ω∥, and this theorem is written as shown below. **Theorem** **A2.**
*Consider a finite sequence $\left\{X_{k}\right\}$ of independent, random, self-adjoint matrices with dimension d. Assume that each random matrix satisfies*

$$\begin{array}{c} E\left[X_{k}\right]=0,\mathrm{and}\;\lambda_{\max}\left(X_{k}\right)\leq R\;\mathrm{almost}\;\mathrm{surely}. \end{array}$$

*Then, for all t≥0,*

$$\begin{array}{c} P\left(\lambda_{\max}\left(\sum_{k}X_{k}\right)\geq t\right)\leq d\cdot \exp\left(\frac{-t^{2}/2}{\sigma^{2}+Rt/3}\right), \end{array}$$

*where $\sigma^{2}:=∥\sum_{k}E\left[X_{k}^{2}\right]∥$.*
Let $e_{i}$ be an n×1 vector, where $e_{i}\left(i\right)=1$ and 0 elsewhere, for i∈[n]. For convenience, set W=A−Ω. Then we can write *W* as $W=\sum_{i=1}^{n}\sum_{j=1}^{n}W\left(i,j\right)e_{i}e_{j}^{\prime }$. Set $W^{\left(i,j\right)}$ as the n×n matrix such that $W^{\left(i,j\right)}=W\left(i,j\right)\left(e_{i}e_{j}^{\prime }+e_{j}e_{i}^{\prime }\right)$, which gives $W=\sum_{1\leq i<j\leq n}W^{\left(i,j\right)}$ where $E[W^{\left(i,j\right)}]=0$ and

$$\begin{array}{cc} ∥W^{\left(i,j\right)}∥ & =\;∥W\left(i,j\right)\left(e_{i}e_{j}^{\prime }+e_{j}e_{i}\right)∥\;=|W\left(i,j\right)|∥\left(e_{i}e_{j}^{\prime }+e_{j}e_{i}^{\prime }\right)∥\;=|W\left(i,j\right)|\;=|A\left(i,j\right)-\Omega \left(i,j\right)|\leq 1. \end{array}$$

For the variance parameter $\sigma^{2}:=∥\sum_{1\leq i<j\leq n}E\left[{\left(W^{\left(i,j\right)}\right)}^{2}\right]∥$. We bound $E(W^{2}\left(i,j\right))$ as shown below:

$$\begin{array}{cc} E(W^{2}\left(i,j\right)) & =E({\left(A\left(i,j\right)-\Omega \left(i,j\right)\right)}^{2})=\mathrm{Var}\left(A\left(i,j\right)\right)=\Omega \left(i,j\right)\left(1-\Omega \left(i,j\right)\right)\leq \Omega \left(i,j\right)\\ \; & =\rho \Pi \left(i,:\right)\tilde{P}\Pi^{\prime }\left(j,:\right)\leq \rho . \end{array}$$

Next we bound $\sigma^{2}$ as shown below:

$$\begin{array}{cc} \sigma^{2} & =∥\sum_{1\leq i<j\leq n}E\left(W^{2}\left(i,j\right)\right)\left(e_{i}e_{j}^{\prime }+e_{j}e_{i}^{\prime }\right)\left(e_{i}e_{j}^{\prime }+e_{j}e_{i}^{\prime }\right)∥=∥\sum_{1\leq i<j\leq n}E\left[W^{2}\left(i,j\right)\left(e_{i}e_{i}^{\prime }+e_{j}e_{j}^{\prime }\right)\right]∥\\ \; & \leq \max_{1\leq i\leq n}\left|\sum_{j=1}^{n}E\left(W^{2}\left(i,j\right)\right)\right|\leq \max_{1\leq i\leq n}\sum_{j=1}^{n}\rho =\rho n. \end{array}$$

Set $t=\frac{\alpha +1+\sqrt{\left(\alpha +1)(\alpha +19\right)}}{3}\sqrt{\rho n\log(n)}$ for any α>0, combine Theorem A2 with $\sigma^{2}\leq \rho n,R=1,d=n$, and we have

$$\begin{array}{cc} P(∥W∥\geq t) & =P(∥\sum_{1\leq i<j\leq n}W^{\left(i,j\right)}∥\geq t)\leq n\cdot \exp\left(\frac{-t^{2}/2}{\sigma^{2}+Rt/3}\right)\\ \; & \leq n\cdot \exp\left(\frac{-(\alpha +1)\log(n)}{\frac{18}{{\left(\sqrt{\alpha +1}+\sqrt{\alpha +19}\right)}^{2}}+\frac{2\sqrt{\alpha +1}}{\sqrt{\alpha +1}+\sqrt{\alpha +19}}\sqrt{\frac{\log(n)}{\rho n}}}\right)\leq \frac{1}{n^{\alpha }}, \end{array}$$

where we use Assumption (A1) such that $\frac{18}{{\left(\sqrt{\alpha +1}+\sqrt{\alpha +19}\right)}^{2}}+\frac{2\sqrt{\alpha +1}}{\sqrt{\alpha +1}+\sqrt{\alpha +19}}\sqrt{\frac{\log(n)}{\rho n}}\leq$$\frac{18}{{\left(\sqrt{\alpha +1}+\sqrt{\alpha +19}\right)}^{2}}$$+\frac{2\sqrt{\alpha +1}}{\sqrt{\alpha +1}+\sqrt{\alpha +19}}=1$. □

### Appendix C.2. Proof of Lemma 2

**Proof.** Let $H={\hat{U}}^{\prime }U$, and $H=U_{H}\Sigma_{H}V_{H}^{\prime }$ be the SVD decomposition of $H_{\hat{U}}$ with $U_{H},V_{H}\in R^{n\times K}$, where $U_{H}$ and $V_{H}$ represent respectively the left and right singular matrices of *H*. Define $\mathrm{sgn}\left(H\right)=U_{H}V_{H}^{\prime }$. Since E(A(i,j)−Ω(i,j))=0, $E[{\left(A\left(i,j\right)-\Omega \left(i,j\right)\right)}^{2}]\leq \rho$ by the proof of Lemma 1, $\frac{1}{\sqrt{\rho n/(\mu \log(n))}}\leq O\left(1\right)$ holds by Assumption (A1) where μ is the incoherence parameter defined as $\mu =\frac{{n∥U∥}_{2\to \infty }^{2}}{K}$. By Theorem 4.2 [64], with high probability, we have

$$\begin{array}{c} ∥\hat{U}{\mathrm{sgn}\left(H\right)-U∥}_{2\to \infty }\leq C\frac{\sqrt{K\rho }\left(\kappa \left(\Omega \right)\sqrt{\mu }+\sqrt{\log(n)}\right)}{\sigma_{K}\left(\Omega \right)}, \end{array}$$

provided that $c_{1}\sigma_{K}\left(\Omega \right)\geq \sqrt{\rho n\log(n)}$ for some sufficiently small constant $c_{1}$. By Lemma 3.1 of [44], we know that ${∥U∥}_{2\to \infty }^{2}\leq \frac{1}{\lambda_{K}\left(\Pi^{\prime }\Pi \right)}$, which gives

(A1) $$\begin{array}{c} ∥\hat{U}{\mathrm{sgn}\left(H\right)-U∥}_{2\to \infty }\leq C\frac{\sqrt{K\rho }\left(\kappa \left(\Omega \right)\sqrt{\frac{n}{K\lambda_{K}\left(\Pi^{\prime }\Pi \right)}}+\sqrt{\log(n)}\right)}{\sigma_{K}\left(\Omega \right)}. \end{array}$$

**Remark** **A1.**
*By Theorem 4.2 of [65], when $\sigma_{K}\left(\Omega \right)\geq 4{∥A-\Omega ∥}_{\infty }$, we have*

$$\begin{array}{c} ∥\hat{U}{\mathrm{sgn}\left(H\right)-U∥}_{2\to \infty }\leq 14\frac{{∥A-\Omega ∥}_{\infty }}{\sigma_{K}\left(\Omega \right)}{∥U∥}_{2\to \infty }. \end{array}$$

*By Lemma 3.1 [44], we have*

$$\begin{array}{c} ∥\hat{U}{\mathrm{sgn}\left(H\right)-U∥}_{2\to \infty }\leq 14\frac{{∥A-\Omega ∥}_{\infty }}{\sigma_{K}\left(\Omega \right)}\sqrt{\frac{1}{\lambda_{K}\left(\Pi^{\prime }\Pi \right)}}. \end{array}$$

*Unlike Lemma V.1 [44] which bounds ${∥A-\Omega ∥}_{\infty }$ via the Chernoff bound and obtains ${∥A-\Omega ∥}_{\infty }\leq C\rho n$ with high probability, we bound ${∥A-\Omega ∥}_{\infty }$ by the Bernstein inequality using a similar idea as Equation (C.67) of [41]. Let $y={\left(y_{1},y_{2},\ldots ,y_{n}\right)}^{\prime }$ be any n×1 vector; by Equation (C.67) [41], we know that with an application of the Bernstein inequality, for any t≥0 and i∈[n], we have*

$$\begin{array}{c} P(|\sum_{j=1}^{n}\left(A\left(i,j\right)-\Omega \left(i,j\right)\right)y\left(j\right)\left|>t\right)\leq 2\exp\left(-\frac{t^{2}/2}{\sum_{j=1}^{n}\Omega \left(i,j\right)y^{2}\left(j\right)+\frac{{t∥y∥}_{\infty }}{3}}\right). \end{array}$$

*By the proof of Lemma 1, we have Ω(i,j)≤ρ. Set y(j) as *1* or −1 such that (A(i,j)−Ω(i,j))y(j)=|A(i,j)−Ω(i,j)|, we have*

$$\begin{array}{c} {P(∥A-\Omega ∥}_{\infty }>t)\leq 2\exp\left(-\frac{t^{2}/2}{\rho n+\frac{t}{3}}\right). \end{array}$$

*Set $t=\frac{\alpha +1+\sqrt{\left(\alpha +1)(\alpha +19\right)}}{3}\sqrt{\rho n\log(n)}$ for any α>0, by Assumption (A1), we have*

$$\begin{array}{c} {P(∥A-\Omega ∥}_{\infty }>t)\leq 2\exp\left(-\frac{t^{2}/2}{\rho n+\frac{t}{3}}\right)\leq n^{-\alpha }. \end{array}$$

*Hence, when $\sigma_{K}\left(\Omega \right)\geq C_{0}\sqrt{\rho n\log(n)}$ where $C_{0}=4\frac{\alpha +1+\sqrt{\left(\alpha +1)(\alpha +19\right)}}{3}$, with probability at least $1-o(n^{-\alpha })$,*

$$\begin{array}{c} ∥\hat{U}\mathrm{sgn}\left(H_{\hat{U}}\right){-U∥}_{2\to \infty }\leq C\frac{\sqrt{\rho n\log(n)}}{\sigma_{K}\left(\Omega \right)}\sqrt{\frac{1}{\lambda_{K}\left(\Pi^{\prime }\Pi \right)}}. \end{array}$$

*Note that when $\lambda_{K}\left(\Pi^{\prime }\Pi \right)=O\left(\frac{n}{K}\right)$, the above bound turns to be $C\frac{\sqrt{\rho K\log(n)}}{\sigma_{K}\left(\Omega \right)}$, which is consistent with that of Equation (A1). Also note that this bound $\frac{\sqrt{\rho n\log(n)}}{\sigma_{K}\left(\Omega \right)}\sqrt{\frac{1}{\lambda_{K}\left(\Pi^{\prime }\Pi \right)}}$ is sharper than the $\frac{\rho n}{\sigma_{K}\left(\Omega \right)}\sqrt{\frac{1}{\lambda_{K}\left(\Pi^{\prime }\Pi \right)}}$ of Lemma V.1 [44] by Assumption (A1).*

Since $\hat{U}$ and *U* have orthonormal columns, now we are ready to bound $∥\hat{U}{\hat{U}}^{\prime }-UU^{\prime }{∥}_{2\to \infty }$:

$$\begin{array}{cc} \; & ∥\hat{U}{\hat{U}}^{\prime }-UU^{\prime }{∥}_{2\to \infty }=\max_{i\in [n]}{∥e_{i}^{\prime }\left(UU^{\prime }-\hat{U}{\hat{U}}^{\prime }\right)∥}_{F}\\ \; & =\max_{i\in [n]}{∥e_{i}^{\prime }\left(UU^{\prime }-\hat{U}\mathrm{sgn}\left(H\right)U^{\prime }+\hat{U}\mathrm{sgn}\left(H\right)U^{\prime }-\hat{U}{\hat{U}}^{\prime }\right)∥}_{F}\\ \; & \leq \max_{i\in [n]}∥e_{i}^{\prime }\left(U-\hat{U}\mathrm{sgn}\left(H\right)\right)U^{\prime }{∥}_{F}+\max_{i\in [n]}{∥e_{i}^{\prime }\hat{U}\left(\mathrm{sgn}\left(H\right)U^{\prime }-{\hat{U}}^{\prime }\right)∥}_{F}\\ \; & =\max_{i\in [n]}∥e_{i}^{\prime }\left(U-\hat{U}\mathrm{sgn}\left(H\right)\right){∥}_{F}+\max_{i\in [n]}{∥\hat{U}\left(\mathrm{sgn}\left(H\right)U^{\prime }-{\hat{U}}^{\prime }\right)e_{i}∥}_{F}\\ \; & =\max_{i\in [n]}∥e_{i}^{\prime }\left(U-\hat{U}\mathrm{sgn}\left(H\right)\right){∥}_{F}+\max_{i\in [n]}{∥\left(\mathrm{sgn}\left(H\right)U^{\prime }-{\hat{U}}^{\prime }\right)e_{i}∥}_{F}\\ \; & =\max_{i\in [n]}∥e_{i}^{\prime }\left(U-\hat{U}\mathrm{sgn}\left(H\right)\right){∥}_{F}+\max_{i\in [n]}{∥e_{i}^{\prime }\left(U{\left(\mathrm{sgn}\left(H\right)\right)}^{\prime }-\hat{U}\right)∥}_{F}\\ \; & =\max_{i\in [n]}∥e_{i}^{\prime }\left(U-\hat{U}\mathrm{sgn}\left(H\right)\right){∥}_{F}+\max_{i\in [n]}{∥e_{i}^{\prime }\left(U-\hat{U}\mathrm{sgn}\left(H\right)\right)∥}_{F}\\ \; & =2\max_{i\in [n]}∥e_{i}^{\prime }\left(U-\hat{U}\mathrm{sgn}\left(H\right)\right){∥}_{F}=2{∥U-\hat{U}\mathrm{sgn}\left(H\right)∥}_{2\to \infty }\\ \; & \leq C\frac{\sqrt{K}\left(\kappa \left(\Omega \right)\sqrt{\frac{n}{K\lambda_{K}\left(\Pi^{\prime }\Pi \right)}}+\sqrt{\log(n)}\right)}{\sigma_{K}\left(\tilde{P}\right)\sqrt{\rho }\lambda_{K}\left(\Pi^{\prime }\Pi \right)}, \end{array}$$

where the last inequality holds since $\sigma_{K}\left(\Omega \right)\geq \sigma_{K}\left(\tilde{P}\right)\rho \lambda_{K}\left(\Pi^{\prime }\Pi \right)$ under $MMSB_{n}\left(K,\tilde{P},\Pi ,\rho \right)$ by Lemma II.4 [44] This bound is $C\frac{\sqrt{n\log(n)}}{\sigma_{K}\left(\tilde{P}\right)\sqrt{\rho }\lambda_{K}^{1.5}\left(\Pi^{\prime }\Pi \right)}$ if we use Theorem 4.2 of [65].

**Remark** **A2.**
*By Theorem 4.5 [77], we have $∥\hat{U}{\hat{U}}^{\prime }-UU^{\prime }{∥}_{2\to \infty }\leq \sqrt{n}(∥\hat{U}{∥}_{2\to \infty }+{∥U∥}_{2\to \infty })∥U-\hat{U}{\mathrm{sgn}\left(H\right)∥}_{2\to \infty }\leq \sqrt{n}(∥U-\hat{U}{\mathrm{sgn}\left(H\right)∥}_{2\to \infty }+{2∥U∥}_{2\to \infty })∥U-\hat{U}{\mathrm{sgn}\left(H\right)∥}_{2\to \infty }$ $\leq \sqrt{n}(∥U-\hat{U}{\mathrm{sgn}\left(H\right)∥}_{2\to \infty }+\frac{2}{\sqrt{\lambda_{K}\left(\Pi^{\prime }\Pi \right)}})∥U-\hat{U}{\mathrm{sgn}\left(H\right)∥}_{2\to \infty }=O(\frac{2\sqrt{n}}{\sqrt{\lambda_{K}\left(\Pi^{\prime }\Pi \right)}}∥U-\hat{U}\mathrm{sgn}\left(H\right){∥}_{2\to \infty })$ . Sure our bound $∥\hat{U}{\hat{U}}^{\prime }-UU^{\prime }{∥}_{2\to \infty }\leq 2{∥U-\hat{U}\mathrm{sgn}\left(H\right)∥}_{2\to \infty }$ enjoys concise form. In particular, when $\lambda_{K}\left(\Pi^{\prime }\Pi \right)=O\left(\frac{n}{K}\right)$ and K=O(1), the two bounds give that $∥\hat{U}{\hat{U}}^{\prime }-UU^{\prime }{∥}_{2\to \infty }=O(∥U-\hat{U}\mathrm{sgn}\left(H\right){∥}_{2\to \infty })$, which provides same error bound of the estimated memberships given in Corollary 1.*

□

### Appendix C.3. Proof of Theorem 1

**Proof.** Follow almost the same proof as Equation (3) of [44]. For i∈[n], there exists a permutation matrix $P\in R^{K\times K}$ such that

(A2) $$\begin{array}{c} ∥e_{i}^{\prime }\left(\hat{Z}-ZP\right){∥}_{F}=O\left(\varpi \kappa \left(\Pi^{\prime }\Pi \right)\sqrt{K\lambda_{1}\left(\Pi^{\prime }\Pi \right)}\right). \end{array}$$

Note that the bound in Equation (A2) is $\sqrt{K}$ times the bound in Equation (3) of [44], and this is because in Equation (3) of [44], (in the language of Ref. [44]) $∥{\hat{V}}_{p}^{-1}∥$ denotes the Frobenius norm of ${\hat{V}}_{p}^{-1}$ instead of the spectral norm. Since $∥{\hat{V}}_{p}^{-1}{∥}_{F}\leq \frac{\sqrt{K}}{\sigma_{K}\left({\hat{V}}_{p}\right)}$, the bound in Equation (3) [44] should multiply $\sqrt{K}$. Recall that $Z=\Pi ,\Pi \left(i,:\right)=\frac{Z(i,:)}{{∥Z\left(i,:\right)∥}_{1}},\hat{\Pi }\left(i,:\right)=\frac{\hat{Z}\left(j,:\right)}{∥\hat{Z}{\left(j,:\right)∥}_{1}}$, for i∈[n], since

$$\begin{array}{cc} ∥e_{i}^{\prime }\left(\hat{\Pi }-\Pi P\right){∥}_{1} & =∥\frac{e_{i}^{\prime }\hat{Z}}{∥e_{i}^{\prime }\hat{Z}{∥}_{1}}-\frac{e_{i}^{\prime }ZP}{∥e_{i}^{\prime }{ZP∥}_{1}}{∥}_{1}={∥\frac{e_{i}^{\prime }\hat{Z}∥e_{i}^{\prime }{Z∥}_{1}-e_{i}^{\prime }ZP{∥e_{i}^{\prime }\hat{Z}∥}_{1}}{∥e_{i}^{\prime }\hat{Z}{∥}_{1}{∥e_{i}^{\prime }Z∥}_{1}}∥}_{1}\\ & \leq \frac{∥e_{i}^{\prime }\hat{Z}∥e_{i}^{\prime }{Z∥}_{1}-e_{i}^{\prime }\hat{Z}∥e_{i}^{\prime }\hat{Z}{∥}_{1}{∥}_{1}+∥e_{i}^{\prime }\hat{Z}∥e_{i}^{\prime }\hat{Z}{∥}_{1}-e_{i}^{\prime }ZP∥e_{i}^{\prime }\hat{Z}{{∥}_{1}∥}_{1}}{∥e_{i}^{\prime }\hat{Z}{∥}_{1}{∥e_{i}^{\prime }Z∥}_{1}}\\ & =\frac{|∥e_{i}^{\prime }{Z∥}_{1}-∥e_{i}^{\prime }\hat{Z}{∥}_{1}|+∥e_{i}^{\prime }\hat{Z}-e_{i}^{\prime }{ZP∥}_{1}}{∥e_{i}^{\prime }{Z∥}_{1}}\leq \frac{2∥e_{i}^{\prime }\left(\hat{Z}-ZP\right){∥}_{1}}{∥e_{i}^{\prime }{Z∥}_{1}}\\ \; & =\frac{2∥e_{i}^{\prime }\left(\hat{Z}-ZP\right){∥}_{1}}{∥e_{i}^{\prime }{\Pi ∥}_{1}}=2∥e_{i}^{\prime }\left(\hat{Z}-ZP\right){∥}_{1}\leq 2\sqrt{K}{∥e_{i}^{\prime }\left(\hat{Z}-ZP\right)∥}_{F}\\ \; & =O(\varpi K\kappa \left(\Pi^{\prime }\Pi \right)\sqrt{\lambda_{1}\left(\Pi^{\prime }\Pi \right)}). \end{array}$$

□

### Appendix C.4. Proof of Corollary 1

**Proof.** Under the conditions of Corollary 1, we have

$$\begin{array}{c} \max_{i\in [n]}{∥e_{i}^{\prime }\left(\hat{\Pi }-\Pi P\right)∥}_{1}=O\left(\varpi \sqrt{n}\right). \end{array}$$

Under the conditions of Corollary 1, Lemma 2 gives $\varpi =O(\frac{1}{\sigma_{K}\left(\tilde{P}\right)}\frac{1}{\sqrt{n}}\sqrt{\frac{\log(n)}{\rho n}})$, which gives that

$$\begin{array}{c} \max_{i\in [n]}{∥e_{i}^{\prime }\left(\hat{\Pi }-\Pi P\right)∥}_{1}=O\left(\frac{1}{\sigma_{K}\left(\tilde{P}\right)}\sqrt{\frac{\log(n)}{\rho n}}\right). \end{array}$$

□

## Appendix D. Proof of Consistency under DCMM

### Appendix D.1. Proof of Lemma 3

**Proof.** Since $\Omega =U\Lambda U^{\prime }$, we have $U=\Omega U\Lambda^{-1}$ since $U^{\prime }U=I_{K}$. Recall that $\Omega =\Theta \Pi \tilde{P}\Pi^{\prime }\Theta$, we have $U=\Theta \Pi \tilde{P}\Pi^{\prime }\Theta U\Lambda^{-1}=\Theta \Pi B$, where we set $B=\tilde{P}\Pi^{\prime }\Theta U\Lambda^{-1}$ for convenience. Since U(I,:)=Θ(I,I)Π(I,:)B=Θ(I,I)B, we have $B=\Theta^{-1}\left(I,I\right)U\left(I,:\right)$. Set M=ΠB. Then we have U=ΘM, which gives that $U\left(i,:\right)=e_{i}^{\prime }U=\Theta \left(i,i\right)M\left(i,:\right)$ for i∈[n]. Therefore, $U_{*}\left(i,:\right)=\frac{U(i,:)}{{∥U\left(i,:\right)∥}_{F}}=\frac{M(i,:)}{{∥M\left(i,:\right)∥}_{F}}$, and combined with the fact that $B=\Theta^{-1}\left(I,I\right)U\left(I,:\right)\equiv \Theta^{-1}\left(I,I\right)N_{U}^{-1}\left(I,I\right)N_{U}\left(I,I\right)U\left(I,:\right)\equiv \Theta^{-1}\left(I,I\right)N_{U}^{-1}\left(I,I\right)U_{*}\left(I,:\right)$, we have

$$\begin{array}{c} U_{*}=\left[\begin{array}{c} \Pi \left(1,:\right)/{∥M\left(1,:\right)∥}_{F}\\ \Pi \left(2,:\right)/{∥M\left(2,:\right)∥}_{F}\\ \vdots \\ \Pi \left(n,:\right)/{∥M\left(n,:\right)∥}_{F} \end{array}\right]B=\left[\begin{array}{c} \Pi \left(1,:\right)/{∥M\left(1,:\right)∥}_{F}\\ \Pi \left(2,:\right)/{∥M\left(2,:\right)∥}_{F}\\ \vdots \\ \Pi \left(n,:\right)/{∥M\left(n,:\right)∥}_{F} \end{array}\right]\Theta^{-1}\left(I,I\right)N_{U}^{-1}\left(I,I\right)U_{*}\left(I,:\right). \end{array}$$

Therefore, we have

$$\begin{array}{c} Y=N_{M}\Pi \Theta^{-1}\left(I,I\right)N_{U}^{-1}\left(I,I\right), \end{array}$$

where $N_{M}$ is a diagonal matrix whose *i*-th diagonal entry is $\frac{1}{{∥M\left(i,:\right)∥}_{F}}$ for i∈[n]. □

### Appendix D.2. Proof of Lemma 4

**Proof.** Similar to the proof of Lemma 1, set W=A−Ω and $W^{\left(i,j\right)}=W\left(i,j\right)\left(e_{i}e_{j}^{\prime }+e_{j}e_{i}^{\prime }\right)$, we have $W=\sum_{1\leq i<j\leq n}W^{\left(i,j\right)}$, $E[W^{\left(i,j\right)}]=0$ and $∥W^{\left(i,j\right)}∥\leq 1$. Since

$$\begin{array}{cc} E(W^{2}\left(i,j\right)) & =E({\left(A\left(i,j\right)-\Omega \left(i,j\right)\right)}^{2})=\mathrm{Var}\left(A\left(i,j\right)\right)=\Omega \left(i,j\right)\left(1-\Omega \left(i,j\right)\right)\\ \; & \leq \Omega \left(i,j\right)=\theta \left(i\right)\theta \left(j\right)\Pi \left(i,:\right)\tilde{P}\Pi^{\prime }\left(j,:\right)\leq \theta \left(i\right)\theta \left(j\right){\tilde{P}}_{\max}, \end{array}$$

we have

$$\begin{array}{cc} \sigma^{2} & =∥\sum_{1\leq i<j\leq n}E\left(W^{2}\left(i,j\right)\right)\left(e_{i}e_{j}^{\prime }+e_{j}e_{i}^{\prime }\right)\left(e_{i}e_{j}^{\prime }+e_{j}e_{i}^{\prime }\right)∥=∥\sum_{1\leq i<j\leq n}E\left[W^{2}\left(i,j\right)\left(e_{i}e_{i}^{\prime }+e_{j}e_{j}^{\prime }\right)\right]∥\\ \; & \leq \max_{1\leq i\leq n}|\sum_{j=1}^{n}E\left(W^{2}\left(i,j\right)\right)|\leq \max_{1\leq i\leq n}\sum_{j=1}^{n}\theta \left(i\right)\theta \left(j\right){\tilde{P}}_{\max}\leq {\tilde{P}}_{\max}\theta_{\max}{∥\theta ∥}_{1}. \end{array}$$

Set $t=\frac{\alpha +1+\sqrt{\left(\alpha +1)(\alpha +19\right)}}{3}\sqrt{{\tilde{P}}_{\max}\theta_{\max}{∥\theta ∥}_{1}\log\left(n\right)}$ for any α>0, combine Theorem A2 with $\sigma^{2}\leq {\tilde{P}}_{\max}\theta_{\max}{∥\theta ∥}_{1},R=1,d=n$, we have

$$\begin{array}{cc} P(∥W∥\geq t) & =P(∥\sum_{1\leq i<j\leq n}W^{\left(i,j\right)}∥\geq t)\leq n\cdot \exp\left(\frac{-t^{2}/2}{\sigma^{2}+Rt/3}\right)\\ \; & \leq n\cdot \exp\left(\frac{-(\alpha +1)\log(n)}{\frac{18}{{\left(\sqrt{\alpha +1}+\sqrt{\alpha +19}\right)}^{2}}+\frac{2\sqrt{\alpha +1}}{\sqrt{\alpha +1}+\sqrt{\alpha +19}}\sqrt{\frac{\log(n)}{{\tilde{P}}_{\max}\theta_{\max}{∥\theta ∥}_{1}}}}\right)\leq \frac{1}{n^{\alpha }}, \end{array}$$

where we use Assumption (A2) such that $\frac{18}{{\left(\sqrt{\alpha +1}+\sqrt{\alpha +19}\right)}^{2}}+\frac{2\sqrt{\alpha +1}}{\sqrt{\alpha +1}+\sqrt{\alpha +19}}\sqrt{\frac{\log(n)}{{\tilde{P}}_{\max}\theta_{\max}{∥\theta ∥}_{1}}}\leq \frac{18}{{\left(\sqrt{\alpha +1}+\sqrt{\alpha +19}\right)}^{2}}+\frac{2\sqrt{\alpha +1}}{\sqrt{\alpha +1}+\sqrt{\alpha +19}}=1$. □

### Appendix D.3. Proof of Lemma 5

**Proof.** The proof is similar to that of Lemma 2, so we omit most details. Since E(A(i,j)−Ω(i,j))=0, $E\left[{\left(A\left(i,j\right)-\Omega \left(i,j\right)\right)}^{2}\right]\leq \theta \left(i\right)\theta \left(j\right){\tilde{P}}_{\max}\leq \theta_{\max}^{2}{\tilde{P}}_{\max}$, $\frac{1}{\theta_{\max}\sqrt{{\tilde{P}}_{\max}n/\left(\mu \log\left(n\right)\right)}}\leq O\left(1\right)$ holds by Assumption (A2) where $\mu =\frac{{n∥U∥}_{2\to \infty }^{2}}{K}$. By Theorem 4.2 [64], with high probability, we have

$$\begin{array}{c} ∥\hat{U}{\mathrm{sgn}\left(H\right)-U∥}_{2\to \infty }\leq C\frac{\theta_{\max}\sqrt{{\tilde{P}}_{\max}K}\left(\kappa \left(\Omega \right)\sqrt{\mu }+\sqrt{\log(n)}\right)}{\sigma_{K}\left(\Omega \right)}, \end{array}$$

provided that $c_{*}\sigma_{K}\left(\Omega \right)\geq \theta_{\max}\sqrt{{\tilde{P}}_{\max}n\log\left(n\right)}$ for some sufficiently small constant $c_{*}$. By Lemma H.1 of [43], we know that ${∥U∥}_{2\to \infty }^{2}\leq \frac{\theta_{\max}^{2}}{\lambda_{K}\left(\Pi^{\prime }\Theta^{2}\Pi \right)}\leq \frac{\theta_{\max}^{2}}{\theta_{\min}^{2}\lambda_{K}\left(\Pi^{\prime }\Pi \right)}$ under $DCMM_{n}\left(K,\tilde{P},\Pi ,\Theta \right)$, which gives

$$\begin{array}{c} ∥\hat{U}{\mathrm{sgn}\left(H\right)-U∥}_{2\to \infty }\leq C\frac{\theta_{\max}\sqrt{{\tilde{P}}_{\max}K}\left(\frac{\theta_{\max}\kappa \left(\Omega \right)}{\theta_{\min}}\sqrt{\frac{n}{K\lambda_{K}\left(\Pi^{\prime }\Pi \right)}}+\sqrt{\log(n)}\right)}{\sigma_{K}\left(\Omega \right)}, \end{array}$$

**Remark** **A3.**
*Similar to the proof of Lemma 2, by Theorem 4.2 of [65], when $\sigma_{K}\left(\Omega \right)\geq 4{∥A-\Omega ∥}_{\infty }$, we have*

$$\begin{array}{c} ∥\hat{U}{\mathrm{sgn}\left(H\right)-U∥}_{2\to \infty }\leq 14\frac{{∥A-\Omega ∥}_{\infty }}{\sigma_{K}\left(\Omega \right)}{∥U∥}_{2\to \infty }\leq \frac{14\theta_{\max}{∥A-\Omega ∥}_{\infty }}{\theta_{\min}\sigma_{K}\left(\Omega \right)\sqrt{\lambda_{K}\left(\Pi^{\prime }\Pi \right)}}. \end{array}$$

*Let $y={\left(y_{1},y_{2},\ldots ,y_{n}\right)}^{\prime }$ be any n×1 vector, and by the Bernstein inequality, for any t≥0 and i∈[n], we have*

$$\begin{array}{c} P(|\sum_{j=1}^{n}\left(A\left(i,j\right)-\Omega \left(i,j\right)\right)y\left(j\right)\left|>t\right)\leq 2\exp\left(-\frac{t^{2}/2}{\sum_{j=1}^{n}\Omega \left(i,j\right)y^{2}\left(j\right)+\frac{{t∥y∥}_{\infty }}{3}}\right). \end{array}$$

*By the proof of Lemma 4, we have $\Omega \left(i,j\right)\leq \theta \left(i\right)\theta \left(j\right){\tilde{P}}_{\max}$, which gives $\sum_{j=1}^{n}\Omega \left(i,j\right)\leq {\tilde{P}}_{\max}\theta_{\max}{∥\theta ∥}_{1}$. Set y(j) as *1* or −1 such that (A(i,j)−Ω(i,j))y(j)=|A(i,j)−Ω(i,j)|, we have*

$$\begin{array}{c} {P(∥A-\Omega ∥}_{\infty }>t)\leq 2\exp\left(-\frac{t^{2}/2}{{\tilde{P}}_{\max}\theta_{\max}{∥\theta ∥}_{1}+\frac{t}{3}}\right). \end{array}$$

*Set $t=\frac{\alpha +1+\sqrt{\left(\alpha +1)(\alpha +19\right)}}{3}\sqrt{{\tilde{P}}_{\max}\theta_{\max}{∥\theta ∥}_{1}\log\left(n\right)}$ for any α>0, by Assumption (A2), we have*

$$\begin{array}{c} {P(∥A-\Omega ∥}_{\infty }>t)\leq 2\exp\left(-\frac{t^{2}/2}{{\tilde{P}}_{\max}\theta_{\max}{∥\theta ∥}_{1}+\frac{t}{3}}\right)\leq n^{-\alpha }. \end{array}$$

*Hence, when $\sigma_{K}\left(\Omega \right)\geq C_{0}\sqrt{{\tilde{P}}_{\max}\theta_{\max}{∥\theta ∥}_{1}\log\left(n\right)}$ where $C_{0}=4\frac{\alpha +1+\sqrt{\left(\alpha +1)(\alpha +19\right)}}{3}$, with probability at least $1-o(n^{-\alpha })$,*

$$\begin{array}{c} ∥\hat{U}{\mathrm{sgn}\left(H\right)-U∥}_{2\to \infty }\leq C\frac{\theta_{\max}\sqrt{{\tilde{P}}_{\max}\theta_{\max}{∥\theta ∥}_{1}\log\left(n\right)}}{\theta_{\min}\sigma_{K}\left(\Omega \right)\sqrt{\lambda_{K}\left(\Pi^{\prime }\Pi \right)}}. \end{array}$$

*Meanwhile, since $\sqrt{{\tilde{P}}_{\max}\theta_{\max}{∥\theta ∥}_{1}\log\left(n\right)}\leq \theta_{\max}\sqrt{{\tilde{P}}_{\max}n\log\left(n\right)}$, for convenience, we let the lower bound requirement of $\sigma_{K}\left(\Omega \right)$ be $C\theta_{\max}\sqrt{{\tilde{P}}_{\max}n\log\left(n\right)}$.*

Similar to the proof of Lemma 2, we have

$$\begin{array}{cc} \; & ∥\hat{U}{\hat{U}}^{\prime }-UU^{\prime }{∥}_{2\to \infty }=\max_{i\in [n]}∥e_{i}^{\prime }\left(UU^{\prime }-\hat{U}{\hat{U}}^{\prime }\right){∥}_{F}\leq 2{∥U-\hat{U}\mathrm{sgn}\left(H\right)∥}_{2\to \infty }\\ \; & \leq C\frac{\theta_{\max}\sqrt{{\tilde{P}}_{\max}K}\left(\frac{\theta_{\max}\kappa \left(\Omega \right)}{\theta_{\min}}\sqrt{\frac{n}{K\lambda_{K}\left(\Pi^{\prime }\Pi \right)}}+\sqrt{\log(n)}\right)}{\sigma_{K}\left(\Omega \right)}\\ \; & \leq C\frac{\theta_{\max}\sqrt{{\tilde{P}}_{\max}K}\left(\frac{\theta_{\max}\kappa \left(\Omega \right)}{\theta_{\min}}\sqrt{\frac{n}{K\lambda_{K}\left(\Pi^{\prime }\Pi \right)}}+\sqrt{\log(n)}\right)}{\theta_{\min}^{2}\sigma_{K}\left(\tilde{P}\right)\lambda_{K}\left(\Pi^{\prime }\Pi \right)}, \end{array}$$

where the last inequality holds since $\sigma_{K}\left(\Omega \right)=\sigma_{K}\left(\Theta \Pi \tilde{P}\Pi^{\prime }\Theta \right)\geq \theta_{\min}^{2}\sigma_{K}\left(\Pi P\Pi^{\prime }\right)=\theta_{\min}^{2}\sigma_{K}\left(\Pi^{\prime }\Pi \tilde{P}\right)\geq \theta_{\min}^{2}\sigma_{K}\left(\tilde{P}\right)\sigma_{K}\left(\Pi^{\prime }\Pi \right)=\theta_{\min}^{2}\sigma_{K}\left(\tilde{P}\right)\lambda_{K}\left(\Pi^{\prime }\Pi \right)$. And this bound is $C\frac{\theta_{\max}\sqrt{{\tilde{P}}_{\max}\theta_{\max}{∥\theta ∥}_{1}\log\left(n\right)}}{\theta_{\min}^{3}\sigma_{K}\left(\tilde{P}\right)\lambda_{K}^{1.5}\left(\Pi^{\prime }\Pi \right)}$ if we use Theorem 4.2 of [65]. □

### Appendix D.4. Proof of Theorem 2

**Proof.** To prove this theorem, we follow similar procedures as Theorem 3.2 of [43]. For i∈[n], recall that $Z_{*}=Y_{*}J_{*}\equiv N_{U}^{-1}N_{M}\Pi ,{\hat{Z}}_{*}={\hat{Y}}_{*}{\hat{J}}_{*},\Pi \left(i,:\right)=\frac{Z(i,:)}{{∥Z\left(i,:\right)∥}_{1}}$ and ${\hat{\Pi }}_{*}\left(i,:\right)=\frac{{\hat{Z}}_{*}\left(i,:\right)}{∥{\hat{Z}}_{*}{\left(i,:\right)∥}_{1}}$, where $N_{M}$ and *M* are defined in the proof of Lemma 3 such that $U=\Theta M\equiv \Theta \Pi B_{*}$ and $N_{M}\left(i,i\right)=\frac{1}{{∥M\left(i,:\right)∥}_{F}}$, we have

$$\begin{array}{c} ∥e_{i}^{\prime }\left({\hat{\Pi }}_{*}-\Pi P_{*}\right){∥}_{1}\leq \frac{2∥e_{i}^{\prime }\left({\hat{Z}}_{*}-Z_{*}P_{*}\right){∥}_{1}}{∥e_{i}^{\prime }Z_{*}{∥}_{1}}\leq \frac{2\sqrt{K}{∥e_{i}^{\prime }\left({\hat{Z}}_{*}-Z_{*}P_{*}\right)∥}_{F}}{∥e_{i}^{\prime }Z_{*}{∥}_{1}}. \end{array}$$

Now, we provide a lower bound of $∥e_{i}^{\prime }Z_{*}{∥}_{1}$ as below

$$\begin{array}{cc} ∥e_{i}^{\prime }Z_{*}{∥}_{1} & =∥e_{i}^{\prime }N_{U}^{-1}N_{M}{\Pi ∥}_{1}=∥N_{U}^{-1}\left(i,i\right)e_{i}^{\prime }N_{M}{\Pi ∥}_{1}=N_{U}^{-1}\left(i,i\right){∥N_{M}\left(i,i\right)e_{i}^{\prime }\Pi ∥}_{1}=\frac{N_{M}\left(i,i\right)}{N_{U}\left(i,i\right)}\\ \; & ={∥U\left(i,:\right)∥}_{F}N_{M}\left(i,i\right)={∥U\left(i,:\right)∥}_{F}\frac{1}{{∥M\left(i,:\right)∥}_{F}}={∥U\left(i,:\right)∥}_{F}\frac{1}{∥e_{i}^{\prime }{M∥}_{F}}\\ \; & ={∥U\left(i,:\right)∥}_{F}\frac{1}{∥e_{i}^{\prime }\Theta^{-1}{U∥}_{F}}={∥U\left(i,:\right)∥}_{F}\frac{1}{∥\Theta^{-1}\left(i,i\right)e_{i}^{\prime }{U∥}_{F}}=\theta \left(i\right)\geq \theta_{\min}. \end{array}$$

Therefore, by Lemma A3, we have

$$\begin{array}{cc} ∥e_{i}^{\prime }\left({\hat{\Pi }}_{*}-\Pi P_{*}\right){∥}_{1} & \leq \frac{2\sqrt{K}{∥e_{i}^{\prime }\left({\hat{Z}}_{*}-Z_{*}P_{*}\right)∥}_{F}}{∥e_{i}^{\prime }Z_{*}{∥}_{1}}\leq \frac{2\sqrt{K}{∥e_{i}^{\prime }\left({\hat{Z}}_{*}-Z_{*}P_{*}\right)∥}_{F}}{\theta_{\min}}\\ \; & =O(\frac{\theta_{\max}^{15}K^{5}\varpi \kappa^{4.5}\left(\Pi^{\prime }\Pi \right)\lambda_{1}^{1.5}\left(\Pi^{\prime }\Pi \right)}{\theta_{\min}^{15}\pi_{\min}}). \end{array}$$

□

### Appendix D.5. Proof of Corollary 2

**Proof.** Under conditions of Corollary 2, we have

$$\begin{array}{c} \max_{i\in [n]}{∥e_{i}^{\prime }\left({\hat{\Pi }}_{*}-\Pi P_{*}\right)∥}_{1}=O\left(\frac{\theta_{\max}^{15}K^{5}\varpi \kappa^{4.5}\left(\Pi^{\prime }\Pi \right)\lambda_{1}^{1.5}\left(\Pi^{\prime }\Pi \right)}{\theta_{\min}^{15}\pi_{\min}}\right)=O\left(\frac{\theta_{\max}^{15}\varpi \sqrt{n}}{\theta_{\min}^{15}}\right). \end{array}$$

Under the conditions of Corollary 2, Lemma 5 gives $\varpi =O(\frac{\theta_{\max}\sqrt{{\tilde{P}}_{\max}\theta_{\max}{∥\theta ∥}_{1}\log\left(n\right)}}{\theta_{\min}^{3}\sigma_{K}\left(\tilde{P}\right)n^{1.5}})$, which gives that

$$\begin{array}{c} \max_{i\in [n]}{∥e_{i}^{\prime }\left({\hat{\Pi }}_{*}-\Pi P_{*}\right)∥}_{1}=O\left(\frac{\theta_{\max}^{15}\varpi \sqrt{n}}{\theta_{\min}^{15}}\right)=O\left(\frac{\theta_{\max}^{16}\sqrt{{\tilde{P}}_{\max}\theta_{\max}{∥\theta ∥}_{1}\log\left(n\right)}}{\theta_{\min}^{18}\sigma_{K}\left(\tilde{P}\right)n}\right). \end{array}$$

By basic algebra, this corollary follows. □

### Appendix D.6. Basic Properties of *Ω* under DCMM

**Lemma** **A1.**
*Under $DCMM_{n}\left(K,\tilde{P},\Pi ,\Theta \right)$, we have*

$$\begin{array}{cc} \; & {∥U\left(i,:\right)∥}_{F}\geq \frac{\theta_{\min}}{\theta_{\max}\sqrt{K\lambda_{1}\left(\Pi^{\prime }\Pi \right)}}\;\mathrm{for}\;i\in \left[n\right],\mathrm{and}\;\eta \geq \frac{\theta_{\min}^{4}\pi_{\min}}{\theta_{\max}^{4}K\lambda_{1}\left(\Pi^{\prime }\Pi \right)}, \end{array}$$

*where $\eta =\min_{k\in [K]}\left({\left(U_{*}\left(I,:\right)U_{*}^{\prime }\left(I,:\right)\right)}^{-1}1\right)\left(k\right)$.*

**Proof.** Since $I=U^{\prime }U=U^{\prime }\left(I,:\right)\Theta^{-1}\left(I,I\right)\Pi^{\prime }\Theta^{2}\Pi \Theta^{-1}\left(I,I\right)U\left(I,:\right)$ by the proof of Lemma 3, we have $(\left(\Theta^{-1}\left(I,I\right)U\left(I,:\right)\right){\left({\left(\Theta^{-1}\left(I,I\right)U\left(I,:\right)\right)}^{\prime }\right)}^{-1}=\Pi^{\prime }\Theta^{2}\Pi$, which gives that

$$\begin{array}{cc} \min_{k}{∥e_{k}^{\prime }\left(\Theta^{-1}\left(I,I\right)U\left(I,:\right)\right)∥}_{F}^{2} & =\min_{k}e_{k}^{\prime }\left(\Theta^{-1}\left(I,I\right)U\left(I,:\right)\right){\left(\Theta^{-1}\left(I,I\right)U\left(I,:\right)\right)}^{\prime }e_{k}\\ \; & \geq \min_{∥x∥=1}x^{\prime }\left(\Theta^{-1}\left(I,I\right)U\left(I,:\right)\right){\left(\Theta^{-1}\left(I,I\right)U\left(I,:\right)\right)}^{\prime }x\\ & =\lambda_{K}\left(\left(\Theta^{-1}\left(I,I\right)U\left(I,:\right)\right){\left(\Theta^{-1}\left(I,I\right)U\left(I,:\right)\right)}^{\prime }\right)=\frac{1}{\lambda_{1}\left(\Pi^{\prime }\Theta^{2}\Pi \right)}, \end{array}$$

where *x* is a K×1 vector whose $l_{2}$ norm is 1. Then, for i∈[n], we have

$$\begin{array}{cc} {∥U\left(i,:\right)∥}_{F} & =∥\theta_{i}\Pi \left(i,:\right)\Theta^{-1}{\left(I,I\right)U\left(I,:\right)∥}_{F}=\theta_{i}{∥\Pi \left(i,:\right)\Theta^{-1}\left(I,I\right)U\left(I,:\right)∥}_{F}\\ \; & \geq \theta_{i}\min_{i}{∥\Pi \left(i,:\right)∥}_{F}\min_{i}{∥e_{i}^{\prime }\left(\Theta^{-1}\left(I,I\right)U\left(I,:\right)\right)∥}_{F}\\ \; & \geq \theta_{i}\min_{i}{∥e_{i}^{\prime }\left(\Theta^{-1}\left(I,I\right)U\left(I,:\right)\right)∥}_{F}/\sqrt{K}\\ & \geq \frac{\theta_{i}}{\sqrt{K\lambda_{1}\left(\Pi^{\prime }\Theta^{2}\Pi \right)}}\geq \frac{\theta_{\min}}{\theta_{\max}\sqrt{K\lambda_{1}\left(\Pi^{\prime }\Pi \right)}}, \end{array}$$

where we use the fact that $\min_{i}{∥\Pi \left(i,:\right)∥}_{F}\geq \frac{1}{\sqrt{K}}$ since $\sum_{k=1}^{K}\Pi \left(i,k\right)=1$ and all entries of Π are non-negative. Since $U_{*}=N_{U}U$, we have

$$\begin{array}{cc} {\left(U_{*}\left(I,:\right)U_{*}^{\prime }\left(I,:\right)\right)}^{-1} & =N_{U}^{-1}\left(I,I\right)\Theta^{-1}\left(I,I\right)\Pi^{\prime }\Theta^{2}\Pi \Theta^{-1}\left(I,I\right)N_{U}^{-1}\left(I,I\right)\\ \; & \geq \frac{\theta_{\min}^{2}}{\theta_{\max}^{2}N_{U,\max}^{2}}\Pi^{\prime }\Pi \geq \frac{\theta_{\min}^{4}}{\theta_{\max}^{4}K\lambda_{1}\left(\Pi^{\prime }\Pi \right)}\Pi^{\prime }\Pi , \end{array}$$

where we set $N_{U,\max}=\max_{i\in [n]}N_{U}\left(i,i\right)$ and we use the fact that $N_{U},\Theta$ are diagonal matrices and $N_{U,\max}\leq \frac{\theta_{\max}\sqrt{K\lambda_{1}\left(\Pi^{\prime }\Pi \right)}}{\theta_{\min}}$. Then we have

$$\begin{array}{cc} \; & \eta =\min_{k\in [K]}\left({\left(U_{*}\left(I,:\right)U_{*}^{\prime }\left(I,:\right)\right)}^{-1}1\right)\left(k\right)\geq \frac{\theta_{\min}^{4}}{\theta_{\max}^{4}K\lambda_{1}\left(\Pi^{\prime }\Pi \right)}\min_{k\in [K]}e_{k}^{\prime }\Pi^{\prime }\Pi 1\\ \; & =\frac{\theta_{\min}^{4}}{\theta_{\max}^{4}K\lambda_{1}\left(\Pi^{\prime }\Pi \right)}\min_{k\in [K]}e_{k}^{\prime }\Pi^{\prime }1=\frac{\theta_{\min}^{4}\pi_{\min}}{\theta_{\max}^{4}K\lambda_{1}\left(\Pi^{\prime }\Pi \right)}. \end{array}$$

□

### Appendix D.7. Bounds between Ideal SVM-cone-DCMMSB and SVM-cone-DCMMSB

The next lemma focuses on the 2nd step of SVM-cone-DCMMSB and is the cornerstone to characterize the behaviors of SVM-cone-DCMMSB.

**Lemma** **A2.**
*Under $DCMM_{n}\left(K,\tilde{P},\Pi ,\Theta \right)$, when conditions of Lemma 5 hold, there exists a permutation matrix $P_{*}\in R^{K\times K}$ such that with probability at least $1-o(n^{-\alpha })$, we have*

$$\begin{array}{c} \max_{1\leq k\leq K}{∥e_{k}^{\prime }\left({\hat{U}}_{*,2}\left({\hat{I}}_{*},:\right)-P_{*}^{\prime }U_{*,2}\left(I,:\right)\right)∥}_{F}=O\left(\frac{K^{3}\theta_{\max}^{11}\varpi \kappa^{3}\left(\Pi^{\prime }\Pi \right)\lambda_{1}^{1.5}\left(\Pi^{\prime }\Pi \right)}{\theta_{\min}^{11}\pi_{\min}}\right), \end{array}$$

*where $U_{*,2}=U_{*}U^{\prime },{\hat{U}}_{*,2}={\hat{U}}_{*}{\hat{U}}^{\prime }$, i.e., $U_{*,2},{\hat{U}}_{*,2}$ are the row-normalized versions of $UU^{\prime }$ and $\hat{U}{\hat{U}}^{\prime }$, respectively.*

**Proof.** Lemma G.1. of [43] says that using ${\hat{U}}_{*,2}$ as input of the SVM-cone algorithm returns the same result as using ${\hat{U}}_{*}$ as the input. By Lemma F.1 of [43], there exists a permutation matrix $P_{*}\in R^{K\times K}$ such that

$$\begin{array}{c} \max_{k\in [K]}{∥e_{k}^{\prime }\left({\hat{U}}_{*,2}\left({\hat{I}}_{*},:\right)-P_{*}^{\prime }U_{*,2}\left(I,:\right)\right)∥}_{F}=O\left(\frac{\sqrt{K}\zeta \epsilon_{*}}{\lambda_{K}^{1.5}\left(U_{*,2}\left(I,:\right)\right)U_{*,2}^{\prime }\left(I,:\right)}\right), \end{array}$$

where $\zeta \leq \frac{4K}{\eta \lambda_{K}^{1.5}\left(U_{*,2}\left(I,:\right)U_{*,2}^{\prime }\left(I,:\right)\right)}=O\left(\frac{K}{\eta \lambda_{K}^{1.5}\left(U_{*}\left(I,:\right)U_{*}^{\prime }\left(I,:\right)\right)}\right),\epsilon_{*}=\max_{i\in [n]}{∥{\hat{U}}_{*,2}\left(i,:\right)-U_{*,2}\left(i,:\right)∥}_{F}$ and $\eta =\min_{1\leq k\leq K}\left({\left(U_{*}\left(I,:\right)U_{*}^{\prime }\left(I,:\right)\right)}^{-1}1\right)\left(k\right)$. Next we give upper bound of $\epsilon_{*}$.

$$\begin{array}{cc} & ∥{\hat{U}}_{*,2}\left(i,:\right)-U_{*,2}{\left(i,:\right)∥}_{F}={∥\frac{{\hat{U}}_{2}\left(i,:\right)∥U_{2}{\left(i,:\right)∥}_{F}-U_{2}\left(i,:\right){∥{\hat{U}}_{2}\left(i,:\right)∥}_{F}}{∥{\hat{U}}_{2}{\left(i,:\right)∥}_{F}{∥U_{2}\left(i,:\right)∥}_{F}}∥}_{F}\leq \frac{2∥{\hat{U}}_{2}\left(i,:\right)-U_{2}{\left(i,:\right)∥}_{F}}{∥U_{2}{\left(i,:\right)∥}_{F}}\\ \; & \leq \frac{2∥{\hat{U}}_{2}-U_{2}{∥}_{2\to \infty }}{∥U_{2}{\left(i,:\right)∥}_{F}}\leq \frac{2\varpi }{∥U_{2}{\left(i,:\right)∥}_{F}}=\frac{2\varpi }{∥\left(UU^{\prime }\right)\left(i,:\right){∥}_{F}}=\frac{2\varpi }{∥U\left(i,:\right)U^{\prime }{∥}_{F}}=\frac{2\varpi }{{∥U\left(i,:\right)∥}_{F}}\\ \; & \leq \frac{2\theta_{\max}\varpi \sqrt{K\lambda_{1}\left(\Pi^{\prime }\Pi \right)}}{\theta_{\min}}, \end{array}$$

where the last inequality holds by Lemma A1. Then, we have $\epsilon_{*}=O\left(\frac{\theta_{\max}\varpi \sqrt{K\lambda_{1}\left(\Pi^{\prime }\Pi \right)}}{\theta_{\min}}\right)$. By Lemma H.2. of [43], $\lambda_{K}\left(U_{*}\left(I,:\right)U_{*}^{\prime }\left(I,:\right)\right)\geq \frac{\theta_{\min}^{2}\kappa^{-1}\left(\Pi^{\prime }\Pi \right)}{\theta_{\max}^{2}}$. By the lower bound of η given in Lemma A1, we have

$$\begin{array}{c} \max_{k\in [K]}{∥e_{k}^{\prime }\left({\hat{U}}_{*,2}\left({\hat{I}}_{*},:\right)-P_{*}^{\prime }U_{*,2}\left(I,:\right)\right)∥}_{F}=O\left(\frac{K^{3}\theta_{\max}^{11}\varpi \kappa^{3}\left(\Pi^{\prime }\Pi \right)\lambda_{1}^{1.5}\left(\Pi^{\prime }\Pi \right)}{\theta_{\min}^{11}\pi_{\min}}\right). \end{array}$$

□

Next the lemma focuses on the 3rd step of SVM-cone-DCMMSB and bounds $\max_{i\in [n]}{∥e_{i}^{\prime }\left({\hat{Z}}_{*}-Z_{*}P_{*}\right)∥}_{F}$.

**Lemma** **A3.**
*Under $DCMM_{n}\left(K,\tilde{P},\Pi ,\Theta \right)$, when the conditions of Lemma 5 hold, with a probability of at least $1-o(n^{-\alpha })$, we have*

$$\begin{array}{c} \max_{i\in [n]}{∥e_{i}^{\prime }\left({\hat{Z}}_{*}-Z_{*}P_{*}\right)∥}_{F}=O\left(\frac{\theta_{\max}^{15}K^{4.5}\varpi \kappa^{4.5}\left(\Pi^{\prime }\Pi \right)\lambda_{1}^{1.5}\left(\Pi^{\prime }\Pi \right)}{\theta_{\min}^{14}\pi_{\min}}\right). \end{array}$$

**Proof.** For i∈[n], since $Z_{*}=Y_{*}J_{*},{\hat{Z}}_{*}={\hat{Y}}_{*}{\hat{J}}_{*}$ and $J_{*},{\hat{J}}_{*}$ are diagonal matrices, we have

$$\begin{array}{cc} & ∥e_{i}^{\prime }\left({\hat{Z}}_{*}-Z_{*}P_{*}\right){∥}_{F}=∥e_{i}^{\prime }\left(\max\left(0,{\hat{Y}}_{*}{\hat{J}}_{*}\right)-Y_{*}J_{*}P_{*}\right){∥}_{F}\leq {∥e_{i}^{\prime }\left({\hat{Y}}_{*}{\hat{J}}_{*}-Y_{*}J_{*}P_{*}\right)∥}_{F}\\ \; & =∥e_{i}^{\prime }\left({\hat{Y}}_{*}-Y_{*}P_{*}\right){\hat{J}}_{*}+e_{i}^{\prime }Y_{*}P_{*}\left({\hat{J}}_{*}-P_{*}^{\prime }J_{*}P_{*}\right){∥}_{F}\\ \; & \leq ∥e_{i}^{\prime }\left({\hat{Y}}_{*}-Y_{*}P_{*}\right){∥}_{F}∥{\hat{J}}_{*}∥+∥e_{i}^{\prime }Y_{*}P_{*}{∥}_{F}∥{\hat{J}}_{*}-P_{*}^{\prime }J_{*}P_{*}∥\\ \; & =∥e_{i}^{\prime }\left({\hat{Y}}_{*}-Y_{*}P_{*}\right){∥}_{F}∥{\hat{J}}_{*}∥+∥e_{i}^{\prime }Y_{*}{∥}_{F}∥{\hat{J}}_{*}-P_{*}^{\prime }J_{*}P_{*}∥\\ \; & =∥e_{i}^{\prime }\left({\hat{Y}}_{*}-Y_{*}P_{*}\right){∥}_{F}∥{\hat{J}}_{*}∥+∥e_{i}^{\prime }Y_{*}{∥}_{F}∥J_{*}-P_{*}{\hat{J}}_{*}P_{*}^{\prime }∥. \end{array}$$

Therefore, the bound of $∥e_{i}^{\prime }\left({\hat{Z}}_{*}-Z_{*}P_{*}\right){∥}_{F}$ can be obtained as long as we bound $∥e_{i}^{\prime }\left({\hat{Y}}_{*}-Y_{*}P_{*}\right){∥}_{F},∥{\hat{J}}_{*}∥,∥e_{i}^{\prime }Y_{*}{∥}_{F}$ and $∥J_{*}-P_{*}{\hat{J}}_{*}P_{*}^{\prime }∥$. We bound the four terms as below:

- We bound $∥e_{i}^{\prime }\left({\hat{Y}}_{*}-Y_{*}P_{*}\right){∥}_{F}$ first. Set $U_{*}\left(I,:\right)=B_{*},{\hat{U}}_{*}\left({\hat{I}}_{*},:\right)={\hat{B}}_{*},U_{*,2}\left(I,:\right)=B_{2*},{\hat{U}}_{*,2}\left({\hat{I}}_{*},:\right)={\hat{B}}_{2*}$ for convenience. For i∈[n], we have
  $$\begin{array}{cc} & ∥e_{i}^{\prime }\left({\hat{Y}}_{*}-Y_{*}P_{*}\right){∥}_{F}={∥e_{i}^{\prime }\left(\hat{U}{\hat{B}}_{*}^{\prime }{\left({\hat{B}}_{*}{\hat{B}}_{*}^{\prime }\right)}^{-1}-UB_{*}^{\prime }{\left(B_{*}B_{*}^{\prime }\right)}^{-1}P_{*}\right)∥}_{F}\\ & =∥e_{i}^{\prime }\left(\hat{U}-U\left(U^{\prime }\hat{U}\right)\right){\hat{B}}_{*}^{\prime }{\left({\hat{B}}_{*}{\hat{B}}_{*}^{\prime }\right)}^{-1}+e_{i}^{\prime }(U\left(U^{\prime }\hat{U}\right){\hat{B}}_{*}^{\prime }{\left({\hat{B}}_{*}{\hat{B}}_{*}^{\prime }\right)}^{-1}\\ \; & \;\;\;\;-U\left(U^{\prime }\hat{U}\right){\left(P_{*}^{\prime }\left(B_{*}B_{*}^{\prime }\right){\left(B_{*}^{\prime }\right)}^{-1}\left(U^{\prime }\hat{U}\right)\right)}^{-1}{)∥}_{F}\\ & \leq ∥e_{i}^{\prime }\left(\hat{U}-U\left(U^{\prime }\hat{U}\right)\right){\hat{B}}_{*}^{\prime }{\left({\hat{B}}_{*}{\hat{B}}_{*}^{\prime }\right)}^{-1}{∥}_{F}+∥e_{i}^{\prime }U\left(U^{\prime }\hat{U}\right)({\hat{B}}_{*}^{\prime }{\left({\hat{B}}_{*}{\hat{B}}_{*}^{\prime }\right)}^{-1}\\ \; & \;\;\;\;-{\left(P_{*}^{\prime }\left(B_{*}B_{*}^{\prime }\right){\left(B_{*}^{\prime }\right)}^{-1}\left(U^{\prime }\hat{U}\right)\right)}^{-1}{)∥}_{F}\\ \; & \leq ∥e_{i}^{\prime }\left(\hat{U}-U\left(U^{\prime }\hat{U}\right)\right){∥}_{F}∥{\hat{B}}_{*}^{-1}{∥}_{F}+{∥e_{i}^{\prime }U\left(U^{\prime }\hat{U}\right)\left({\hat{B}}_{*}^{\prime }{\left({\hat{B}}_{*}{\hat{B}}_{*}^{\prime }\right)}^{-1}-{\left(P_{*}^{\prime }\left(B_{*}B_{*}^{\prime }\right){\left(B_{*}^{\prime }\right)}^{-1}\left(U^{\prime }\hat{U}\right)\right)}^{-1}\right)∥}_{F}\\ & \leq \sqrt{K}∥e_{i}^{\prime }\left(\hat{U}-U\left(U^{\prime }\hat{U}\right)\right){∥}_{F}/\sqrt{\lambda_{K}\left({\hat{B}}_{*}{\hat{B}}_{*}^{\prime }\right)}+{∥e_{i}^{\prime }U\left(U^{\prime }\hat{U}\right)\left({\hat{B}}_{*}^{-1}-{\left(P_{*}^{\prime }B_{*}\left(U^{\prime }\hat{U}\right)\right)}^{-1}\right)∥}_{F}\\ \; & \overset{\left(i\right)}{=}\sqrt{K}∥e_{i}^{\prime }\left(\hat{U}{\hat{U}}^{\prime }-UU^{\prime }\right)\hat{U}{∥}_{F}O\left(\frac{\theta_{\max}\sqrt{\kappa (\Pi^{\prime }\Pi )}}{\theta_{\min}}\right)+{∥e_{i}^{\prime }U\left(U^{\prime }\hat{U}\right)\left({\hat{B}}_{*}^{-1}-{\left(P_{*}^{\prime }B_{*}\left(U^{\prime }\hat{U}\right)\right)}^{-1}\right)∥}_{F}\\ \; & \leq \sqrt{K}∥e_{i}^{\prime }\left(\hat{U}{\hat{U}}^{\prime }-UU^{\prime }\right){∥}_{F}O\left(\frac{\theta_{\max}\sqrt{\kappa (\Pi^{\prime }\Pi )}}{\theta_{\min}}\right)+{∥e_{i}^{\prime }U\left(U^{\prime }\hat{U}\right)\left({\hat{B}}_{*}^{-1}-{\left(P_{*}^{\prime }B_{*}\left(U^{\prime }\hat{U}\right)\right)}^{-1}\right)∥}_{F}\\ \; & \leq \sqrt{K}\varpi O\left(\frac{\theta_{\max}\sqrt{\kappa (\Pi^{\prime }\Pi )}}{\theta_{\min}}\right)+{∥e_{i}^{\prime }U\left(U^{\prime }\hat{U}\right)\left({\hat{B}}_{*}^{-1}-{\left(P_{*}^{\prime }B_{*}\left(U^{\prime }\hat{U}\right)\right)}^{-1}\right)∥}_{F}\\ & =O\left(\varpi \frac{\theta_{\max}\sqrt{K\kappa (\Pi^{\prime }\Pi )}}{\theta_{\min}}\right)+{∥e_{i}^{\prime }U\left(U^{\prime }\hat{U}\right)\left({\hat{B}}_{*}^{-1}-{\left(P_{*}^{\prime }B_{*}\left(U^{\prime }\hat{U}\right)\right)}^{-1}\right)∥}_{F}, \end{array}$$
  where we have used similar idea in the proof of Lemma VII.3 in [44] such that we apply $O(\frac{1}{\lambda_{K}\left(B_{*}B_{*}^{\prime }\right)})$ to estimate $\frac{1}{\lambda_{K}\left({\hat{B}}_{*}{\hat{B}}_{*}^{\prime }\right)}$.
  Now we aim to bound $∥e_{i}^{\prime }U\left(U^{\prime }\hat{U}\right)\left({\hat{B}}_{*}^{-1}-{\left(P_{*}^{\prime }B_{*}\left(U^{\prime }\hat{U}\right)\right)}^{-1}\right){∥}_{F}$. For convenience, set $T=U^{\prime }\hat{U},S=P_{*}^{\prime }B_{*}T$. We have
  $$\begin{array}{cc} & ∥e_{i}^{\prime }U\left(U^{\prime }\hat{U}\right)\left({\hat{B}}_{*}^{-1}-{\left(P_{*}^{\prime }B_{*}\left(U^{\prime }\hat{U}\right)\right)}^{-1}\right){∥}_{F}={∥e_{i}^{\prime }UTS^{-1}\left(S-{\hat{B}}_{*}\right){\hat{B}}_{*}^{-1}∥}_{F}\\ & \leq ∥e_{i}^{\prime }UTS^{-1}\left(S-{\hat{B}}_{*}\right){∥}_{F}∥{\hat{B}}_{*}^{-1}{∥}_{F}\leq {∥e_{i}^{\prime }UTS^{-1}\left(S-{\hat{B}}_{*}\right)∥}_{F}\frac{\sqrt{K}}{|\lambda_{K}\left({\hat{B}}_{*}\right)|}\\ & =∥e_{i}^{\prime }UTS^{-1}\left(S-{\hat{B}}_{*}\right){∥}_{F}\frac{\sqrt{K}}{\sqrt{\lambda_{K}\left({\hat{B}}_{*}{\hat{B}}_{*}^{\prime }\right)}}\leq {∥e_{i}^{\prime }UTS^{-1}\left(S-{\hat{B}}_{*}\right)∥}_{F}O\left(\frac{\theta_{\max}\sqrt{K\kappa (\Pi^{\prime }\Pi )}}{\theta_{\min}}\right)\\ & =∥e_{i}^{\prime }UTT^{-1}B_{*}^{\prime }{\left(B_{*}B_{*}^{\prime }\right)}^{-1}P_{*}\left(S-{\hat{B}}_{*}\right){∥}_{F}O\left(\frac{\theta_{\max}\sqrt{K\kappa (\Pi^{\prime }\Pi )}}{\theta_{\min}}\right)\\ & =∥e_{i}^{\prime }UB_{*}^{\prime }{\left(B_{*}B_{*}^{\prime }\right)}^{-1}P_{*}\left(S-{\hat{B}}_{*}\right){∥}_{F}O\left(\frac{\theta_{\max}\sqrt{K\kappa (\Pi^{\prime }\Pi )}}{\theta_{\min}}\right)\\ & =∥e_{i}^{\prime }Y_{*}P_{*}\left(S-{\hat{B}}_{*}\right){∥}_{F}O\left(\frac{\theta_{\max}\sqrt{K\lambda_{1}\left(\Pi^{\prime }\Pi \right)}}{\theta_{\min}}\right)\leq ∥e_{i}^{\prime }Y_{*}{∥}_{F}{∥S-{\hat{B}}_{*}∥}_{F}O\left(\frac{\theta_{\max}\sqrt{K\lambda_{1}\left(\Pi^{\prime }\Pi \right)}}{\theta_{\min}}\right)\\ \; & \overset{\mathrm{By}\;\mathrm{Equation}\;(A3)}{\leq }\frac{\theta_{\max}^{2}\sqrt{K\lambda_{1}\left(\Pi^{\prime }\Pi \right)}}{\theta_{\min}^{2}\lambda_{K}\left(\Pi^{\prime }\Pi \right)}\max_{1\leq k\leq K}{∥e_{k}^{\prime }\left(S-{\hat{B}}_{*}\right)∥}_{F}O\left(\frac{\theta_{\max}K\sqrt{\kappa (\Pi^{\prime }\Pi )}}{\theta_{\min}}\right)\\ & =\max_{1\leq k\leq K}{∥e_{k}^{\prime }\left({\hat{B}}_{*}-P_{*}^{\prime }B_{*}U^{\prime }\hat{U}\right)∥}_{F}O\left(\frac{\theta_{\max}^{3}K^{1.5}\kappa \left(\Pi^{\prime }\Pi \right)}{\theta_{\min}^{3}\sqrt{\lambda_{K}\left(\Pi^{\prime }\Pi \right)}}\right)\\ \; & =\max_{1\leq k\leq K}{∥e_{k}^{\prime }\left({\hat{B}}_{*}{\hat{U}}^{\prime }-P_{*}^{\prime }B_{*}U^{\prime }\right)\hat{U}∥}_{F}O\left(\frac{\theta_{\max}^{3}K^{1.5}\kappa \left(\Pi^{\prime }\Pi \right)}{\theta_{\min}^{3}\sqrt{\lambda_{K}\left(\Pi^{\prime }\Pi \right)}}\right)\\ & \leq \max_{1\leq k\leq K}{∥e_{k}^{\prime }\left({\hat{B}}_{*}{\hat{U}}^{\prime }-P^{\prime }B_{*}U^{\prime }\right)∥}_{F}O\left(\frac{\theta_{\max}^{3}K^{1.5}\kappa \left(\Pi^{\prime }\Pi \right)}{\theta_{\min}^{3}\sqrt{\lambda_{K}\left(\Pi^{\prime }\Pi \right)}}\right)\\ \; & =\max_{1\leq k\leq K}{∥e_{k}^{\prime }\left({\hat{B}}_{2*}-P_{*}^{\prime }B_{2*}\right)∥}_{F}O\left(\frac{\theta_{\max}^{3}K^{1.5}\kappa \left(\Pi^{\prime }\Pi \right)}{\theta_{\min}^{3}\sqrt{\lambda_{K}\left(\Pi^{\prime }\Pi \right)}}\right)\\ \; & \overset{\mathrm{By}\;\mathrm{Lemma}A2\;}{=}O\left(\frac{K^{4.5}\theta_{\max}^{14}\varpi \kappa^{4.5}\left(\Pi^{\prime }\Pi \right)\lambda_{1}\left(\Pi^{\prime }\Pi \right)}{\theta_{\min}^{14}\pi_{\min}}\right). \end{array}$$
  Then, we have
  $$\begin{array}{cc} ∥e_{i}^{\prime }\left({\hat{Y}}_{*}-Y_{*}P_{*}\right){∥}_{F} & \leq O\left(\varpi \frac{\theta_{\max}\sqrt{K\kappa (\Pi^{\prime }\Pi )}}{\theta_{\min}}\right)+∥e_{i}^{\prime }U\left(U^{\prime }\hat{U}\right){\left({\hat{B}}_{*}^{-1}-\left(P_{*}^{\prime }B_{*}U^{\prime }\hat{U}\right)\right)}^{-1}{)∥}_{F}\\ \; & \leq O\left(\varpi \frac{\theta_{\max}\sqrt{K\kappa (\Pi^{\prime }\Pi )}}{\theta_{\min}}\right)+O\left(\frac{K^{4.5}\theta_{\max}^{14}\varpi \kappa^{4.5}\left(\Pi^{\prime }\Pi \right)\lambda_{1}\left(\Pi^{\prime }\Pi \right)}{\theta_{\min}^{14}\pi_{\min}}\right)\\ \; & =O(\frac{K^{4.5}\theta_{\max}^{14}\varpi \kappa^{4.5}\left(\Pi^{\prime }\Pi \right)\lambda_{1}\left(\Pi^{\prime }\Pi \right)}{\theta_{\min}^{14}\pi_{\min}}). \end{array}$$
- for $∥e_{i}^{\prime }Y_{*}{∥}_{F}$, since $Y_{*}=UU_{*}^{-1}\left(I,:\right)$, we have
  (A3) $$\begin{array}{c} ∥e_{i}^{\prime }Y_{*}{∥}_{F}\leq {∥U\left(i,:\right)∥}_{F}{∥U_{*}^{-1}\left(I,:\right)∥}_{F}\leq \frac{\sqrt{K}{∥U\left(i,:\right)∥}_{F}}{\sqrt{\lambda_{K}\left(U_{*}\left(I,:\right)U_{*}^{\prime }\left(I,:\right)\right)}}\leq \frac{\theta_{\max}^{2}\sqrt{K\lambda_{1}\left(\Pi^{\prime }\Pi \right)}}{\theta_{\min}^{2}\lambda_{K}\left(\Pi^{\prime }\Pi \right)}. \end{array}$$
- for $∥{\hat{J}}_{*}∥$, recall that ${\hat{J}}_{*}=\sqrt{\mathrm{diag}({\hat{U}}_{*}\left({\hat{I}}_{*},:\right)\hat{\Lambda }{\hat{U}}_{*}^{\prime }\left({\hat{I}}_{*},:\right))}$, we have
  $$\begin{array}{cc} ∥{\hat{J}}_{*}{∥}^{2} & =\max_{k\in [K]}{\hat{J}}_{*}^{2}\left(k,k\right)=\max_{k\in [K]}e_{k}^{\prime }{\hat{U}}_{*}\left({\hat{I}}_{*},:\right)\hat{\Lambda }{\hat{U}}_{*}^{\prime }\left({\hat{I}}_{*},:\right)e_{k}\\ \; & =\max_{k\in [K]}∥e_{k}^{\prime }{\hat{U}}_{*}\left({\hat{I}}_{*},:\right)\hat{\Lambda }{\hat{U}}_{*}^{\prime }\left({\hat{I}}_{*},:\right)e_{k}∥\\ \; & \leq \max_{k\in [K]}∥e_{k}^{\prime }{\hat{U}}_{*}\left({\hat{I}}_{*},:\right){∥}^{2}∥\hat{\Lambda }∥\leq \max_{k\in [K]}∥e_{k}^{\prime }{\hat{U}}_{*}\left({\hat{I}}_{*},:\right){∥}_{F}^{2}∥\hat{\Lambda }∥=∥\hat{\Lambda }∥, \end{array}$$
  where we have used the fact that $∥{\hat{U}}_{*}{\left(i,:\right)∥}_{F}=1$ for i∈[n] in the last equality. Since we need $\sigma_{K}\left(\Omega \right)\geq C\theta_{\max}\sqrt{{\tilde{P}}_{\max}n\log\left(n\right)}\geq C∥A-\Omega ∥$ in the proof of Lemma 5, we have $∥\hat{\Lambda }∥=∥A∥=∥A-\Omega +\Omega ∥\leq ∥A-\Omega ∥+∥\Omega ∥\leq \sigma_{K}\left(\Omega \right)+∥\Omega ∥\leq 2∥\Omega ∥=2∥\Theta \Pi \tilde{P}\Pi^{\prime }\Theta ∥\leq 2C\theta_{\max}^{2}{\tilde{P}}_{\max}\lambda_{1}\left(\Pi^{\prime }\Pi \right)=O\left(\theta_{\max}^{2}{\tilde{P}}_{\max}\lambda_{1}\left(\Pi^{\prime }\Pi \right)\right)$. Then we have
  $$\begin{array}{c} ∥{\hat{J}}_{*}∥=O\left(\theta_{\max}\sqrt{{\tilde{P}}_{\max}\lambda_{1}\left(\Pi^{\prime }\Pi \right)}\right). \end{array}$$
- for $∥J_{*}-P_{*}{\hat{J}}_{*}P_{*}^{\prime }∥$, we provide some simple facts first: $∥\hat{\Lambda }∥=∥A∥,∥\Lambda ∥=∥\Omega ∥,\Omega =U\Lambda U^{\prime },\tilde{A}=\hat{U}\hat{\Lambda }{\hat{U}}^{\prime },∥\hat{U}∥=1,∥U∥=1,∥e_{k}^{\prime }P_{*}{\hat{B}}_{2*}∥=∥{\hat{B}}_{2*}e_{k}∥=∥e_{k}^{\prime }{\hat{B}}_{2*}∥\leq ∥e_{k}^{\prime }{\hat{B}}_{2*}{∥}_{F}=1$. Since $\tilde{A}$ is the best rank *K* approximation to *A* in the spectral norm, and therefore $∥\tilde{A}-A∥\leq ∥\Omega -A∥$ since $\Omega =U\Lambda U^{\prime }$ with rank *K* and Ω can also be viewed as a rank *K* approximation to *A*. This leads to $∥\Omega -\tilde{A}∥=∥\Omega -A+A-\tilde{A}∥\leq 2∥A-\Omega ∥$. By Lemma H.2 [43], $∥B_{*}∥=∥U_{*}\left(I,:\right)∥=\sqrt{\lambda_{1}\left(U_{*}\left(I,:\right)U_{*}^{\prime }\left(I,:\right)\right)}\leq \sqrt{\kappa (\Pi^{\prime }\Theta^{2}\Pi )}\leq \frac{\theta_{\max}\kappa^{0.5}\left(\Pi^{\prime }\Pi \right)}{\theta_{\min}}$. $∥A∥=∥A-\Omega +\Omega ∥\leq ∥A-\Omega ∥+∥\Omega ∥\leq \sigma_{K}\left(\Omega \right)+∥\Omega ∥\leq 2∥\Omega ∥$ by the lower bound requirement of $\sigma_{K}\left(\Omega \right)$ in Lemma 5, and we also have $∥A-\Omega ∥\leq \sigma_{K}\left(\Omega \right)\leq ∥\Omega ∥$. For k∈[K], let $\tau_{k}=J_{*}\left(k,k\right),{\hat{\tau }}_{k}=\left(P_{*}{\hat{J}}_{*}P_{*}^{\prime }\right)\left(k,k\right)$ for convenience. Based on the above facts and Lemma A2, we have
  $$\begin{array}{cc} \; & \max_{k\in [K]}|\tau_{k}^{2}-{\hat{\tau }}_{k}^{2}|=\max_{k\in [K]}∥e_{k}^{\prime }U_{*}\left(I,:\right)\Lambda U_{*}^{\prime }\left(I,:\right)e_{k}-e_{k}^{\prime }P_{*}{\hat{U}}_{*}\left({\hat{I}}_{*},:\right)\hat{\Lambda }{\hat{U}}_{*}^{\prime }\left({\hat{I}}_{*},:\right)P_{*}^{\prime }e_{k}∥\\ \; & =\max_{k\in [K]}∥e_{k}^{\prime }B_{2*}U\Lambda U^{\prime }B_{2*}e_{k}-e_{k}^{\prime }P_{*}{\hat{B}}_{2*}\hat{U}\hat{\Lambda }{\hat{U}}^{\prime }{\hat{B}}_{2*}^{\prime }P_{*}^{\prime }e_{k}∥\\ \; & \leq ∥e_{k}^{\prime }\left(B_{2*}-P_{*}{\hat{B}}_{2*}\right)U\Lambda U^{\prime }B_{2*}^{\prime }e_{k}∥+∥e_{k}^{\prime }P_{*}{\hat{B}}_{2*}\left(U\Lambda U^{\prime }-\hat{U}\hat{\Lambda }{\hat{U}}^{\prime }\right)B_{2*}^{\prime }e_{k}∥\\ \; & \;\;\;+∥e_{k}^{\prime }P_{*}{\hat{B}}_{2*}\hat{U}\hat{\Lambda }{\hat{U}}^{\prime }\left(B_{2*}^{\prime }-{\hat{B}}_{2*}^{\prime }P_{*}^{\prime }\right)e_{k}∥\\ \; & \leq ∥e_{k}^{\prime }\left(B_{2*}-P_{*}{\hat{B}}_{2*}\right)∥∥U∥∥\Lambda ∥∥U^{\prime }∥∥B_{2*}^{\prime }e_{k}∥+∥e_{k}^{\prime }P_{*}{\hat{B}}_{2*}∥∥U\Lambda U^{\prime }-\hat{U}\hat{\Lambda }{\hat{U}}^{\prime }∥∥B_{2*}^{\prime }e_{k}∥\\ & \;\;\;+∥e_{k}^{\prime }P_{*}{\hat{B}}_{2*}∥∥\hat{U}∥∥\hat{\Lambda }∥∥{\hat{U}}^{\prime }∥∥\left(B_{2*}^{\prime }-{\hat{B}}_{2*}^{\prime }P_{*}^{\prime }\right)e_{k}∥\\ \; & \leq ∥e_{k}^{\prime }\left(B_{2*}-P_{*}{\hat{B}}_{2*}\right)∥∥\Lambda ∥∥B_{2*}^{\prime }e_{k}∥+∥U\Lambda U^{\prime }-\hat{U}\hat{\Lambda }{\hat{U}}^{\prime }∥∥B_{2*}^{\prime }e_{k}∥+∥\hat{\Lambda }∥∥\left(B_{2*}^{\prime }-{\hat{B}}_{2*}^{\prime }P_{*}^{\prime }\right)e_{k}∥\\ \; & =∥e_{k}^{\prime }\left(B_{2*}-P_{*}{\hat{B}}_{2*}\right)∥(∥\Omega ∥∥B_{2*}^{\prime }e_{k}∥+∥A∥)+∥\Omega -\tilde{A}∥∥B_{2*}^{\prime }e_{k}∥\\ \; & =∥e_{k}^{\prime }\left({\hat{B}}_{2*}-P_{*}^{\prime }B_{2*}\right)∥(∥\Omega ∥∥B_{2*}^{\prime }e_{k}∥+∥A∥)+∥\Omega -\tilde{A}∥∥B_{2*}^{\prime }e_{k}∥\\ \; & \leq ∥e_{k}^{\prime }\left({\hat{B}}_{2*}-P_{*}^{\prime }B_{2*}\right)∥(∥\Omega ∥∥B_{2*}^{\prime }e_{k}∥+∥A∥)+2∥A-\Omega ∥∥B_{2*}^{\prime }e_{k}∥\\ \; & =∥e_{k}^{\prime }\left({\hat{B}}_{2*}-P_{*}^{\prime }B_{2*}\right)∥(∥\Omega ∥∥UB_{*}^{\prime }e_{k}∥+∥A∥)+2∥A-\Omega ∥∥UB_{*}^{\prime }e_{k}∥\\ \; & \leq ∥e_{k}^{\prime }\left({\hat{B}}_{2*}-P_{*}^{\prime }B_{2*}\right)∥(∥\Omega ∥∥B_{*}^{\prime }e_{k}∥+∥A∥)+2∥A-\Omega ∥∥B_{*}^{\prime }e_{k}∥\\ \; & \leq ∥e_{k}^{\prime }\left({\hat{B}}_{2*}-P_{*}^{\prime }B_{2*}\right)∥(∥\Omega ∥∥B_{*}^{\prime }e_{k}∥+2∥\Omega ∥)+2∥\Omega ∥∥B_{*}^{\prime }e_{k}∥\\ \; & =∥e_{k}^{\prime }\left({\hat{B}}_{2*}-P_{*}^{\prime }B_{2*}\right)∥(∥B_{*}^{\prime }e_{k}∥+1)O\left(\theta_{\max}^{2}{\tilde{P}}_{\max}\lambda_{1}\left(\Pi^{\prime }\Pi \right)\right)+∥B_{*}^{\prime }e_{k}∥O\left(\theta_{\max}^{2}{\tilde{P}}_{\max}\lambda_{1}\left(\Pi^{\prime }\Pi \right)\right)\\ \; & \leq ∥e_{k}^{\prime }\left({\hat{B}}_{2*}-P_{*}^{\prime }B_{2*}\right)∥(∥B_{*}∥+1)O\left(\theta_{\max}^{2}{\tilde{P}}_{\max}\lambda_{1}\left(\Pi^{\prime }\Pi \right)\right)+∥B_{*}∥O\left(\theta_{\max}^{2}{\tilde{P}}_{\max}\lambda_{1}\left(\Pi^{\prime }\Pi \right)\right)\\ \; & \leq ∥e_{k}^{\prime }\left({\hat{B}}_{2*}-P_{*}^{\prime }B_{2*}\right)∥O\left(\theta_{\max}^{3}{\tilde{P}}_{\max}\kappa^{0.5}\left(\Pi^{\prime }\Pi \right)\lambda_{1}\left(\Pi^{\prime }\Pi \right)/\theta_{\min}\right)\\ \; & \;\;\;+O(\theta_{\max}^{3}{\tilde{P}}_{\max}\kappa^{0.5}\left(\Pi^{\prime }\Pi \right)\lambda_{1}\left(\Pi^{\prime }\Pi \right)/\theta_{\min})\\ \; & \leq ∥e_{k}^{\prime }\left({\hat{B}}_{2*}-P_{*}^{\prime }B_{2*}\right){∥}_{F}O\left(\theta_{\max}^{3}{\tilde{P}}_{\max}\kappa^{0.5}\left(\Pi^{\prime }\Pi \right)\lambda_{1}\left(\Pi^{\prime }\Pi \right)/\theta_{\min}\right)\\ \; & \;\;\;+O(\theta_{\max}^{3}{\tilde{P}}_{\max}\kappa^{0.5}\left(\Pi^{\prime }\Pi \right)\lambda_{1}\left(\Pi^{\prime }\Pi \right)/\theta_{\min})\\ \; & =O\left(\frac{K^{3}\theta_{\max}^{11}\varpi \kappa^{3}\left(\Pi^{\prime }\Pi \right)\lambda_{1}^{1.5}\left(\Pi^{\prime }\Pi \right)}{\theta_{\min}^{11}\pi_{\min}}\right)O\left(\theta_{\max}^{3}{\tilde{P}}_{\max}\kappa^{0.5}\left(\Pi^{\prime }\Pi \right)\lambda_{1}\left(\Pi^{\prime }\Pi \right)/\theta_{\min}\right)\\ \; & \;\;\;+O\left(\theta_{\max}^{3}{\tilde{P}}_{\max}\kappa^{0.5}\left(\Pi^{\prime }\Pi \right)\lambda_{1}\left(\Pi^{\prime }\Pi \right)/\theta_{\min}\right)=O\left(\frac{K^{3}\theta_{\max}^{14}{\tilde{P}}_{\max}\varpi \kappa^{3.5}\left(\Pi^{\prime }\Pi \right)\lambda_{1}^{2.5}\left(\Pi^{\prime }\Pi \right)}{\theta_{\min}^{12}\pi_{\min}}\right). \end{array}$$
  Recall that $J_{*}=N_{U}\left(I,I\right)\Theta \left(I,I\right)$, we have $∥J_{*}∥\leq N_{U,\max}\theta_{\max}\leq \frac{\theta_{\max}^{2}\sqrt{K\lambda_{1}\left(\Pi^{\prime }\Pi \right)}}{\theta_{\min}}$ where the last inequality holds by Lemma A1. Similarly, we have $J_{*}\left(k,k\right)\geq \theta_{\min}\min_{i\in [n]}\frac{1}{{∥U\left(i,:\right)∥}_{F}}\geq \frac{\theta_{\min}^{2}\sqrt{\lambda_{K}\left(\Pi^{\prime }\Pi \right)}}{\theta_{\max}}$ where the last inequality holds by the proof of Lemma 5. Then we have
  $$\begin{array}{cc} \; & ∥J_{*}-P_{*}{\hat{J}}_{*}P_{*}^{\prime }∥=\max_{k\in [K]}\left|{\hat{\tau }}_{k}-\tau_{k}\right|=\max_{k\in [K]}\frac{|{\hat{\tau }}_{k}^{2}-\tau_{k}^{2}|}{{\hat{\tau }}_{k}+\tau_{k}}\leq \max_{k\in [K]}\frac{|{\hat{\tau }}_{k}^{2}-\tau_{k}^{2}|}{\tau_{k}}\\ \; & \leq \frac{\theta_{\max}}{\theta_{\min}^{2}\sqrt{\lambda_{K}\left(\Pi^{\prime }\Pi \right)}}\max_{k\in [K]}\left|{\hat{\tau }}_{k}^{2}-\tau_{k}^{2}\right|=O\left(\frac{K^{3}\theta_{\max}^{15}{\tilde{P}}_{\max}\varpi \kappa^{3.5}\left(\Pi^{\prime }\Pi \right)\lambda_{1}^{2.5}\left(\Pi^{\prime }\Pi \right)}{\theta_{\min}^{14}\pi_{\min}\sqrt{\lambda_{K}\left(\Pi^{\prime }\Pi \right)}}\right). \end{array}$$
  Combining the above results, we have
  $$\begin{array}{cc} & ∥e_{i}^{\prime }\left({\hat{Z}}_{*}-Z_{*}P_{*}\right){∥}_{F}\leq ∥e_{i}^{\prime }\left({\hat{Y}}_{*}-Y_{*}P_{*}\right){∥}_{F}∥{\hat{J}}_{*}∥+∥e_{i}^{\prime }Y_{*}{∥}_{F}∥J_{*}-P_{*}{\hat{J}}_{*}P_{*}^{\prime }∥\\ \; & \leq O\left(\frac{K^{4.5}\theta_{\max}^{14}\varpi \kappa^{4.5}\left(\Pi^{\prime }\Pi \right)\lambda_{1}\left(\Pi^{\prime }\Pi \right)}{\theta_{\min}^{14}\pi_{\min}}\right)O\left(\theta_{\max}\sqrt{{\tilde{P}}_{\max}\lambda_{1}\left(\Pi^{\prime }\Pi \right)}\right)\\ \; & \;\;\;+\frac{\theta_{\max}^{2}\sqrt{K\lambda_{1}\left(\Pi^{\prime }\Pi \right)}}{\theta_{\min}^{2}\lambda_{K}\left(\Pi^{\prime }\Pi \right)}O\left(\frac{K^{3}\theta_{\max}^{15}{\tilde{P}}_{\max}\varpi \kappa^{3.5}\left(\Pi^{\prime }\Pi \right)\lambda_{1}^{2.5}\left(\Pi^{\prime }\Pi \right)}{\theta_{\min}^{14}\pi_{\min}\sqrt{\lambda_{K}\left(\Pi^{\prime }\Pi \right)}}\right)\\ \; & =O(\frac{\theta_{\max}^{15}K^{4.5}\varpi \kappa^{4.5}\left(\Pi^{\prime }\Pi \right)\lambda_{1}^{1.5}\left(\Pi^{\prime }\Pi \right)}{\theta_{\min}^{14}\pi_{\min}}). \end{array}$$

□
